# An atomic surface site interaction point description of non-covalent interactions†

**DOI:** 10.1039/d3sc05690b

**Published:** 2023-12-08

**Authors:** Maria Chiara Storer, Katarzyna J. Zator, Derek P. Reynolds, Christopher A. Hunter

**Affiliations:** a Yusuf Hamied Department of Chemistry, University of Cambridge Lensfield Road Cambridge CB2 1EW UK herchelsmith.orgchem@ch.cam.ac.uk

## Abstract

Molecular electrostatic potential surfaces (MEPS) calculated using density functional theory have been used to develop a simplified description of the non-covalent interaction properties of organic molecules. The Atomic Interaction Point (AIP) model introduced here represents an evolution of the Surface Site Interaction Point (SSIP) model described previously, in which a molecule is represented by a discrete set of interaction points that define sites of interaction with other molecules. The interaction sites are described by interaction parameters that are equivalent to the experimentally determined H-bond donor and acceptor parameters *α* and *β*. By using high electron density MEPS that lie inside the van der Waals surface, it is possible to obtain accurate interaction parameters and locations for polar sites (s-holes, H-bond donors and acceptors), which are identified as local maxima and minima on the MEPS. For non-polar sites that represent π-systems and halogens, an approach based on molecular orbitals was used to assign the locations of the AIPs, and the interaction parameters were obtained using a lower electron density MEPS that lies close to the van der Waals surface. The AIP descriptions can be implemented directly in the Surface Site Interaction Point Model for Liquids at Equilibrium (SSIMPLE) to calculate solvation free energies, and the free energy of transfer of 1504 compounds from *n*-hexadecane to water was predicted with a root mean square error of 5 kJ mol^−1^. AIPs also provide a useful tool for mapping non-covalent interactions in intermolecular complexes, and examples are provided showing how X-ray crystal structures can be converted into AIP interaction maps that allow quantification of the free energy contributions of both polar and non-polar interactions to the stabilities of complexes in solution.

## Abstract 2

Atomic interaction points provide a comprehensive description of the non-covalent interaction properties of organic compounds and can be used to predict free energy contributions to intermolecular complexation including the effects of desolvation.

## Graphical abstract

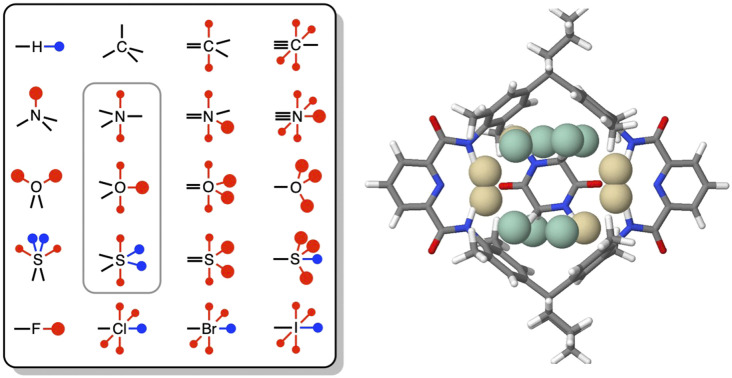

## Introduction

Intermolecular interactions determine the functional properties of most organic molecules, and quantitative predictive models for the relationship between chemical structure and the thermodynamic properties of non-covalent interactions would have a significant impact in many fields, ranging from catalysis to materials and medicine.^1–4^ Empirical methods have been developed based on quantitative structure activity relationships, and these tools can be applied to complex systems if experimental data is available for parameterisation.^5,6^ First principles methods based on quantum mechanics and atomistic simulation can be used to make predictions about systems for which experimental data is not available, but these methods are limited to relatively small molecular ensembles due to computational cost.^7,8^ Intermediate methods that use calculated parameters from quantum mechanics together with implementations parameterised on experimental data play a useful role in making relatively accurate predictions for relatively complex systems. One such approach is the combination of the Surface Site Interaction Point (SSIP) model that uses density functional theory (DFT) to calculate molecular descriptors *ab initio* and the Surface Site Interaction Point Model for Liquids at Equilibrium (SSIMPLE) that uses these parameters in an empirical model to calculate free energy changes associated with intermolecular interactions in solution.^9,10^ Here, we address some limitations of the SSIP model and introduce a new approach that provides a significantly more accurate description of the non-covalent interaction properties of organic molecules.

Experimentally determined free energy parameters (*α* and *β*) that describe the H-bonding properties of a wide range of different functional groups correlate well with the maximum and minimum values of the molecular electrostatic potential (MEP) calculated in the gas phase using *ab initio* methods (*E*_max_ and *E*_min_).^11^ The 0.0020 e bohr^−3^ electron density isosurface is commonly used for the calculation of molecular electrostatic potential surfaces (MEPS), because it represents an approximation of the van der Waals surface, but the MEP values calculated on this surface are anomalously perturbed by secondary electrostatic interactions with neighbouring functional groups.^12,13^ We have shown that values of *E*_max_ and *E*_min_ calculated on higher electron density isosurfaces provide a more accurate description of the effects of long range through space interactions on the H-bond donor and acceptor parameters measured for organic compounds. Specifically, the 0.0104 e bohr^−3^ electron density isosurface provides a good description of the H-bond donor parameter *α*, and the 0.0300 e bohr^−3^ electron density isosurface provides a good description of the H-bond acceptor parameter *β*. These isosurfaces lie 0.25 and 0.46 Å inside the van der Waals surface, which is consistent with interatomic distances measured in neutron diffraction studies of H-bonding interactions in the solid state.^13,14^ Here, we generalise the analysis of high electron density isosurfaces to develop a comprehensive description of the non-covalent interaction properties of organic compounds and show that the approach can be used to accurately predict partition coefficients for liquid–liquid phase transfer of a wide range of organic compounds between water and organic solvent.

## Results and discussion

### H-Bond donors

**Fig. 1:**
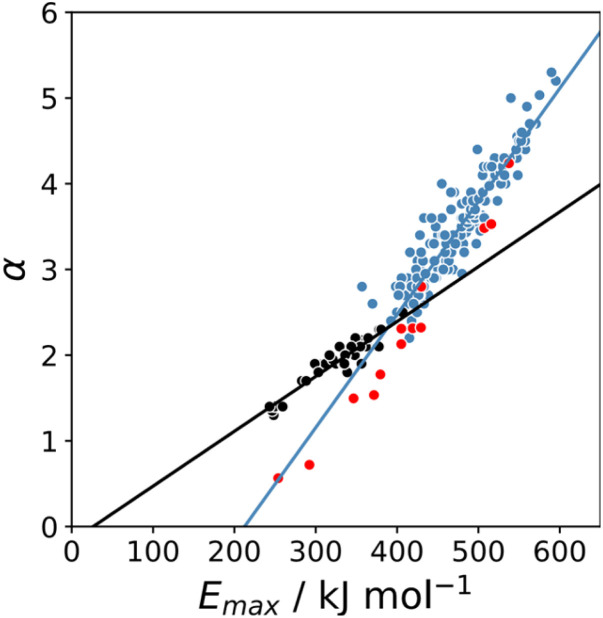
Relationship between the experimentally measured H-bond parameter *α* and the value of *E*_max_ calculated on the 0.0104 e bohr^−3^ electron density isosurface using DFT (B3LYP/6-31G* or B3LYP/6-311G** for bromine and iodine). Polar H-bond donors (OH and NH) are shown in blue, non-polar H-bond donors (CH and SH) are shown in black, and σ-holes are shown in red. The best fit straight lines are shown for polar donors and σ-holes (blue line, *m*_*α*_ = 0.0132 kJ^−1^ mol, *c*_*α*_ = −2.80, *r*^2^ = 0.89) and for non-polar donors (black line, *m*_*α*_ = 0.0072 kJ^−1^ mol, *c*_*α*_ = −0.17, *r*^2^ = 0.90).

The maximum in the MEP on the 0.0104 e bohr^−3^ electron density isosurface (*E*_max_) was calculated using density functional theory (DFT) for all compounds for which experimental *α* parameters are available.^13^Fig. 1 shows that the compounds separate into two different classes of H-bond donor, which have a different relationship between *α* and *E*_max_. Data points corresponding to polar donors (OH and NH) are shown in blue, and data points corresponding to non-polar donors (CH and SH) are shown in black. In each case, there is a linear relationship between *α* and *E*_max_ (eqn (1)). The difference between the behaviour of polar and non-polar donors presumably reflects differences in the degree of penetration of the van der Waals surface associated with H-bonding interactions at these sites.

1 *α* = *m*_*α*_*E*_max_ + c_*α*_

### σ-Holes

The other class of non-covalent interaction site that gives rise to positive values on the MEPS is the σ-hole. Interactions with σ-holes have been extensively studied in the context of halogen-bonding, and measurements of association constants (*K*) for the formation of these complexes allow determination of the corresponding *α* parameters by rearrangement of eqn (2).

2 −(*RT*/kJ mol^−1^)ln(*K*/M^−1^) = −(*α* − *α*_s_)(*β* − *β*_s_) + 6

where *β* is the H-bond acceptor parameter of the functional group that interacts with the σ-hole, and *α*_S_ and *β*_S_ are the corresponding solvent parameters.

Experimental data on halogen-bonding must be analysed with care, because there can be a significant covalent contribution in some cases. For example, the stability of the 1 : 1 complex formed by tetramethylthiourea and molecular iodine is solvent-independent, which means that the solvent competition model described by eqn (2) is not applicable. Large covalent contributions are often invoked for halogen-bonded complexes involving sulfur and nitrogen acceptors,^15–17^ so only complexes with oxygen acceptors were used to determine *α* parameters for σ-holes. A dataset of experimentally determined association constants for 67 different complexes involving 12 different σ-holes was compiled (see ESI†).^18^ For compounds that have more than one interaction site, the experimentally measured association constant includes a statistical factor related to the degeneracy of the 1 : 1 complex. For example, carbon tetrachloride has four σ-holes, one on each chlorine atom, so the experimentally measured association constant is four times the value for interaction at one site.

**Fig. 2:**
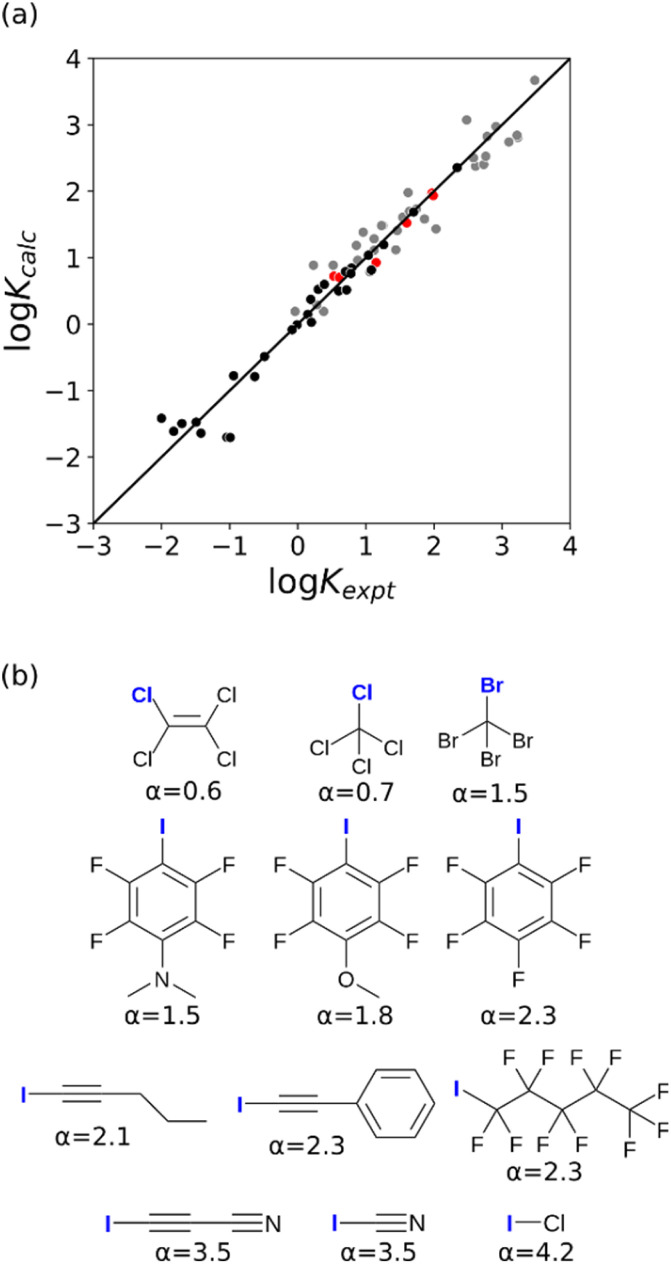
(a) Comparison of the experimentally measured values of association constant for formation of halogen-bonded complexes with oxygen acceptors (*K*_expt_) with the statistically corrected values calculated using eqn (2) (*K*_calc_). The line corresponds to *y* = *x*(RMSE = 0.32). The red data points correspond to ICN complexes and the grey data points correspond to ICl complexes. (b) The best fit *α* parameters for compounds with σ-holes on the atom highlighted in blue.

The value of *α* for each compound was optimised to obtain the best fit between the experimental values of log *K* and the statistically corrected values calculated using eqn (2). The results are shown in Fig. 2(a). The data for ICN and ICl are highlighted in red and grey respectively. For these two compounds, the data points span a wide range of log *K* values, and the fact that all points fall close to the *y* = *x* line shows that eqn (2) provides an accurate description of the thermodynamic properties of complexes formed with different acceptors in different solvents. In contrast, the experimental data for complexes formed with molecular iodine did not fit to eqn (2) (see ESI†), which suggests that even with oxygen acceptors there is a significant covalent contribution for interactions with this σ-hole.^18^ The values of *E*_max_ on the 0.0104 e bohr^−3^ electron density isosurface were calculated for each of the compounds in Fig. 2(b) using DFT, and the results are compared with the experimental *α* parameters in Fig. 1 (red data points). The properties of the σ-holes are remarkably similar to the polar H-bond donors: there is a linear correlation between *α* and *E*_max_, and the red data points fall close to the blue line, which is the best fit straight line for polar H-bond donors.

**Fig. 3:**
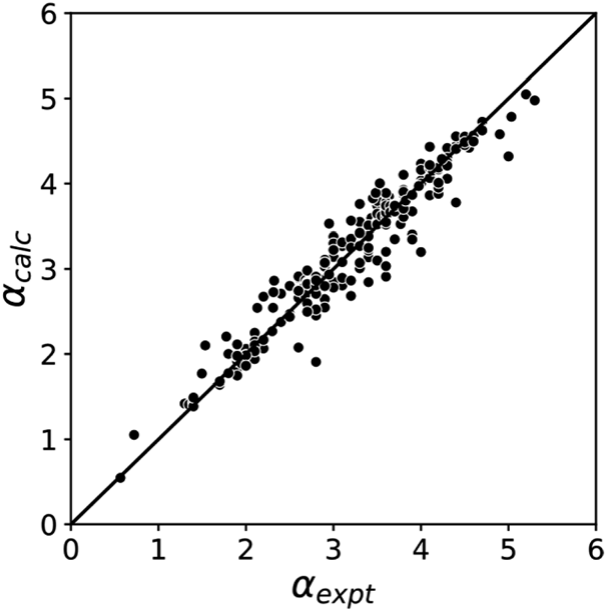
Comparison of experimental *α* parameters for H-bond donors and σ-holes (*α*_expt_) with the corresponding values calculated with eqn (1) (*α*_calc_) using *m*_*α*_ and *c*_*α*_ parameters from the lines of best fit in Fig. 1 (RMSE = 0.24).

The blue line of best fit in Fig. 1 can therefore be used to obtain values of *m*_*α*_ and *c*_*α*_ for σ-holes as well as for polar H-bond donors, and eqn (1) allows calculation of *α* parameters for σ-holes from values of *E*_max_ calculated on the 0.0104 e bohr^−3^ electron density isosurface. Fig. 3 compares the experimental values of *α* for all non-polar H-bond donors, polar H-bond donors and σ-holes with the values calculated using eqn (1). The root mean square error (RMSE) in the calculated value of *α* using this method is 0.24, which represents an improvement over the previously published method that was based on the 0.0020 e bohr^−^^3^ electron density isosurface (RMSE = 0.28 for the same dataset).^10^

### Polar H-bond acceptors

**Fig. 4:**
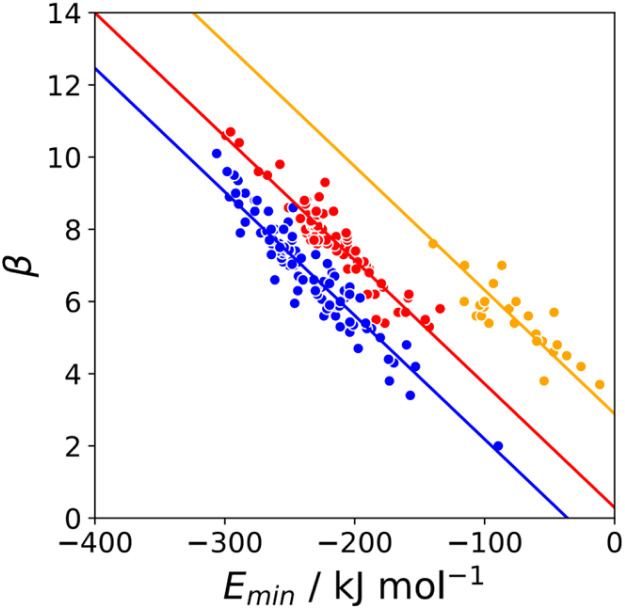
Relationship between the experimentally measured H-bond parameter *β* and the value of *E*_min_ calculated on the 0.0300 e bohr^−3^ electron density isosurface (DFT B3LYP/6-31G*) for pyridine and imine nitrogen acceptors (blue, *r*^2^ = 0.90), amide oxygen acceptors (red, *r*^2^ = 0.88), and thionyl sulfur acceptors (yellow, *r*^2^ = 0.70). The lines are the best fit to eqn (3) with *m*_*β*_ = −0.0336 kJ^−1^ mol.

The minimum in the MEP on the 0.0300 e bohr^−3^ electron density isosurface (*E*_min_) was calculated for all compounds for which experimental *β* parameters are available.^9^ A statistical correction factor was applied to obtain the *β* parameter for compounds with multiple acceptor sites (see ESI†). Fig. 4 compares the results with the experimental *β* parameters for pyridine and imine nitrogen acceptors (blue), amide oxygen acceptors (red), and thionyl sulfur acceptors (yellow). The H-bond acceptor properties of each of these functional groups is well-described by eqn (3), and the slope of the best fit straight line is practically identical for different types of acceptor.

3 *β* = *m*_*β*_*E*_min_ + *c*_*β*_

Similar results were obtained for all the functional groups listed in Table 1: the intercept depends on atom type, but the slopes are similar (see ESI†). The values of the intercepts (*c*_*β*_) were therefore separately optimised for each functional group, and the slopes (*m*_*β*_) were constrained to be identical to minimise the number of variables using in fitting the data to eqn (3). The resulting value of *m*_*β*_ was −0.0336 kJ^−1^ mol, and the *c*_*β*_ parameters are listed in Table 1. The values of *c*_*β*_ span a wide range, from −3 to +5, which reflects differences in the underlying processes that determine the free energy changes associated with H-bond formation for different functional groups. Platts has previously compared empirical H-bond acceptor parameters with molecular properties calculated *ab initio* in the gas phase, and correlations were only obtained if the H-bond acceptors were split according to atom type.^19^ In contrast when the calculated properties of the corresponding hydrogen fluoride complexes were used, correlations were found without considering atom type. This result suggests that there are properties of the bound state that are not captured by considering the H-bond acceptor in the free state. A possible interpretation of the *c*_*β*_ parameter is therefore that it provides an empirical correction that quantifies the difference in polarity between the bound and free states, which varies for different types of H-bond acceptor. For example, Table 1 shows that amide acceptors have a larger value of *c*_*β*_ than ketones. Polarisation of the amide bond due to resonance assistance from the nitrogen lone pair could increase the polarity of the oxygen acceptor in the bound state by a mechanism that is not available to ketones, which may account for the large difference in the value of *c*_*β*_.^20^

**Table 1:** *c*_*β*_ parameters for polar H-bond acceptors

Functional group	*c*_*β*_
Nitrile	−0.64
Pyridine, imine	−1.08
Aniline	−2.57
Primary amine	−2.94
Secondary amine	−1.63
Tertiary amine	−0.98
Ketone, aldehyde, ester, carboxylic acid	−0.42
Amide, urea, carbamate, amidate	0.44
*N*-oxide	1.51
Nitro	−1.33
Phosphine oxide	2.03
Sulfoxide, sulfone	0.44
Alcohol	−1.57
Phenol, ether, epoxide	−0.66
Fluoro	0.31
Phosphine sulfide	4.52
Thionyl	2.94

**Fig. 5:**
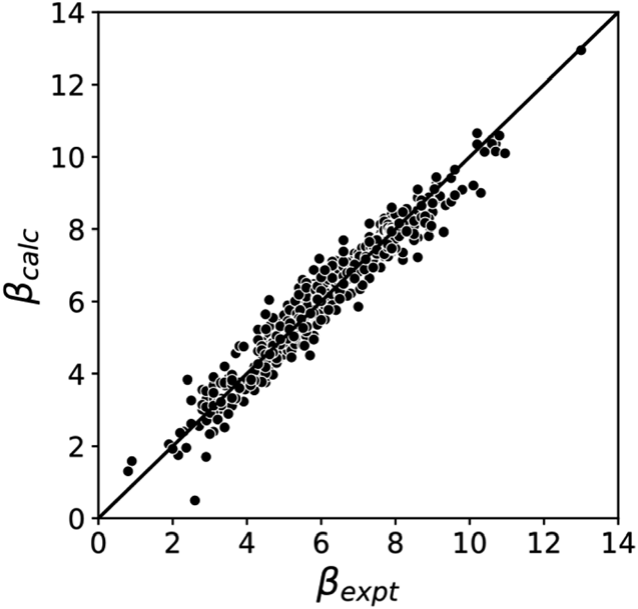
Comparison of experimental *β* parameters for polar H-bond acceptors (*β*_expt_) with the corresponding values calculated with eqn (3) (*β*_calc_) using *m*_*β*_ = −0.0336 kJ^−1^ mol and *c*_*β*_ parameters from Table 1 (RMSE = 0.41).

Fig. 5 compares the H-bond acceptor parameters calculated using the parameters in Table 1 in eqn (3) with the corresponding experimental values. The RMSE is 0.41, which represents a significant improvement over the previously published method that was based on the 0.0020 e bohr^−3^ electron density isosurface (RMSE = 0.70 for the same dataset).^10^

### Non-polar H-bond acceptors

**Fig. 6:**
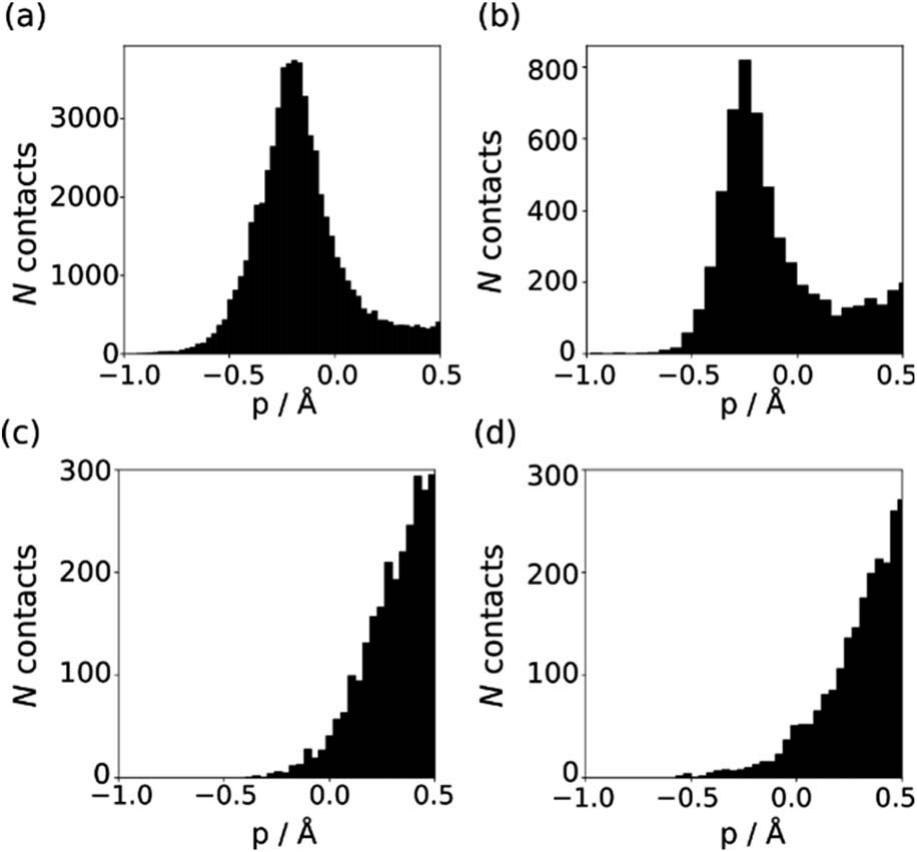
Distribution of *p* values in the CSD for H-bond interactions of NH and OH H-bond donors with (a) carbonyl oxygen (b) thionyl sulfur (c) chlorine in chloroalkanes (d) thioether sulfur.

The behaviour of less polar H-bond acceptors is quite different. For hydrocarbon π-systems, chlorine, bromine or iodine acceptors, comparison of the experimental values of *β* with the values of *E*_min_ calculated on the 0.0300 e bohr^−3^ electron density isosurface results in poor agreement with eqn (3) and different values of *m*_*β*_. Analysis of H-bond lengths in X-ray crystal structures in the Cambridge Structure Database (CSD) indicates that there are fundamental differences between the interactions with polar and non-polar H-bond acceptors.^21,22^Fig. 6 illustrates the degree of penetration for H-bonding interactions between NH and OH donors with different types of acceptor (*p* is defined as the difference between the sum of the van der Waals radii and the interatomic distance in the crystal structure). Fig. 6(a) shows data for H-bonding interactions with a polar H-bond acceptor, carbonyl oxygen. The peak in the distribution of *p* values is well inside the sum of the van der Waals radii.^23^ In contrast, there is no penetration of the van der Waals surface when H-bond donors interact with chlorine (Fig. 6(c)). This result confirms that the 0.0300 e bohr^−3^ electron density isosurface is unlikely to provide a good description of the H-bonding properties of non-polar acceptors and suggests that an isosurface closer to the van der Waals surface should be used.

The *p* distribution provides a useful tool to distinguish polar and non-polar H-bond acceptors. For example, Fig. 6 shows the *p* distributions for two different types of sulfur acceptor. For thionyl sulfur (Fig. 6(b)), the *p* distribution resembles the carbonyl oxygen distribution in Fig. 6(a) with significant penetration of the van der Waals surface, so this functional group is considered a polar H-bond acceptor.^24^ In contrast, the *p* distribution for thioether sulfur (Fig. 6(d)) resembles the chlorine distribution in Fig. 6(c) with no penetration of the van der Waals surface, so this functional group is considered a non-polar acceptor.^25^

**Fig. 7:**
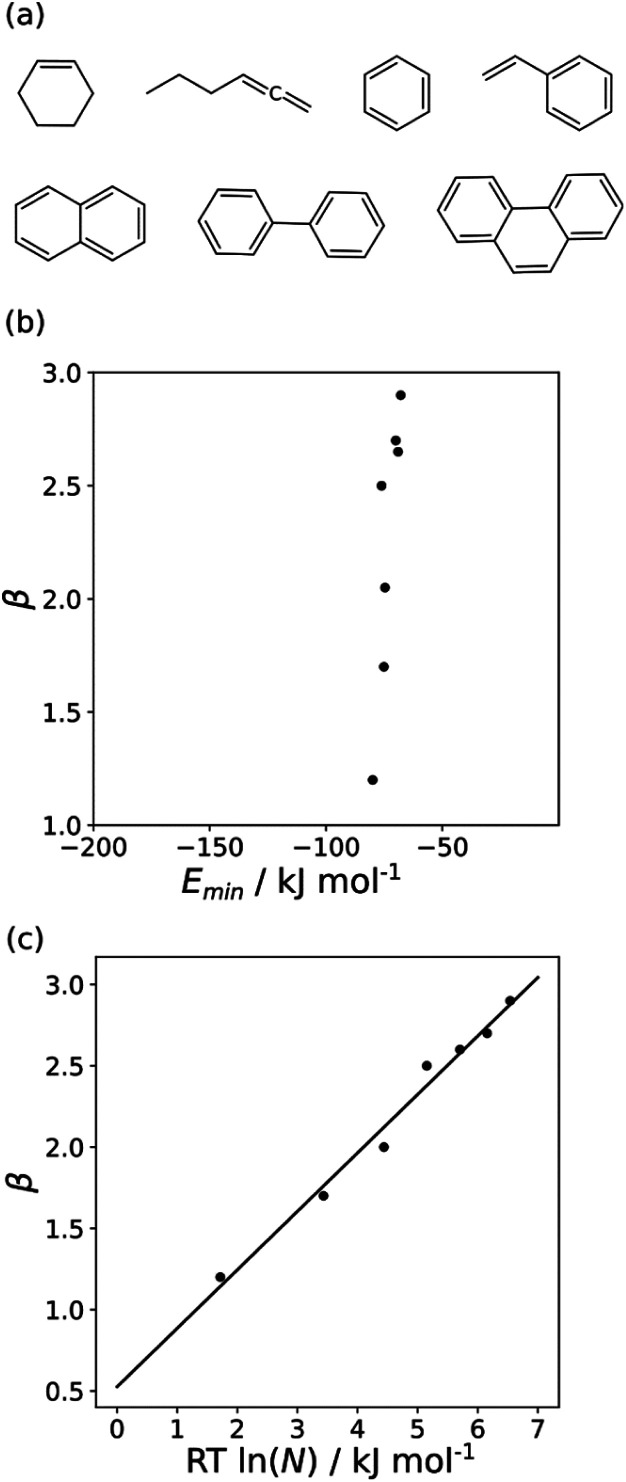
(a) Structures of hydrocarbon π-systems with different numbers of carbon atoms in the π-system (*N*). (b) Comparison of the experimentally measured H-bond parameter *β* for these compounds with the value of *E*_min_ calculated on the 0.0020 e bohr^−3^ electron density isosurface (DFT B3LYP/6-31G*). (c) Comparison of *β* with *N*. The line is the best linear fit (*β* = 0.36 *RT* ln *N* + 0.53, *r*^2^ = 0.98).

The minimum in the MEP on the 0.0020 e bohr^−3^ electron density isosurface (*E*_min_) was calculated for all non-polar acceptors for which experimental *β* parameters are available.^10^Fig. 7(b) compares the results with the experimental *β* parameters for the series of hydrocarbon π-systems shown in Fig. 7(a). The values of *E*_min_ are practically identical for all of these compounds, but there are large variations in the experimentally determined *β* parameters. The variation in *β* parameters is related to the size of the π-system (Fig. 7(c)). This result suggests that there is an entropic contribution to the experimentally determined values of *β*, which is related to the degeneracy associated with multiple sites of interaction.^26^ An approach that accounts for differences in contact surface area is therefore required to understand the properties of non-polar H-bond acceptors.

**Fig. 8:**
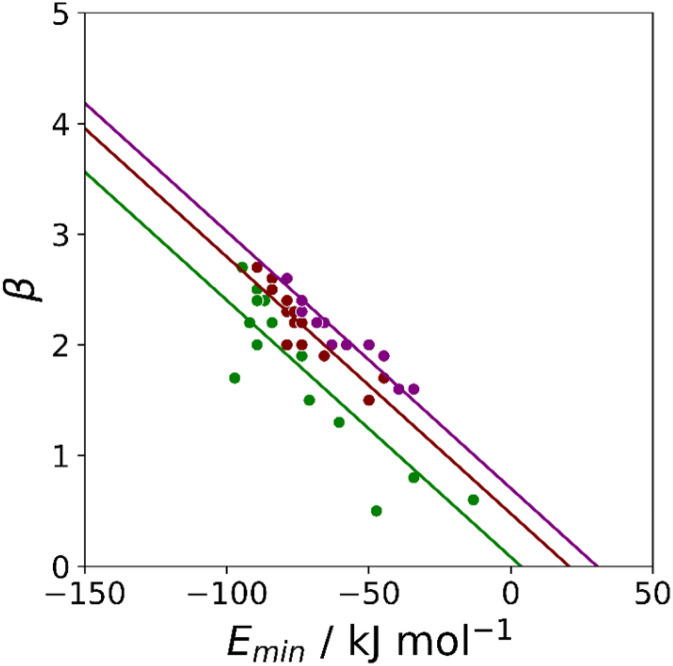
Relationship between the experimentally measured H-bond parameter *β* and the value of *E*_min_ calculated on the 0.0020 e bohr^−3^ electron density isosurface using DFT, B3LYP/6-31G* for chlorine acceptors (green, *r*^2^ = 0.79) and B3LYP/6-311G** for bromine (maroon, *r*^2^ = 0.82) iodine (purple, *r*^2^ = 0.91). The lines are the best fit constraining the slope to be constant (*m*_*β*_ = −0.0232 kJ^−1^ mol).

Haloalkanes that contain only chlorine, bromine or iodine provide three datasets of non-polar acceptors with fixed contact surface areas that can be used to compare experimental *β* parameters with calculated MEP values. Although there is some scatter, the values of *E*_min_ calculated on the 0.0020 e bohr^−3^ electron density isosurface correlate well with the experimental *β* parameters, and all three datasets can be fit to eqn (3) with the same slope (Fig. 8). The value of the intercept increases with the size of the halogen, which is consistent the surface area correlation observed for hydrocarbon π-systems. Fig. 8 suggests that the 0.0020 e bohr^−3^ electron density isosurface can provide a good description of the H-bonding properties of non-polar acceptors using a fixed value of *m*_*β*_, but additional parameterisation will be required to determine a method for calculating an appropriate value of *c*_*β*_ for different functional groups (see discussion of solvation free energies below).

### Atomic surface site interaction points

**Fig. 9:**
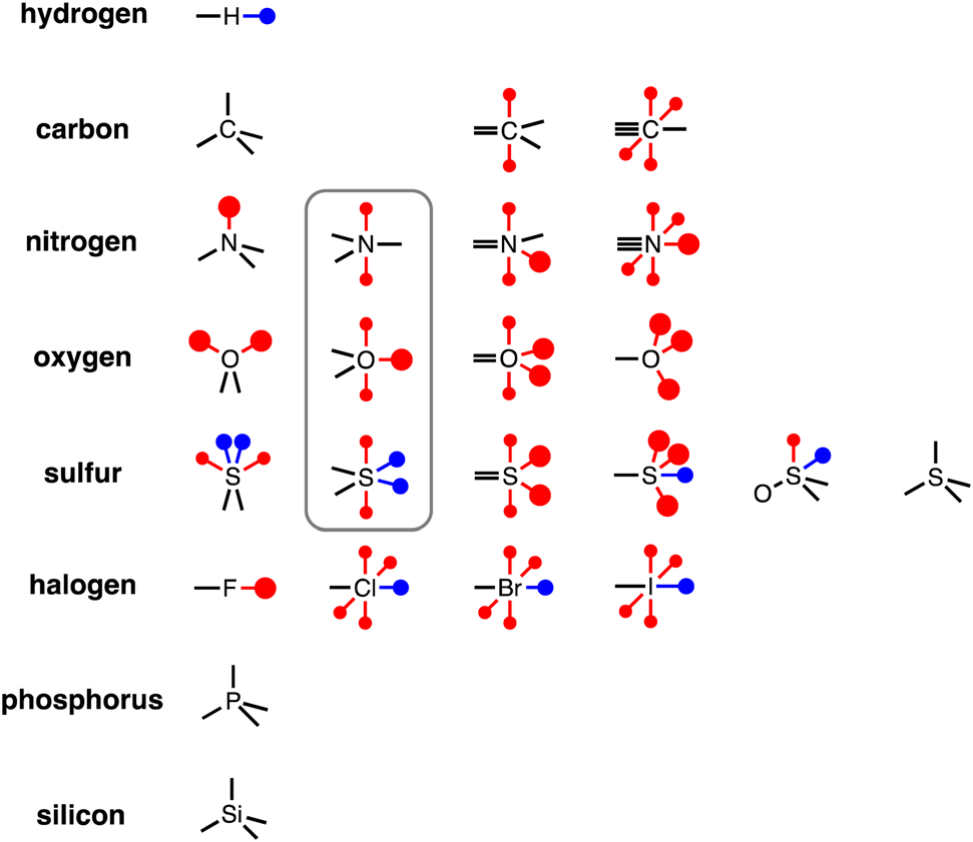
AIP representation of different functional groups (the grey box highlights atoms that have the same valency as the atom to the left but have a π-system). The large red dots represent polar H-bond acceptor sites (lone pairs) assigned using the 0.0300 e bohr^−3^ electron density isosurface, the small red dots represent non-polar H-bond acceptor sites (*e.g.* π-systems) assigned using the 0.0020 e bohr^−3^ electron density isosurface, and the blue dots represent H-bond donors and σ-holes assigned using the 0.0104 e bohr^−3^ electron density isosurface.

To generalise this methodology for calculating non-covalent interaction parameters to the entire surface of a molecule, we use the three different electron density isosurfaces discussed above to assign a set of Atomic Surface Site Interaction Points (AIP) to each atom in a molecule. Fig. 9 illustrates the AIP description for a variety of different atom types.^27^ Lone pairs on oxygen, nitrogen and sp^2^ sulfur atoms were readily identified as local minima in the MEP on the 0.0300 e bohr^−3^ electron density isosurface (large red AIPs in Fig. 9).^13^ Fluorine generally has less well-defined minima on this MEPS and is represented by a single AIP located on the end of the C–F bond (Fig. 9). H-bond donor sites on hydrogen atoms and σ-holes on chlorine, bromine, iodine and sulfur were identified as local maxima in the MEP on the 0.0104 e bohr^−3^ electron density isosurface (blue AIPs in Fig. 9). The 0.0020 e bohr^−3^ electron density isosurface was used to assign most of the other AIPs (small red AIPs in Fig. 9), but the location and number of these AIPs are not readily identified by local maxima and minima on the MEPS.

The SSIP model that we developed previously was based on the assumption that the surface area of a water molecule can be used to determine the footprint of a H-bonding interaction, *i.e.* 9.35 Å^2^ on the 0.0020 e bohr^−3^ electron density isosurface.^10,28^ Since the non-polar van der Waals contribution to non-covalent interactions is proportional to contact surface area, this assumption allows the van der Waals energy associated with an intermolecular SSIP contact to be treated as a constant (*E*_vdW_ = −5.6 kJ mol^−1^).^29^ This approach was therefore adopted for the new atomic version of the SSIP model described here, so that AIPs can be implemented directly in the Surface Site Interaction Point Model for Liquids at Equilibrium (SSIMPLE) to calculate solvation free energies. An analysis of atomic surface areas was therefore used to determine the correct number of non-polar AIPs that should be used to represent non-polar interaction sites.

For each of the compounds in the database of experimental α and *β* parameters discussed above, the surface area associated with each atom was calculated on the 0.002 e bohr^−3^ electron density isosurface. For each atom type in each functional group, the average value of the atomic surface area was calculated. Each oxygen, nitrogen or sulfur lone pair and each chlorine, bromine or iodine σ-hole was assigned a surface area of 9.35 Å^2^, and the remaining atomic surface area was assumed to represent the less polar interaction sites highlighted in Fig. 9. The average surface area available for each type of non-polar AIP was calculated, and the results are summarised in Table 2. For most non-polar AIPs, the area is significantly less than 9.35 Å^2^, so we introduce a scaling factor (*f* = average surface area/9.35) to account for the smaller footprint associated with these sites. Table 2 shows that the scaling factors for non-polar AIPs can conveniently be rounded to the nearest quarter. For sp^2^ oxygen, the total surface area is equivalent to two AIPs, so there is no surface area available for non-polar interactions, *i.e. f* = 0 for oxygen π-systems, and these sites are not used to describe carbonyl or nitro groups. Similarly, the surface area associated with oxygen atoms in P-oxides and N-oxides corresponds to two AIPs, so the third lone pair site shown in Fig. 9 is not used.

**Table 2:** Average surface areas associated with non-polar AIP sites and corresponding scaling factors (*f*)

AIP type	Average surface area/Å^2^	*f*
Carbon π-system	4.45	0.50
Nitrogen π-system	2.65	0.25
Oxygen π-system	−0.10	0.00
Chlorine p-orbital	5.10	0.50
Bromine p-orbital	6.78	0.75
Iodine p-orbital	9.15	1.00
Sulfur π-system or σ-hole	4.37	0.50

The interaction parameters (α or *β*) used to describe non-polar AIPs were generally obtained from the maximum or minimum in the MEP on the patch of surface associated with the corresponding atom. For some atom types, the atomic surface naturally separates into the correct number of patches, *e.g.* for sp^2^ carbon there are two well-defined patches, one above and one below the π-face. For other atom types, there are continuous areas of surface that describe more than one interaction site. For example in chlorine, the four p-orbital sites all lie on the same cylindrical surface. In this case, the minimum in the MEP was assigned as the first p-orbital site, and the other three sites were arranged on the surface to achieve the maximum separation in space. For atom types that have both lone pair and π-sites on a single continuous surface, a different strategy is required to separate the AIPs. For example, sp^2^ nitrogen has two π-sites and one lone pair on the same patch of surface. In this case, the minimum in the MEP on the 0.0300 e bohr^−3^ electron density isosurface was used to locate the lone pair site, and then a radius of 1.24 Å was used to define the footprint of an interaction at this point. All points on the 0.0020 e bohr^−3^ electron density isosurface that fell within this footprint were removed, and the maxima in the MEP on the two remaining patches were used to assign *β* parameters to the π-sites. Full details of the precise procedure for each atom type are provided in the ESI.†

### Solvation free energies

We have previously shown that solvation free energies can be calculated by treating the liquid state as an ensemble of interacting SSIPs at equilibrium.^30,31^ The association constant for formation of an interaction between SSIP *i* and SSIP *j*, *K*_*ij*_, is given by eqn (4).4
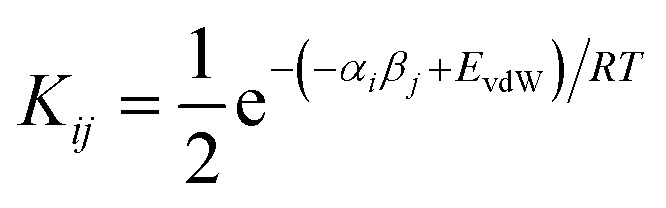


Given the total concentration of each SSIP present in the liquid ([total]_*i*_), it is possible to use eqn (4) to calculate the fraction of each SSIP that is not bound to another SSIP ([free]_*i*_). For each SSIP, the concentration of free SSIPs defines the solvation free energy in solvent S, Δ*G*_S_(*i*), according to eqn (5).5
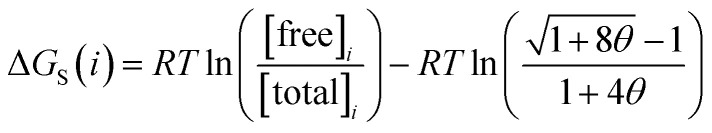
where the second term accounts for any differences in the total density of SSIPs (*θ*) present in different liquid phases.

The molecular solvation energy in solvent S, Δ*G*_S_, can be obtained simply by summing the solvation energies of each SSIP used to represent that molecule. Precisely the same formulation can be applied to AIPs, except that the solvation free energy for each AIP must be multiplied by the corresponding scaling factor, *f*_*i*_ (eqn (6)), and the total concentration of each AIP that is used to determine the speciation of contacts must be scaled in the same way.6
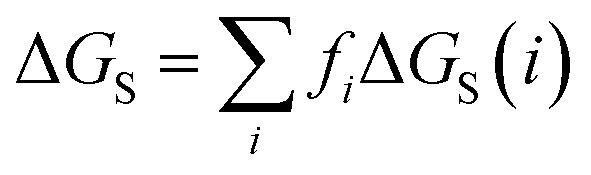


**Fig. 10:**
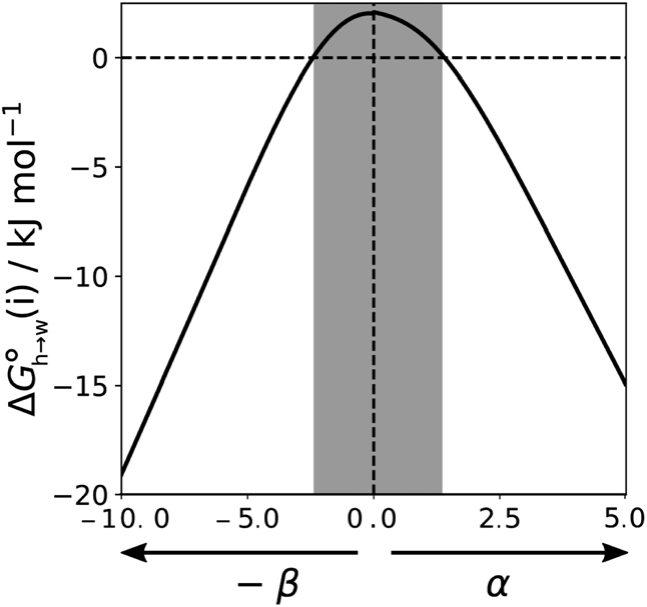
Partition profile showing the free energy of transfer of a single AIP from *n*-hexadecane into water calculated using SSIMPLE 
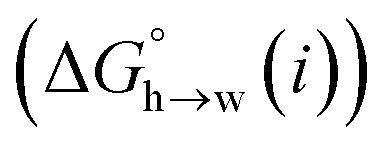
. Water is described by an AIP on each hydrogen (*α* = 2.80) and an AIP on each lone pair (*β* = 4.50), and *n*-hexadecane is described by an AIP on each hydrogen (*α* = 0.47). The shading highlights hydrophobic solute AIPs.

Eqn (4)–(6) can be used to calculate the free energy of transfer of a compound between two different liquid phases. Conversely, the experimentally measured free energies of transfer can be used to parameterise the AIP model.^32^ As we have shown previously, experimental data on the free energies of transfer between *n*-hexadecane and water 
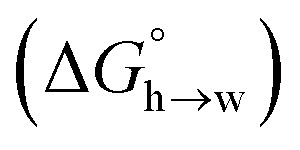
 is particularly useful for parameterisation of non-polar interaction sites.^27^Fig. 10 shows an example of a partition profile calculated using SSIMPLE, which gives the free energy change for transfer of a single solute AIP from *n*-hexadecane to water as a function of the AIP interaction parameter *α* or *β* (eqn (7)).7
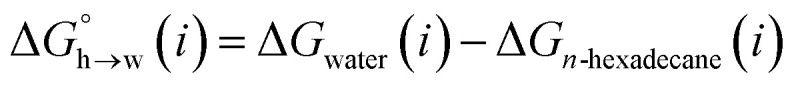


**Fig. 11:**
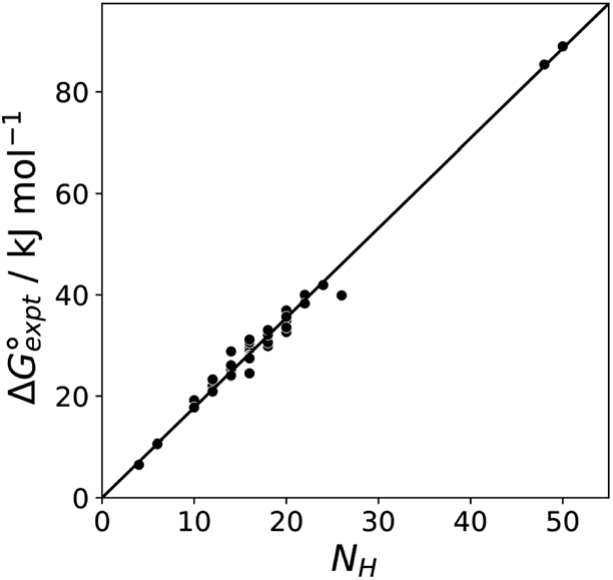
Experimental free energy of transfer of alkanes from *n*-hexadecane to water 
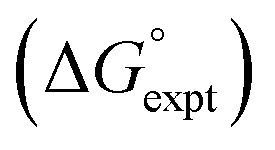
 plotted as a function of the number of hydrogen atoms (*N*_H_).

**Fig. 12:**
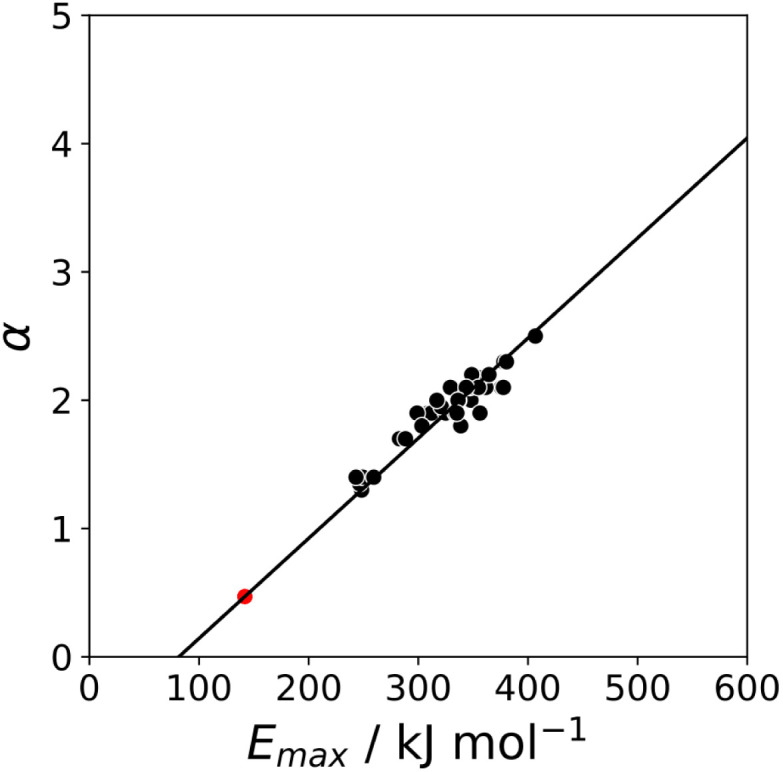
Relationship between the experimentally measured H-bond parameter *α* for non-polar donors and the value of *E*_max_ calculated on the 0.0104 e bohr^−3^ electron density isosurface using DFT (B3LYP/6-31G* or B3LYP/6-311G** for bromine and iodine). The best fit straight line was constrained to pass through the alkane CH point shown in red (*m*_*α*_ = 0.0078 kJ^−1^ mol, *c*_*α*_ = −0.64, *r*^2^ = 0.93).

Fig. 11 shows the relationship between the experimental free energy of transfer from *n*-hexadecane to water for alkanes and the number of hydrogen atoms (*N*_H_).^27^ The linear correlation justifies employing one AIP for each CH hydrogen and shows that AIPs can be used directly to calculate the free energy of transfer from *n*-hexadecane to water relative to the 1 M standard state, and the fact that the line of best fit passes through the origin means that an additional constant is not required.^27^ The slope in Fig. 11 represents the contribution to the overall free energy of transfer of one CH AIP (+1.8 kJ mol^−1^). An identical result for the free energy of transfer of a hydrocarbon CH group is obtained by considering homologous series of different functional groups (see ESI†). Alkanes only contain the positive AIPs associated with CH groups, so when an alkane is dissolved in *n*-hexadecane there are no polar interactions between AIPs. As a result, the value of *K*_*ij*_ for all AIP interactions is a constant determined only by the van der Waals term in eqn (4). The solvation energy of an alkane in *n*-hexadecane is therefore independent of the value of *α* used to describe an alkane CH group. Thus the value of *α* that best represents an alkane CH can be read off Fig. 10 by looking at 
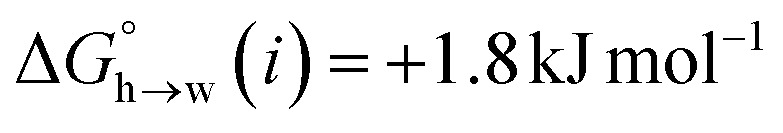
, which gives *α* = 0.47. This parameter provides a reliable anchor point for parameterisation of eqn (1) for non-polar donors, and Fig. 12 shows that there is good agreement with the experimentally measured *α* parameters for all CH and SH donors. The best fit straight line in Fig. 12 was constrained to pass through the alkane CH point (red) to give *m*_*α*_ = 0.0078 kJ^−1^ mol and *c*_*α*_ = −0.64 for non-polar H-bond donors.

A similar approach was used to parameterise eqn (3) for non-polar H-bond acceptors, based on the experimental free energy of transfer of unsaturated hydrocarbons from *n*-hexadecane to water. For each compound, the values of *α* for the CH AIPs were calculated from the maxima in the 0.0104 e bohr^−3^ electron density isosurface using eqn (1), and the corresponding contributions to the overall free energy of transfer were obtained from SSIMPLE. The experimental free energy of transfer from *n*-hexadecane to water is given by the sum of the contributions due to the CH AIPs and the AIPs that represent the π-sites (eqn (8)).8
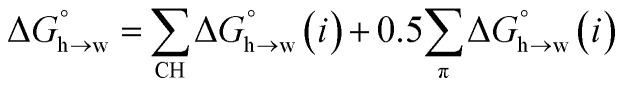
where 0.5 is the scaling factor *f* used for carbon π-system AIPs.

**Fig. 13:**
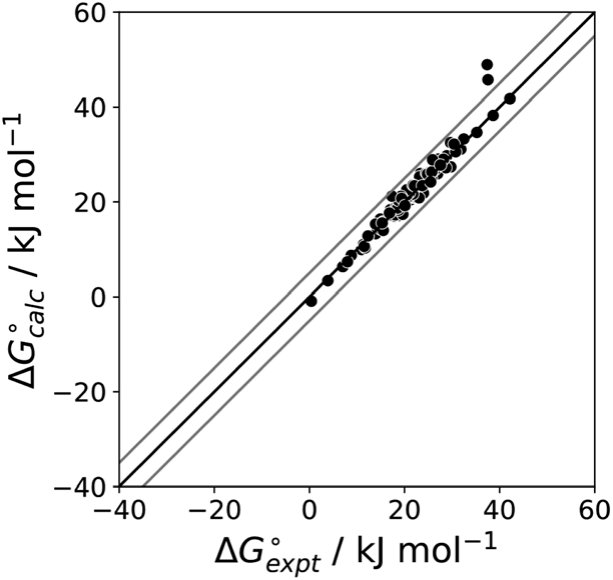
Comparison of the experimental free energy of transfer of 108 unsaturated hydrocarbons from *n*-hexadecane to water 
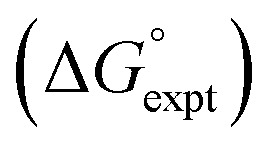
 with the value calculated using the AIP model in SSIMPLE 
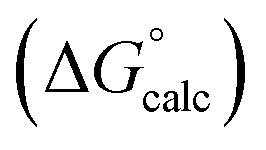
. The black line is *y* = *x*, and the grey lines indicate deviations of ±5 kJ mol^−1^ (RMSE = 1.8 kJ mol^−1^).

The MEP analysis in Fig. 7(b) suggests that hydrocarbon π-system AIPs have similar values of *β*, which means that 
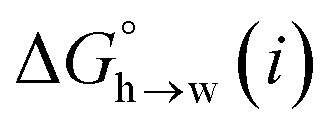
 for the π-sites can be treated as a constant. Using the experimental data for 108 unsaturated hydrocarbons with the calculated values of 
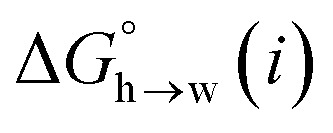
 for the CH AIPs in eqn (8) gives an average value of +1.0 kJ mol^−1^ for the free energy of transfer of one hydrocarbon π-system AIP from *n*-hexadecane to water. The value of *β* that best represents a hydrocarbon π-system can therefore be read off Fig. 10 by looking at 
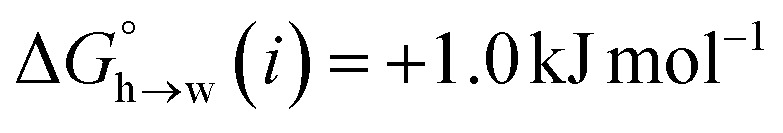
, which gives *β* = 2.0. This parameter provides an anchor point for parameterisation of eqn (3) for non-polar acceptors using the minimum in the MEP calculated on the 0.0020 e bohr^−3^ electron density isosurface. Using this *β* value with the value *m*_*β*_ obtained for halogen acceptors (−0.0232 kJ^−1^ mol) gives *c*_*β*_ = 0.0, and Fig. 13 shows that these parameters provide an excellent description of the phase transfer properties of unsaturated hydrocarbons.

**Fig. 14:**
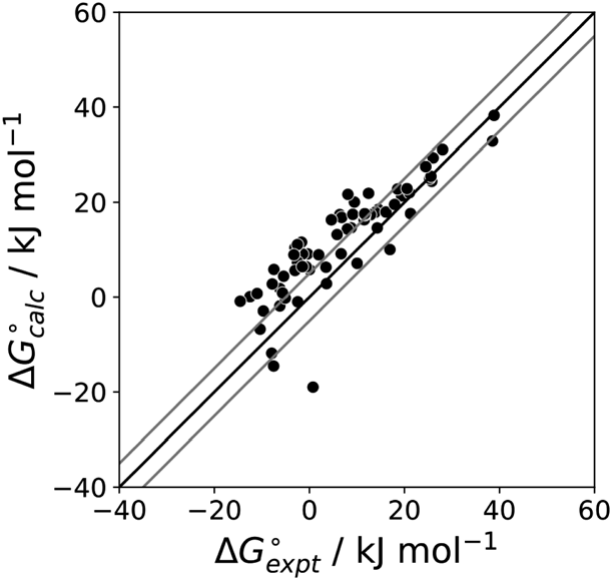
Comparison of the experimental free energy of transfer of 65 compounds with positive π-systems from *n*-hexadecane to water 
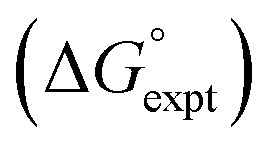
 with the value calculated using the AIP model in SSIMPLE 
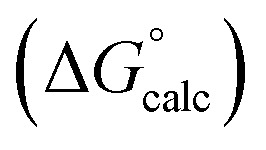
 (*m*_*α*_ = 0.0206 kJ^−1^ mol, *c*_*α*_ = 0.00, RMSE = 7.9 kJ mol^−1^). The black line is *y* = *x*, and the grey lines indicate deviations of ±5 kJ mol^−1^.

For some π-systems with very electron-withdrawing substituents, the MEP can be positive across the entire surface of the atom, so a different approach is required to calculate the value of *α* required to describe these atoms. The intercept of zero (*c*_*β*_) for negative π-systems suggests that a similar approach could be used to obtain *α* parameters for positive π-systems, *i.e.* parameterisation of eqn (1) using the maximum in the MEP calculated on the 0.0020 e bohr^−3^ electron density isosurface and a value of zero for *c*_*α*_. A set of compounds were selected for which experimental values of the free energy of transfer from *n*-hexadecane to water are available and which have carbon π-systems where the minimum in the MEP is positive (see ESI†). The interaction parameters for the positive π-sites were obtained using eqn (1) with *c*_*α*_ = 0.0, and the value of *m*_*α*_ was optimised to obtain the lowest RMSE between the calculated and experimental values of the transfer free energies. The agreement between calculation and experiment shown in Fig. 14 is not as good as that found for negative π-systems in Fig. 13, because the compounds used for positive π-systems contain a wide range of different functional groups, which complicates the analysis. However, this approach does provide a useful method for estimating an appropriate value of *m*_*α*_.

**Fig. 15:**
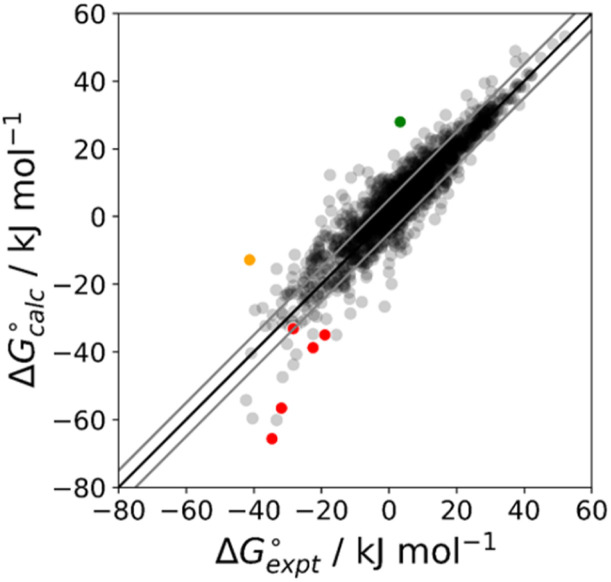
Comparison of the experimental free energy of transfer of 1504 compounds from *n*-hexadecane to water 
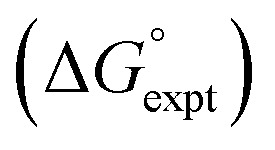
 with the value calculated using the AIP model in SSIMPLE 
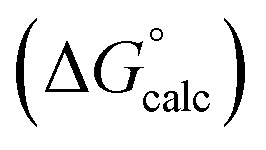
 (RMSE = 5.2 kJ mol^−1^). The black line is *y* = *x*, and the grey lines indicate deviations of ±5 kJ mol^−1^. Outliers are highlighted in colour.

The final AIP model was tested using experimental data on the free energy of transfer of 1504 compounds from *n*-hexadecane to water. This dataset includes the compounds used to parameterise the model as described above, as well as a large number of additional compounds that contain multiple different functional groups. Fig. 15 shows the results. The agreement between calculation and experiment is generally good (RMSE = 5.2 kJ mol^−1^) with a small number of outliers. The green point is diphenyl ether: the experimental value (3 kJ mol^−1^) is much lower than the value for methyl phenyl ether (12 kJ mol^−1^) even though diphenyl ether has a larger hydrophobic surface, which suggests that the experimental value for diphenyl ether is incorrect. The yellow point is tryptophan: the calculated value is incorrect, because the calculations were carried out assuming all functional groups are in the neutral form but amino acids are zwitterionic in water.^33^ The red points correspond to nucleobases and guanine compounds and are predicted to be more polar than the experiment suggests. Visual inspection of the AIP description of these molecules shows no obvious discrepancies, but the molecules all contain densely packed arrays of polar functional groups. The SSIMPLE approach assumes that all AIPs are independent of one another, so the error in these cases may be related to sub-optimal solvation of neighbouring sites in water.

### AIP interaction maps of non-covalent complexes

**Fig. 16:**
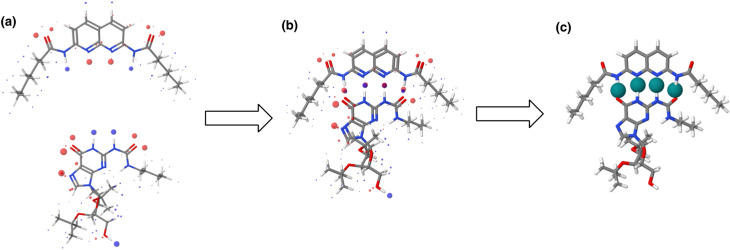
(a) The three-dimensional structures of two molecules that form a quadruply H-bonded complex were extracted from the X-ray crystal structure of the complex and footprinted to obtain the AIP descriptions shown (blue indicates a H-bond donor or positive site, red indicates a H-bond acceptor or negative site, and the diameters of the balls are proportional to the corresponding AIP *α* and *β* parameters). (b) The AIP descriptions of the molecular components superimposed on the X-ray crystal structure of the complex.^34^ (c) Favourable intermolecular interactions in the X-ray crystal structure are shown as green balls. Interactions were identified as close contacts between AIPs on different molecules (<1.7 Å separation), and only contacts that make a significant contribution to the free energy of interaction in chloroform solution are shown (ΔΔ*G* < −4 kJ mol^−1^).

The AIP analysis can also be applied to non-covalent complexes that make multiple intermolecular interactions. Fig. 16(a) shows the AIP description of two compounds that make a quadruply H-bonded complex. The three-dimensional structure of this complex has been characterised by X-ray crystallography,^34^ and Fig. 16(b) shows the AIPs mapped on the three-dimensional structure. The four intermolecular H-bonding interactions can be identified as pairs of red AIPs (H-bond acceptor sites) and blue AIPs (H-bond donor sites) that coincide in space. For each of these contacts, the free energy contribution to the overall intermolecular binding energy in the solid state can be calculated simply as the product of the corresponding *α* and *β* parameters (ΔΔ*G* in kJ mol^−1^). Moreover by including solvation free energies calculated using SSIMPLE, this approach can be extended to obtain the free energy contribution of each interaction to the overall binding energy in solution (eqn (9), see ref. 31 for the derivation of this relationship).9
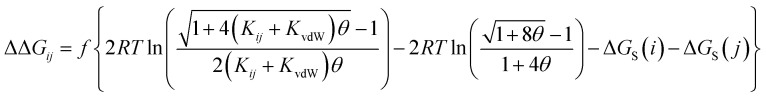
where ΔΔ*G*_*ij*_ is the free energy contribution due the intermolecular interaction between AIP *i* and AIP *j*, *K*_*ij*_ is given by eqn (4), *K*_vdW_ is the corresponding equilibrium constant for a non-polar interaction (from eqn (4) with *α*_*i*_*β*_*j*_ = 0), Δ*G*_S_(*i*) is the solvation energy of AIP *i* calculated using eqn (5), *f* is the lower of the two AIP scaling factors, and *θ* is the total density of SSIPs in the solution.

This example illustrates how AIPs can be used to convert a three-dimensional structure into a map of the non-covalent interactions that are present in an intermolecular complex. The AIP description can be used not only to identify and visualise non-covalent interactions, but it also provides a quantitative estimate of the free energy contribution associated with each intermolecular contact. Fig. 16(c) shows a visualisation of the non-covalent interactions present in the quadruply H-bonded complex in chloroform solution. Only contacts that contribute appreciably to the free energy of intermolecular interaction are displayed (|ΔΔ*G*| > 0.35 kJ mol^−1^). Although there are additional contacts between the alkyl side chains in the X-ray crystal structure, they are associated with negligible free energy contributions, and it is the four H-bonds that provide the driving force for complexation in chloroform solution.

**Fig. 17:**
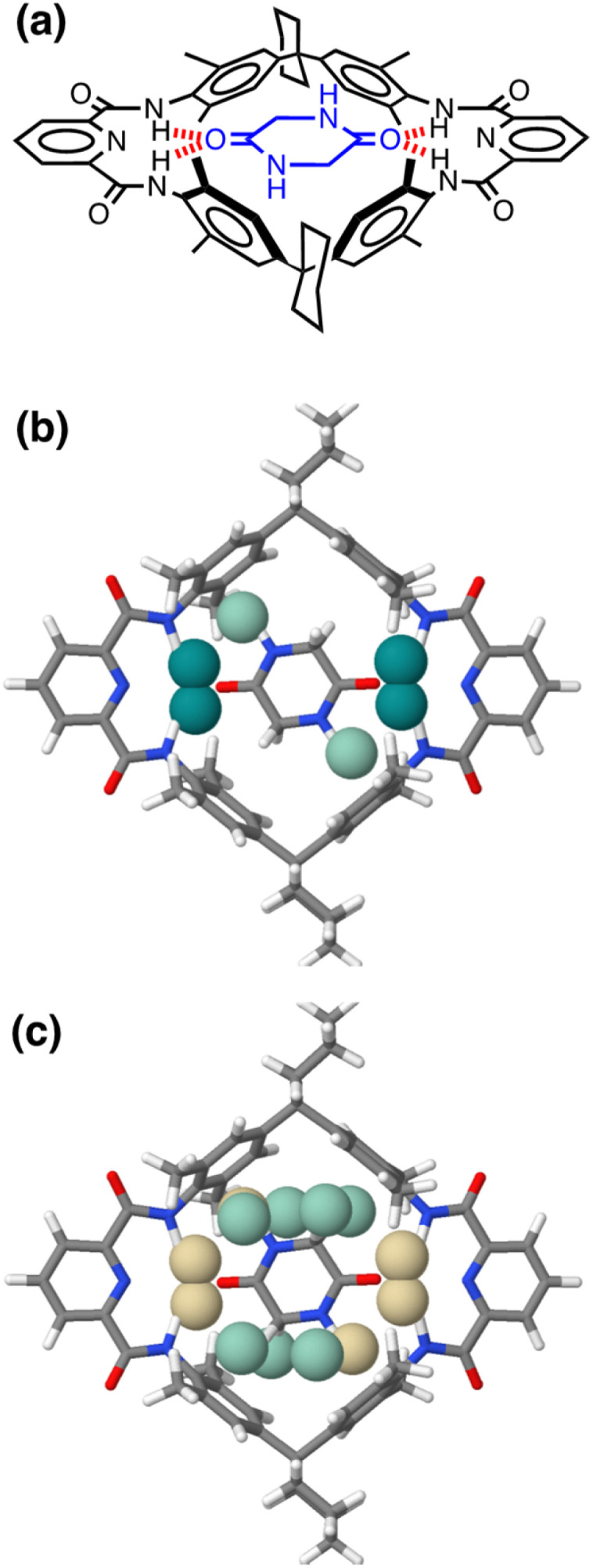
(a) Chemical structure of a host–guest complex.^35^ (b) AIP map of intermolecular interactions that make a significant contribution to the free energy of interaction in chloroform solution (|ΔΔ*G*| > 0.35 kJ mol^−1^): strong favourable interactions shown as dark green balls (ΔΔ*G* < −4 kJ mol^−1^), and weak favourable interactions as pale green balls (ΔΔ*G* > −4 kJ mol^−1^). (c) AIP map of intermolecular interactions that make a significant contribution to the free energy of interaction in water (|ΔΔ*G*| > 0.35 kJ mol^−1^): unfavourable interactions are shown as yellow balls, and weak favourable interactions as pale green balls (ΔΔ*G* > −4 kJ mol^−1^).

Fig. 17 shows the AIP analysis applied to a host–guest complex in two different solvent environments. Mapping the AIPs onto the X-ray crystal structure of the complex reveals a large number of close contacts between the two molecules.^35^Fig. 17(b) shows the AIP interaction map in chloroform, where binding is dominated by H-bonding interactions between the amide groups (ΔΔ*G* ≈ −5 kJ mol^−1^ per contact) and NH–π interactions with the side-walls of the binding site (ΔΔ*G* ≈ −0.5 kJ mol^−1^ per contact). Fig. 17(c) shows the corresponding interaction map for the same complex in aqueous solution. In this case, all of the polar interactions are unfavourable (yellow), due to the large desolvation penalty associated with breaking H-bonding interactions between the amide groups and water molecules (ΔΔ*G* ≈ +1 kJ mol^−1^ per contact). However, these unfavourable contributions are compensated by a large number of favourable interactions (green) associated with hydrophobic contacts between non-polar sites on the guest with the non-polar side-walls of the cavity (ΔΔ*G* ≈ −3 kJ mol^−1^ per contact). The sum of each AIP contact free energy in chloroform solution is −20 kJ mol^−1^, roughly double the total in water (−10 kJ mol^−1^), which is consistent with the much higher binding affinity observed in chloroform solution for this complex (*K*_a_ = 10^6^ M^−1^ compared with 10^2^ M^−1^ in water).^35^ A quantitative treatment of the overall free energy of binding for systems of this complexity will require a more sophisticated treatment of the relationship between the free energy contributions from multiple interaction points, the contribution of conformational entropy changes, the consequences of sub-optimal orientations of interacting groups, and the effects of solvent packing arrangements in the cavity. Nevertheless, the treatment described here provides detailed insight into the precise contribution of each non-covalent interaction to the overall free energy of complexation, providing a powerful tool for use in supramolecular design and understanding the role of solvent.

## Conclusions

The molecular electrostatic potential surface (MEPS) of a molecule calculated using density functional theory provides information on the properties of polar interactions with other molecules in the condensed phase. Correlations between maxima and minima on the MEPS with experimentally determined H-bond parameters (*α* and *β*) allow translation of the MEPS calculated for an isolated molecule in the gas phase into a set of discrete atomic interaction points (AIP) that provide a complete description of the thermodynamic properties of the non-covalent interactions that molecule can make with other molecules. Specifically, the AIP description of a molecule can be used to calculate solvation energies and observables like the partition coefficient for transfer between two different solvents.

By using high electron density MEPS that lie inside the van der Waals surface (0.0300 and 0.0104 e bohr^−3^ electron density isosurfaces), it is possible to obtain accurate descriptions of the properties of polar H-bond and acceptor sites, whereas non-polar sites are better described by an MEPS that lies close the van der Waals surface (0.0020 e bohr^−3^ electron density isosurface). Polar H-bonding sites and σ-holes are clearly identified as local maxima and minima on the high electron density MEPS, and linear correlations were observed between the MEP values and the experimental H-bond parameters. For non-polar sites, an approach based on molecular orbitals was used to assign the locations of the AIPs that represent π-systems and halogens, and parameterisation used phase transfer free energies as well as experimentally measured H-bond parameters. Compared with the original SSIP model, the new AIP model provides a more accurate description of H-bond parameters for a wide variety of different compounds, a more accurate description of the location of the interaction sites in three-dimensional space around the molecule, and an improved description of solvation free energies as measured by experimental data on the partition between water and *n*-hexadecane of more than a thousand different compounds.

The use of AIPs to describe the non-covalent interaction properties of molecules provides a new method for analysing the factors that contribute to the thermodynamic stability of intermolecular complexes. By mapping AIPs onto the three-dimensional structure of a complex, it is possible to identify intermolecular interactions as close proximity between two AIPs. The AIP contacts can be used to calculate the free energy contribution of each intermolecular interaction to the stability of the complex and to make predictions about the effect of solvent on these interactions.

## Data availability

All supporting data is provided in the ESI.†

## Author contributions

The manuscript was written through contributions of all authors.

## Conflicts of interest

There are no conflicts to declare.

## Supplementary Material

SC-015-D3SC05690B-s001
