# Supplementary material for: An atomic surface site interaction point description of non-covalent interactions
Source: Chem Sci. 2023 Dec 8;15(1):160–70. doi: 10.1039/d3sc05690b (PMC10732136; doi:10.1039/d3sc05690b)
Supplement: SC-015-D3SC05690B-s001 [file SC-015-D3SC05690B-s001.pdf]

Electronic Supplementary Material (ESI) for Chemical Science.
This journal is © The Royal Society of Chemistry 2023

An Atomic Surface Site Interaction Point Description of Non-Covalent Interactions

Maria Chiara Storer, Katarzyna J. Zator, Derek P. Reynolds, Christopher A. Hunter*

Yusuf Hamied Department of Chemistry, University of Cambridge, CB2 1EW, U.K
Email: herchelsmith.orgchem@ch.cam.ac.uk

Contents

1 Analysis of α and β Parameters **2**
1.1 σ-Hole Data 2
1.2 I_2 Complexes 9
1.3 Statistical Correction of β Parameters 10
1.4 Halogen β parameters. 12
1.5 Calculation of MEPS 13
1.6 Relationship between β and E_{min} 14
1.7 Linear Regression with Constant Slope and Variable Intercept 19

2 Assignment of Atomic Surface Site Interaction Points **19**
2.1 Atom Types 19
2.2 Atomic Surface Areas 20
2.3 Lone Pair AIP Locations 21
2.4 Fluorine AIP Locations 21
2.5 Hydrogen AIP Locations 22
2.6 σ-Hole AIP Locations 22
2.7 Non-polar AIP Locations 23

3 Free Energy of Transfer **24**
3.1 Data 24
3.2 Free Energy of Transfer and the Number of Alkyl Hydrogens 72
3.3 f value for Various Atom Types 75

4 Host-guest complexes AIP pairings **75**
4.1 Tables of host-guest interactions 75

1 Analysis of α and β Parameters

1.1 σ-Hole Data

Donor	Acceptor	Solvent	log$K$/M^{-1}	σ	Ref.
I-Cl	Pyridine N-oxide	CCl_4	4.6	1	*(1)*
I-Cl	Diphenyl sulfoxide	CCl_4	2.61	1	*(2)*
I-Cl	Triphenylphosphine oxide	CCl_4	3.48	1	*(2)*
I-Cl	1,3,5-Trioxan	CCl_4	0.38	1	*(3)*
I-Cl	Trimethyl orthoformate	CCl_4	1.06	1	*(3)*
I-Cl	Di-n-Butyl ether	CCl_4	1.12	1	*(3)*
I-Cl	1,4-Dioxan	CCl_4	1.44	1	*(3)*
I-Cl	1,2-Dimethoxyethane	CCl_4	1.46	1	*(3)*
I-Cl	15-Crown-5	CCl_4	2.03	1	*(3)*
I-Cl	Tetrahydrofuran	CCl_4	1.86	1	*(3)*
I-Cl	Diphenyl ether	CCl_4	-0.04	1	*(3)*
I-Cl	anisole	CCl_4	0.28	1	*(3)*
I-Cl	Dibenzyl ether	CCl_4	0.88	1	*(3)*
I-Cl	N,N-Dimethylformamide	CCl_4	2.73	1	*(4)*
I-Cl	N,N-Dimethylacetamide	CCl_4	3.23	1	*(4)*
I-Cl	Tetramethylurea	CCl_4	3.22	1	*(4)*
I-Cl	N,N,2,2-Tetramethylpropionamide	CCl_4	2.58	1	*(4)*
I-Cl	Methyl acetate	CCl_4	0.86	1	*(4)*
I-Cl	Acetone	CCl_4	1.25	1	*(4)*
I-Cl	Ethyl methyl ketone	CCl_4	1.23	1	*(4)*
I-Cl	Ethyl formate	CCl_4	0.23	1	*(4)*
I-Cl	Acetaldehyde	CCl_4	0.52	1	*(4)*
I-Cl	N,N-Dimethylbenzamide	CCl_4	2.76	1	*(4)*
I-Cl	Cyclobutanone	CCl_4	1.12	1	*(5)*
I-Cl	Cyclopentanone	CCl_4	1.54	1	*(5)*
I-Cl	Cyclohexanone	CCl_4	1.64	1	*(5)*
I-Cl	Cycloheptaneanone	CCl_4	1.74	1	*(5)*
I-Cl	e-caprolactone	CCl_4	1.62	1	*(5)*
I-Cl	Ethyl butanoate	CCl_4	0.96	1	*(6)*
I-Cl	Pyrrolidin-2-one	CCl_4	2.48	1	*(6)*
I-Cl	N-methylpyrrolidone	CCl_4	2.78	1	*(6)*
I-Cl	N-Methyl-e-Caprolactam	CCl_4	2.91	1	*(6)*
I-Cl	N,N-Diethylacetamide	CCl_4	3.1	1	*(6)*
I-CN	Benzophenone	CCl_4	0.53	1	*(7)*
I-CN	Diphenyl sulfoxide	CCl_4	1.6	1	*(7)*
I-CN	Diethyl ether	CCl_4	0.61	1	*(7)*
I-CN	Tetrahydrofuran	CCl_4	1.15	1	*(7)*
I-CN	Dimethyl sulfoxide	CCl_4	1.97	1	*(8)*
I-CN	Tetramethylene sulfoxide	CCl_4	1.99	1	*(8)*
I_2	t-Butyl methyl ether	cyclohexane	0.07	2	*(2)*
I_2	Diethyl ether	cyclohexane	-0.05	2	*(2)*
I_2	Ethyl n-propyl ether	cyclohexane	-0.11	2	*(2)*
I_2	t-Butyl ethyl ether	cyclohexane	-0.27	2	*(2)*
I_2	Di-n-propyl ether	cyclohexane	-0.16	2	*(2)*

I_2	Di-n-butyl ether	heptane	-0.16	2	(*2*)
I_2	Di-i-propyl ether	cyclohexane	-0.21	2	(*2*)
I_2	Dimethoxymethane	CCl_4	-0.33	2	(*2*)
I_2	1,2-Dimethoxyethane	CCl_4	-0.14	2	(*2*)
I_2	1,1,1-Trimethoxymethane	CCl_4	-0.64	2	(*2*)
I_2	Propylene oxide	heptane	-0.03	2	(*2*)
I_2	Oxetane	heptane	0.59	2	(*2*)
I_2	Tetrahydrofuran	heptane	0.4	2	(*2*)
I_2	Tetrahydropyran	heptane	0.4	2	(*2*)
I_2	1,3-Dioxan	CCl_4	-0.28	2	(*2*)
I_2	1,4-Dioxan	cyclohexane	-0.11	2	(*2*)
I_2	1,3,5-Trioxan	CCl_4	-0.81	2	(*2*)
I_2	2-Methyltetrahydrofuran	heptane	0.47	2	(*2*)
I_2	12-Crown-4	CCl_4	-0.04	2	(*2*)
I_2	15-Crown-5	CCl_4	-0.18	2	(*2*)
I_2	Acetaldehyde	heptane	-0.36	2	(*2*)
I_2	Benzaldehyde	heptane	-0.28	2	(*2*)
I_2	Ethyl trifluoroacetate	heptane	-1	2	(*2*)
I_2	Ethyl trichloroacetate	heptane	-0.62	2	(*2*)
I_2	Methyl formate	heptane	-0.36	2	(*2*)
I_2	Ethyl formate	heptane	-0.31	2	(*2*)
I_2	Dimethyl carbonate	heptane	-0.25	2	(*2*)
I_2	Methyl benzoate	heptane	-0.21	2	(*2*)
I_2	Diethyl carbonate	heptane	-0.21	2	(*2*)
I_2	Ethyl Methyl carbonate	heptane	-0.21	2	(*2*)
I_2	Ethyl pivalate	heptane	-0.17	2	(*2*)
I_2	Ethyl benzoate	heptane	-0.15	2	(*2*)
I_2	Methyl acetate	heptane	-0.12	2	(*2*)
I_2	Ethyl isobutyrate	heptane	-0.1	2	(*2*)
I_2	Ethyl propionate	heptane	-0.04	2	(*2*)
I_2	Ethyl acetate	heptane	-0.03	2	(*2*)
I_2	Ethyl cyclopropanecarboxylate	heptane	0	2	(*2*)
I_2	Ethyl butanoate	CCl_4	0.04	2	(*2*)
I_2	e-Caprolactone	CCl_4	0.22	2	(*2*)
I_2	1,1,1-Trichloroacetone	heptane	-0.96	2	(*2*)
I_2	1,1,1-Trifluoroacetone	heptane	-0.6	2	(*2*)
I_2	3-Trifluoromethylacetophenone	heptane	-0.24	2	(*2*)
I_2	Benzophenone	heptane	-0.07	2	(*2*)
I_2	4-Chloroacetophenone	heptane	-0.07	2	(*2*)
I_2	Cyclobutanone	heptane	-0.02	2	(*2*)
I_2	Diethyl ketone	heptane	0.01	2	(*2*)
I_2	Ethyl methyl ketone	heptane	0.01	2	(*2*)
I_2	Isopropyl methyl ketone	heptane	0.03	2	(*2*)
I_2	Acetone	heptane	0.05	2	(*2*)
I_2	Acetophenone	heptane	0.06	2	(*2*)
I_2	3-Methoxyacetophenone	heptane	0.14	2	(*2*)
I_2	3-Methylacetophenone	heptane	0.11	2	(*2*)
I_2	4-Methylacetophenone	heptane	0.16	2	(*2*)
I_2	Cyclopentanone	heptane	0.23	2	(*2*)
I_2	4-Methoxyacetophenone	heptane	0.29	2	(*2*)

I_2	Cycloheptaneanone	CCl_4	0.39	2	(*2*)
I_2	Cyclohexanone	CCl_4	0.4	2	(*2*)
I_2	Dimethylcarbamoyl chloride	heptane	-0.41	2	(*2*)
I_2	N,N-dimethyltrichloroacetamide	heptane	-0.35	2	(*2*)
I_2	N,N-dimethyltrifluoroacetamide	heptane	-0.21	2	(*2*)
I_2	N,N-dimethylchloroacetamide	CCl_4	0.34	2	(*2*)
I_2	1-(3'-Chlorophenyl)-2-pyrrolidone	CCl_4	0.41	2	(*2*)
I_2	1-(4'-Chlorophenyl)-2-pyrrolidone	CCl_4	0.47	2	(*2*)
I_2	N,N-diisopropylpivalamide	heptane	0.48	2	(*2*)
I_2	1-Phenyl-2-pyrrolidone	CCl_4	0.64	2	(*2*)
I_2	1-(3'-Methoxylphenyl)-2-pyrrolidone	CCl_4	0.64	2	(*2*)
I_2	1-(4'-Ethylphenyl)-2-pyrrolidone	CCl_4	0.7	2	(*2*)
I_2	1-(4'-Methylphenyl)-2-pyrrolidone	CCl_4	0.73	2	(*2*)
I_2	1-(2'-Chlorophenyl)-2-pyrrolidone	CCl_4	0.73	2	(*2*)
I_2	1-(4'-Methoxylphenyl)-2-pyrrolidone	CCl_4	0.78	2	(*2*)
I_2	N,N-dimethylformamide	heptane	0.81	2	(*2*)
I_2	N,N-dimethylpivalamide	heptane	0.83	2	(*2*)
I_2	N,N-dimethylbenzamide	heptane	0.91	2	(*2*)
I_2	N,N-dimethylacetamide	heptane	1.18	2	(*2*)
I_2	Pyrrolidin-2-one	CCl_4	1.2	2	(*2*)
I_2	N,N-diethylacetamide	heptane	1.27	2	(*2*)
I_2	N-methylpyrrolidone	heptane	1.3	2	(*2*)
I_2	N-methyl-e-caprolactam	heptane	1.35	2	(*2*)
I_2	N-methylpiperidone	heptane	1.47	2	(*2*)
I_2	Tetraethylurea	heptane	1.04	2	(*2*)
I_2	Tetramethylurea	heptane	1.16	2	(*2*)
I_2	Di-n-butyl sulfoxide	cyclohexane	1.93	2	(*2*)
I_2	Tetramethylene sulfoxide	CCl_4	1.72	2	(*2*)
I_2	Dimethyl sulfoxide	cyclohexane	1.56	2	(*2*)
I_2	Diphenyl sulfoxide	CCl_4	1.1	2	(*2*)
I_2	Sulfolane	CCl_4	-0.12	2	(*2*)
I_2	Diethyl sulfite	CCl_4	-0.16	2	(*2*)
I_2	Trimethylphosphine oxide	CCl_4	2.72	2	(*2*)
I_2	Triethylphosphine oxide	CCl_4	2.76	2	(*2*)
I_2	Tri-n-butylphosphine oxide	cyclohexane	2.75	2	(*2*)
I_2	Tri-n-octylphosphine oxide	cyclohexane	2.77	2	(*2*)
I_2	Hexamethylphosphoric acid triamide	CCl_4	2.55	2	(*2*)
I_2	Triphenylphosphine oxide	CCl_4	2.08	2	(*2*)
I_2	Ethyl tetraethylphosphorodiamidate	CCl_4	1.93	2	(*2*)

I_2	Diethyl methylphosphonate	cyclohexane	1.57	2	(*2*)
I_2	Tri-n-butyl phosphate	cyclohexane	1.33	2	(*2*)
I_2	Diethyl chloromethylphosphonate	CCl_4	0.87	2	(*2*)
I_2	Diethyl dichloromethylphosphonate	CCl_4	0.51	2	(*2*)
I_2	Pyridine N-oxide	cyclohexane	2.4	2	(*2*)
CCl_4	Tetrahydrofuran	cyclohexane	-1.1	4	(*9*)
CCl_4	Tetrahydropyran	cyclohexane	-0.89	4	(*9*)
CCl_4	Di-n-Butyl ether	cyclohexane	-1.4	4	(*2*)
CCl_4	N,N-Dimethylacetamide	cyclohexane	-0.44	4	(*10*)
CCl_4	N-Methylpyrrolidone	cyclohexane	-0.39	4	(*10*)
CBr_4	Tetrahydrofuran	heptane	-0.34	4	(*9*)
CBr_4	Tetrahydropyran	heptane	-0.03	4	(*9*)
$Cl_2C{=}CCl_2$	Tetrahydrofuran	cyclohexane	-0.82	4	(*9*)
$Cl_2C{=}CCl_2$	Tetrahydropyran	cyclohexane	-1.22	4	(*9*)
IC_6F_5	Di-n-Butyl sulfoxide	cyclohexane	0.3	1	(*11*)
IC_6F_5	Tri-n-Butylphosphine oxide	cyclohexane	1.08	1	(*11*)
p-C#CPh -IC_6F_4	tri n-butyl phosphine oxide	cyclohexane	0.75	4	(*11*)
p-Ph -IC_6F_4	tri n-butyl phosphine oxide	cyclohexane	0.59	4	(*11*)
p-OCH3 -IC_6F_4	tri n-butyl phosphine oxide	cyclohexane	0.52	4	(*11*)
N(CH2)5 -IC_6F_4	tri n-butyl phosphine oxide	cyclohexane	0.11	4	(*11*)
I-C≡C-C≡N	N,N-Diethylacetamide	CCl_4	1.7	1	(*12*)
I-C≡C-C≡N	triphenylphosphine oxide	CCl_4	2.34	1	(*12*)
I-C≡C-Ph	N,N-Diethylacetamide	CCl_4	0.2	1	(*12*)
I-C≡C-Ph	Triphenylphosphine oxide	benzene	0.39	1	(*12*)
I-C≡C-Ph	Hexamethylphosphoric triamide	benzene	0.78	1	(*12*)
I-C≡C-COOEt	triphenylphosphine oxide	benzene	1.04	1	(*12*)
I-C≡C-I	triphenylphosphine oxide	benzene	0.19	1	(*12*)
I-C≡C-I	Hexamethylphosphoric triamide	benzene	0.72	1	(*12*)
I-$(CF_2)_5CF_3$	Tri-n-Butylphosphine oxide	CCl_4	0.6	1	(*11*)
I-$(CF_2)_5CF_3$	Tri-n-Butylphosphine oxide	benzene	0.7	1	(*11*)

Table S1 log K data for complexation of σ-holes with oxygen acceptors in non-polar solvents (σ indicates the number of degenerate interaction sites).

Solvent	α_S	β_S
benzene	1.1	1.6
CCl4	1.4	0.6
heptane	1.2	0.6
cyclohexane	1.2	0.6

Table S2 α_S and β_S parameters of the non-polar solvents for the for complexation of σ-holes with oxygen acceptors.

Acceptor	β
1,1,1-trifluoroacetone	2.9
1,1,1-trichloroacetone	3.1
1,3,5-trioxan	3.1
diphenyl ether	3.1
ethyl trifluoroacetate	3.2
anisole	3.3
ethyl trichloroacetate	3.4
1,1,1-trimethoxymethane	4.3
trimethyl orthoformate	4.3
dimethoxymethane	4.4
acetaldehyde	4.5
ethyl formate	4.5
1,3-dioxan	4.5
methyl formate	4.5
dibenzyl ether	4.7
3-trifluoromethylacetophenone	4.7
benzaldehyde	4.8
methyl benzoate	4.9
ethyl methyl carbonate	4.9
diethyl sulfite	4.9
dimethyl carbonate	4.9
di-n-butyl ether	5.0
1,4-dioxan	5.0
diethyl carbonate	5.0
methyl acetate	5.1
4-chloroacetophenone	5.1
ethyl benzoate	5.1
propylene oxide	5.2
di-n-propyl ether	5.2
ethyl n-propyl ether	5.2
dimethylcarbamoyl chloride	5.3
diethyl ether	5.3
ethyl acetate	5.3
cyclobutanone	5.3
benzophenone	5.4
di-i-propyl ether	5.4
ethyl pivalate	5.4
diethyl ketone	5.4
n,n-dimethyltrifluoroacetamide	5.4
isopropyl methyl ketone	5.5
ethyl propionate	5.5
ethyl isobutyrate	5.5
ethyl butanoate	5.5
3-methylacetophenone	5.5
1,2-dimethoxyethane	5.6
t-butyl methyl ether	5.6
12-crown-4	5.6
ethyl cyclopropanecarboxylate	5.6

15-crown-5	5.6
3-methoxyacetophenone	5.6
t-butyl ethyl ether	5.6
tetrahydropyran	5.7
acetone	5.7
n,n-dimethyltrichloroacetamide	5.7
ethyl methyl ketone	5.7
acetophenone	5.7
4-methylacetophenone	5.8
tetrahydrofuran	5.9
cyclopentanone	6.0
2-methyltetrahydrofuran	6.0
4-methoxyacetophenone	6.0
cyclohexanone	6.1
sulfolane	6.2
oxetane	6.2
cycloheptanone	6.2
1-(3'-chlorophenyl)-2-pyrrolidone	6.4
1-(4'-chlorophenyl)-2-pyrrolidone	6.5
e-caprolactone	6.7
n,n-dimethylchloroacetamide	6.9
1-(3'-methoxylphenyl)-2-pyrrolidone	7.0
1-phenyl-2-pyrrolidone	7.1
1-(4'-methylphenyl)-2-pyrrolidone	7.3
1-(4'-ethylphenyl)-2-pyrrolidone	7.3
diphenyl sulfoxide	7.5
1-(4'-methoxylphenyl)-2-pyrrolidone	7.5
n,n-dimethylformamide	7.6
n,n-diisopropylpivalamide	7.6
n,n,2,2-tetramethylpropionamide	7.8
n,n-dimethylpivalamide	7.8
diethyl dichloromethylphosphonate	7.8
1-(2'-chlorophenyl)-2-pyrrolidone	7.8
n,n-dimethylbenzamide	7.8
n,n-diethylacetamide	8.2
n,n-dimethylacetamide	8.4
n-methylpyrrolidone	8.4
tetramethylurea	8.4
diethyl chloromethylphosphonate	8.5
tetraethylurea	8.5
tetramethylene sulfoxide	8.6
tri-n-butyl phosphate	8.6
dimethyl sulfoxide	8.7
n-methyl-e-caprolactam	8.7
di-n-butyl sulfoxide	8.7
n-methylpiperidone	8.8
pyrrolidin-2-one	8.9
ethyl tetraethylphosphorodiamidate	9.0
pyridine n-oxide	9.1
diethyl methylphosphonate	9.1

triphenylphosphine oxide	10.1
tri-n-butylphosphine oxide	10.2
triethylphosphine oxide	10.6
tri-n-octylphosphine oxide	10.7
trimethylphosphine oxide	10.7
hexamethylphosphoric triamide	11.0

Table S3 β values of the oxygen acceptors involved in the complexation with σ-holes in non-polar solvents.

1.2 I_2 Complexes

Figure S1 shows a comparison of the calculated log$K$ (logK_{calc}) and the experimental log$K$ (logK_{expt}) for the 1:1 complexes of I_2 with different oxygen acceptors. The calculated log$K$ is obtained with Equ. 2 with an α for I_2 = 2.3.

The plot shows that Equ. 2 does not accurately describe the behaviour of complexes involving I_2, perhaps because the low-lying $\sigma*$ orbitals of I_2 give rise to a high degree of covalent character in the interaction even when oxygen acceptors are involved.

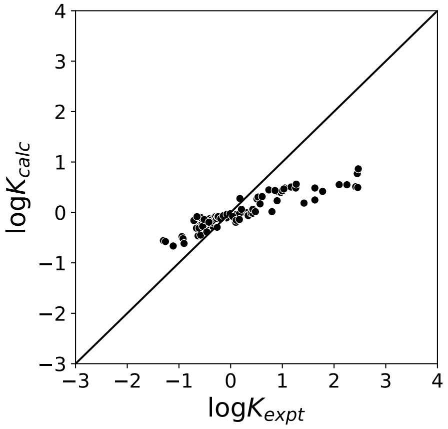

Figure S1 Comparison of the calculated log$K$ (logK_{calc}) and the experimental log$K$ (logK_{expt}) for the 1:1 complexes of I_2 with different oxygen acceptors. The calculated log$K$ is obtained with Equ. 2 with an α for I_2 = 2.3.

1.3 Statistical Correction of β Parameters

For some compounds, the experimentally determined value of β was statistically corrected to reflect the number of degenerate H-bond acceptors present. Table S4 lists the molecules for which the value of β differs from that published in ref. (*13*).

name	original β	corrected β	Note
3-chloronitrobenzene	3.4	2.7	b
4-chloronitrobenzene	3.6	2.9	b
bis(2-chloroethyl) ether	3.7	3.0	a
nitromethane	3.7	3.0	b
nitroethane	3.8	3.1	b
4-nitrophenyl acetate	3.8	3.1	b
4-nitrotoluene	3.8	3.1	b
4-nitrobenzaldehyde	3.9	3.2	b
nitrobenzene	3.9	3.2	b
2-methyl-2-nitropropane	4.0	3.3	b
2-nitropropane	4.0	3.3	b
1,2-dimethyl-4-nitrobenzene	4.1	3.4	b
4-nitroanisole	4.2	3.5	b
biacetyl	4.2	3.6	b
2-chloroethyl ethyl ether	4.3	4.0	a
2-benzyl-1,2-benzothiazol-3(2h)-one 1,1-dioxide	4.6	3.9	a
1-(4-nitrophenyl)ethanone	4.6	3.9	b
dibenzyl ether	4.7	4.5	a
diethyl sulphate	4.8	4.1	a
n,n-diethyl-4-nitroaniline	5.1	4.4	b
n-benzyl-n-methylbenzenesulfonamide	5.2	4.5	b
9,10 phenantrequinone	5.3	4.6	b
ethyl methanesulfonate	5.3	4.6	b
N,*N*-dimethylbenzenesulfonamide	5.8	5.1	b
diphenyl sulphone	5.9	5.2	b
N-methylmethanesulfonamide	5.9	5.2	b
N,*N*-dimethylmethanesulfonamide	6.0	5.3	b
tetrahydrothiophene 1,1-dioxide	6.2	5.5	b
dimethyl sulphone	6.2	5.5	b
N,*N*,*N*',*N*'-tetraethylsulfamide	6.3	5.6	b
dibutyl sulphone	6.4	5.7	b
3-aminopropanenitrile	6.1	5.9	c
2-methyl-5-(methylsulfanyl)-1,3,4-thiadiazole	7.5	6.4	b
2,5-dimethyl-1,3,4-thiadiazole	7.2	6.8	b
N-[(4-methylphenyl)sulfonyl]-S,S-diphenyl-sulfilimine	7.8	7.1	b
4-butyl-4H-1,2,4-triazole	8.7	8.2	b
2,2,2-trimethyl-1-[(4-methylphenyl)sulfonyl]diazan-2-ium-1-ide	8.9	8.2	b
2,2,2-trimethyl-1-(octylsulfonyl)diazan-2-ium-1-ide	9.5	8.8	b

Table S4 Corrected β values Note a) competition with other functional groups, but the contribution of just the ether was obtained from IR in ref. (*14*) b) statistically corrected by RTln2 to take into account two equivalent sites c) competition with other functional groups, but the contribution of just the ether was obtained from IR in ref. (*15*)

1.4 Halogen β parameters.

**Compound**	β
CHLORINE	
1,1,1-trichloroethane	0.5
tetrachloromethane	0.6
chloroform	0.8
dichloromethane	1.3
1,1-dichloroethane	1.5
1,2-dichloroethane	1.7
1,3-dichloropropane	1.9
1,4-dichlorobutane	2.0
1,5-dichloropentane	2.2
1-chlorobutane	2.2
1-chloropentane	2.2
2-chloropropane	2.4
2-chloro-2-methylpropane	2.4
chlorocyclohexane	2.5
1-chloroadamantane	2.7
BROMINE	
dibromomethane	1.5
1,2-dibromoethane	1.7
1,3-dibromopropane	1.9
bromocyclopropane	2.0
1,4-dibromobutane	2.0
1-bromopropane	2.2
bromoethane	2.2
1-bromobutane	2.3
1-bromopentane	2.3
2-bromopropane	2.4
bromocyclohexane	2.5
2-bromo-2-methylpropane	2.6
1-bromoadamantane	2.7
IODINE	
1,2-diiodoethane	1.6
diiodomethane	1.6
1,3-diiodopropane	1.9
iodoethane	2.0
1,4-diiodobutane	2.0
iodomethane	2.0
1-iodopentane	2.2
2-iodopropane	2.2
2-iodo-2-methylpropane	2.3
iodocyclohexane	2.4
1-iodoadamantane	2.6

Table S5 β parameters for halogen acceptors from ref. (*16*), which have been statistically corrected taking into account the number of acceptor sites.

1.5 Calculation of MEPS

MEPS calculations were carried out using NWChem 7.0.2 on a cluster of 8 eight-core nodes (Intel Xeon E5-2650 v2 2.60GHz Ivy Bridge CPUs, 128 GB RAM per node). A standard geometry optimisation was carried out on each molecule using a DRIVER module with up to 600 iterations and a convergence criterion of 1e-08 (B3LYP functional and 6-31G* basis set, or 6-31G** basis set for bromine and iodine without effective core potentials). The MEPS were then calculated using the espiso function in NWChem with the following parameters:

0.0020 isosurface: padding: 4.0, iso_surf: 0.002, step_size: 0.066, tol: 3e-05

0.0104 isosurface: padding: 4.0, iso_surf: 0.0104, step_size: 0.04, tol: 1e-4

0.0300 isosurface: padding: 4.0, iso_surf: 0.0300, step_size: 0.04, tol: 1e-4

For each structure, seven different rotations were carried out (combinations of 45° and 120° in one plane and 10° and 100° in another), and the MEPS calculations were repeated. The seven rotated MEPS were then superimposed to obtain high resolution MEPS for analysis.

1.6 Relationship between β and E_{min}

Figure S2 shows the relationship of the β and the E_{min} of the MEP calculated with DFT with the B3LYP/6-31G* for polar acceptor functional groups involving nitrogen, oxygen, fluorine and sp^2 sulfur.

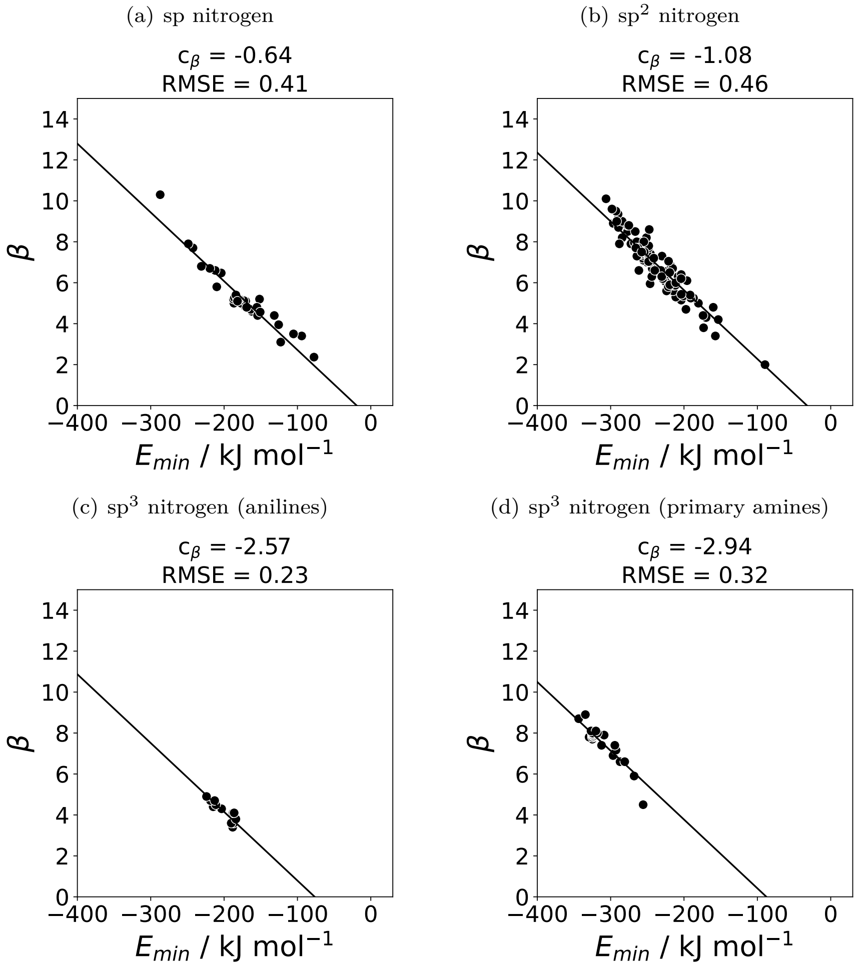

Figure S2 Relationship between β and the E_{min} of the MEP calculated for polar acceptor functional groups at the 0.0300 e bohr^{-3} with DFT, calculated with the B3LYP/6-31G* (B3LYP/6-311G** for sulfur). The lines are the lines of best fit keeping the slope constant amongst the different functional groups (m_β =-0.0336 / kJ mol^{-1}).

(a) sp^3 nitrogen (secondary amines)

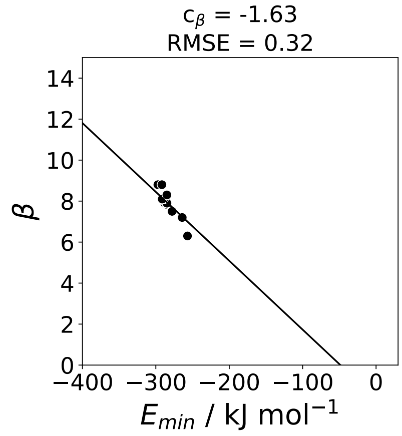

(b) sp^3 nitrogen (tertiary amines)

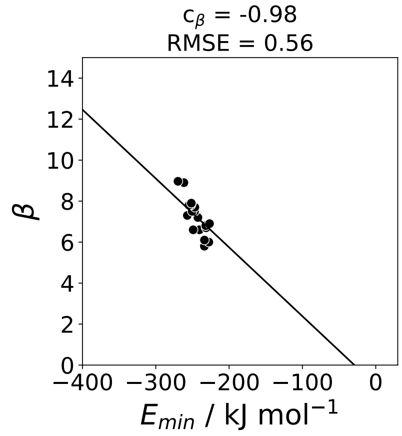

(c) sp^2 oxygen (ketones, esters, aldehydes, cabonates, nitro groups)

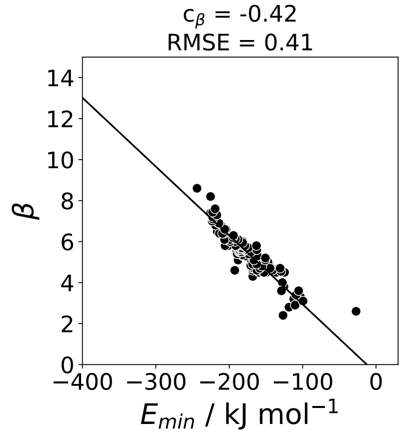

(d) sp^2 oxygen (amides, ureas, vinylogous amides)

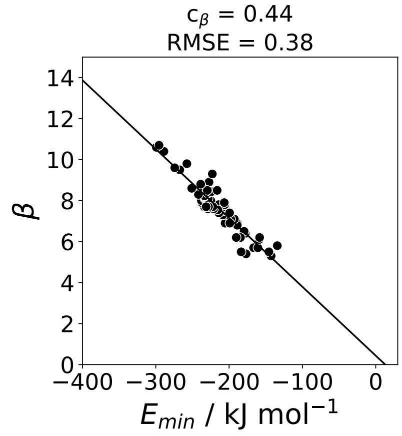

(e) sp^2 oxygen (nitrogen oxides)

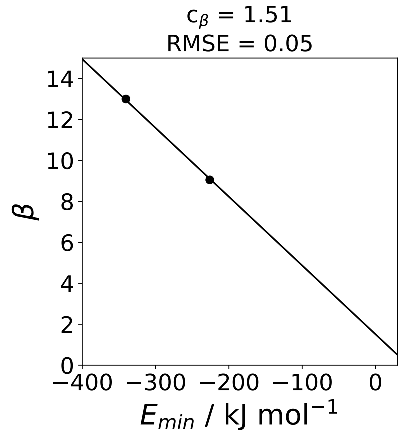

(f) sp^2 oxygen (nitro)

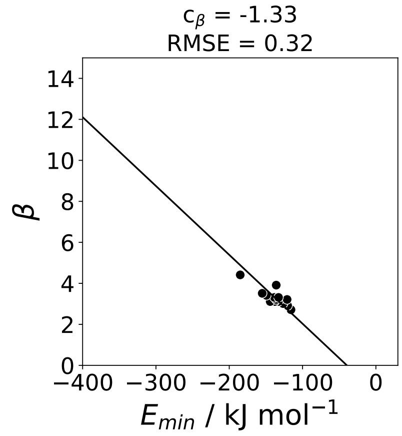

Figure S2 Continued here.

(g) sp^2 oxygen (S=O)

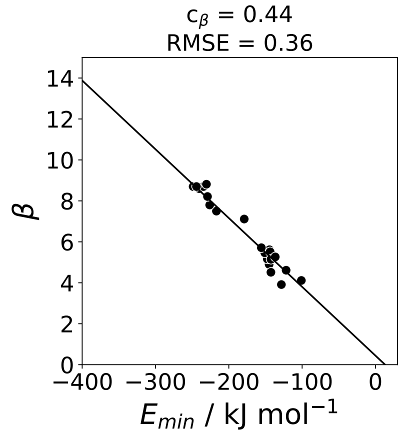

(h) sp^2 oxygen (P=O)

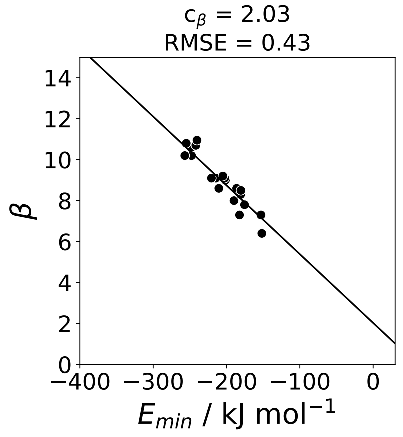

(i) sp^3 oxygen (ethers, phenols, epoxides)

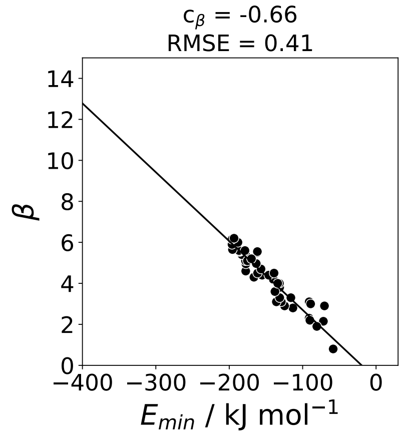

(j) sp^3 oxygen (alcohols)

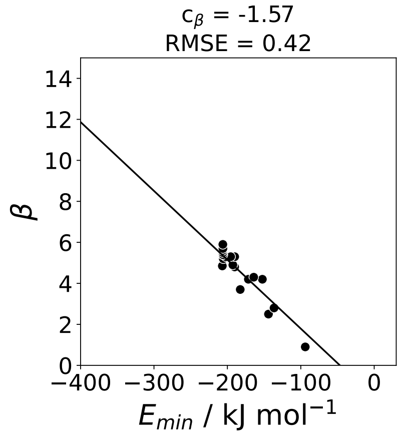

(k) fluorine

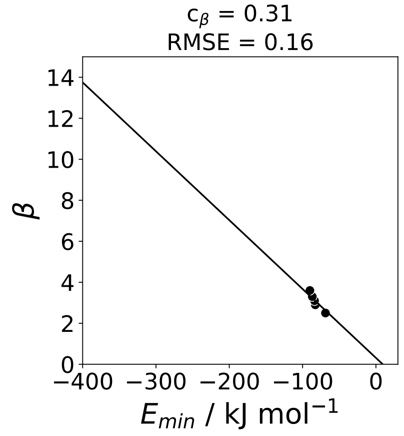

Figure S2 Continued here.

(l) sp^2 sulfur (thiones)

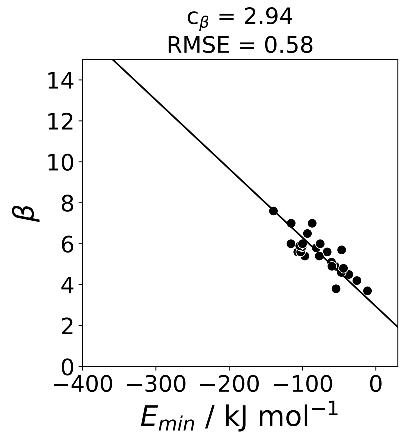

(m) sp^2 sulfur (thiophosphines)

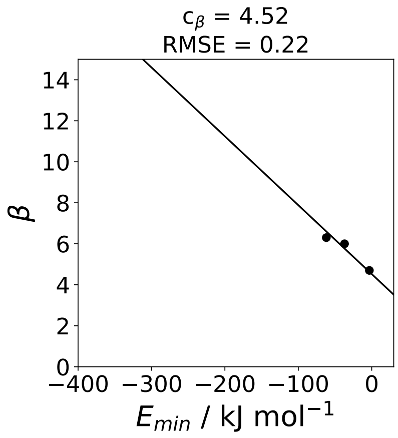

Figure S2 Continued here.

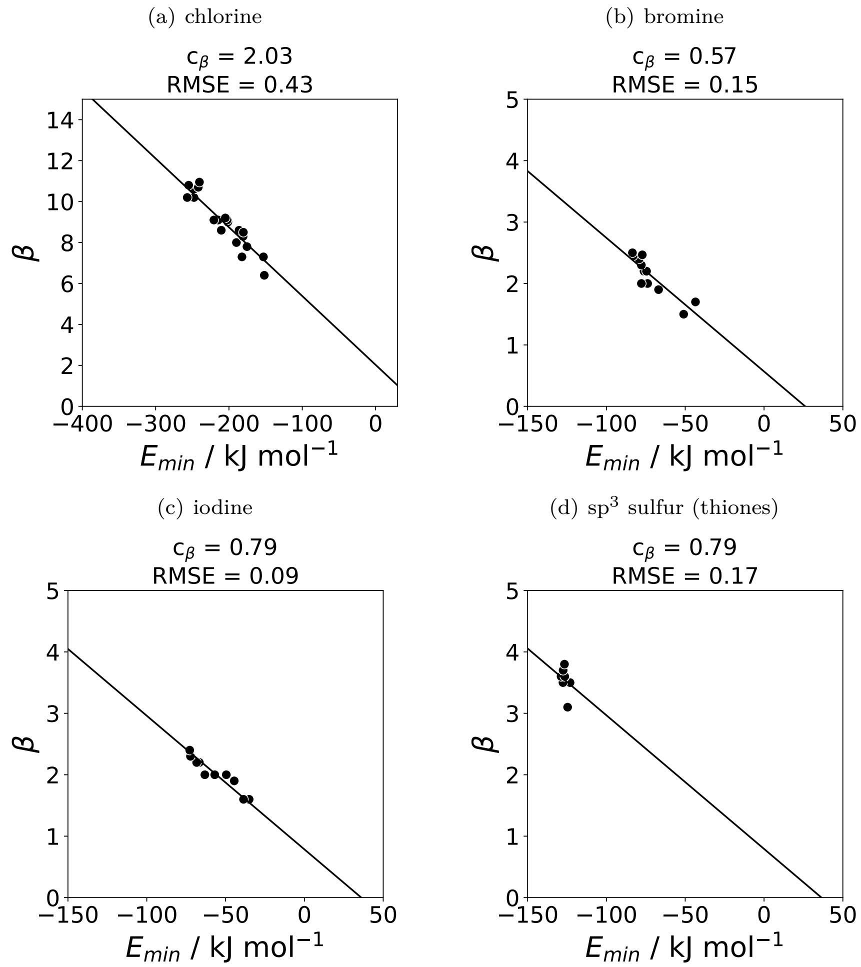

Figure S3 Relationship between the β and E_{min} of the MEP calculated for non-polar acceptor functional groups at the 0.0020 e bohr^{-3} surface with DFT, with the B3LYP/6-31G* (B3LYP/6-311G** for bromine iodine). The lines are the lines of best fit keeping the slope constant amongst the different functional groups (m_β = -0.0232 / kJ mol^{-1}).

1.7 Linear Regression with Constant Slope and Variable Intercept

The parameter $\mathbf{y}$ is related to x by the linear equation shown below.

$$\mathbf{y} = \mathrm{m}\mathbf{x} + \mathrm{c} \tag{S1}$$

The error of this equation is:

$$\epsilon = \frac{1}{N}\sum_{i}^{N}(y_i - mx_i + c)^2 \tag{S2}$$

We want to find the optimal intercept c if the slope is known. This will allow us to handle data that have a constant slope but varying intercept. To find the optimal c we rewrite the equation as

$$\epsilon = \frac{1}{N}\sum_{i}^{N}\left(a_i^2 - 2a_i c + c^2\right) \tag{S3}$$

Where a_i is y_i-mx_i.

We can also write this as:

$$\epsilon = \frac{1}{N}\left(\sum_{i}^{N}a_i^2 - 2c\sum_{i}^{N}a_i + c^2\right) \tag{S4}$$

To find the c which minimises the error we differentiate with respect to c and set this to zero.

The c which minimises the error is

$$c = \frac{1}{N}\sum_{i}^{N}a_i = \frac{1}{N}\sum_{i}^{N}(y_i - mx_i) \tag{S5}$$

Having found the optimal intercept for a given slope, we can loop through different possible slopes, find the ideal intercept and calculate the RMSE. We then select the slope that gives the lowest average RMSE for the different systems of interest.

2 Assignment of Atomic Surface Site Interaction Points

2.1 Atom Types

An extended set of atom type based on the SYBYL system was used (Table S6). We employed a sub-graph matching method: the molecular graph represents the connectivity of the molecule where the nodes are the molecule element and SYBYL atom type and the edges contain information on the bond order (*17*). The sub-graph matching algorithm employed was the VF2 algorithm (*18*) implemented using NetworkX (*19*).

2.2 Atomic Surface Areas

The calculated Molecular Electrostatic Potential Surface (MEPS) of a molecule is described by a set of points. Each point was assigned to the closest atom, defined as the atom with the smallest difference between the distance to the MEPS point and the van der Waals radius as defined in the Medeleev library (*20*). The patch of MEPS points assigned to each atom was then used to calculate the atomic surface area using the ConvexHull algorithm in the SciPy library (*21*). For each atom type, a minimum surface area parameter (A_{min}) was defined using the 0.0020 e bohr^{-3} electron density isosurface to eliminate atoms that are too sterically hindered to make intermolecular contacts, and these atoms were not assigned any AIPs (see Table S1). In order to minimise the through-space interactions with neighbouring groups, the edge region of each surface patch was eliminated. This region was defined by the lowest 20% of local densities of neighbouring MEPS points. The local density of MEPS points was calculated for each point in the patch by counting the number of points within a 1 Å radius. The remaining points were used to assign AIPs.

**Atom Type**	**Description**	A_{min} / **Å**^{2}
C.1	sp carbon	5.00
C.2	sp2 carbon	2.50
C.3	sp3 carbon	-
C.ar	aromatic carbon	1.85
C.cat	amidine carbon	1.85
H.O.water	hydrogen in water	1.50
H.N.ammonia	hydrogen in ammonia	1.50
H.O	hydrogen bound to oxygen	1.50
H.N	hydrogen bound to nitrogen	1.50
H.soft	hydrogen otherwise	1.50
N.1	sp nitrogen	5.00
N.2	sp2 nitrogen	1.85
N.3.ammonia	sp3 nitrogen in ammonia	3.00
N.3.primary	sp3 nitrogen in primary amines	3.00
N.3.secondary	sp3 nitrogen in secondary amines	3.00
N.3.tertiary	sp3 nitrogen in tertiary amines	3.00
N.3.aniline	sp3 nitrogen in pyramidal anilines	3.00
N.ar	aromatic nitrogen with a lone pair	3.00
N.ar.no_lp	aromatic nitrogen without a lone pair	1.85
N.pl3.am	planar amide nitrogen	1.85
N.pl3.aniline	planar aniline nitrogen	1.85
N.pl3.primary	planar primary nitrogen	1.85
N.pl3.secondary	planar secondary nitrogen	1.85
N.pl3.tertiary	planar tertiary nitrogen	1.85
N.pl3.nitro	planar nitrogen in nitro	1.85
O.co2	oxygen in carboxylate	3.00
O.2.noxide	oxygen in N-oxides	3.00
O.2.sulfone	oxygen in sulfones	3.00
O.2.sulfoxide	oxygen in sulfoxides	3.00
O.2.po	oxygen in phosphine oxides	3.00
O.2.carbonyl	sp2 oxygen in ketones esters	3.00

O.2.aldehyde	sp2 oxygen in aldehydes	3.00
O.2.am	sp2 oxygen in amides	3.00
O.2.one_lp	sp2 furan-type oxygen	3.00
O.2.nitro	sp2 oxygen in nitro groups	3.00
O.2.other	sp2 oxygen otherwise	3.00
O.3.water	sp3 oxygen in water	3.00
O.3.alcohol	sp3 oxygen in aliphatic alcohols	3.00
O.3.any	sp3 oxygen otherwise	3.00
P.3	phosphorus	-
Si	silicon	-
Se.3	sp3 selenium	5.00
S.O	sulfur in sulfoxides	5.00
S.O2	sulfur in sulfones	-
S.2.ps	sulfur in phosphine sulfides	5.00
S.2.phene	sp2 sulfur in thiophenes	5.00
S.2	sp2 sulfur otherwise	5.00
S.3	sp3 sulfur	5.00
F	fluorine	5.00
Cl	chlorine	5.00
Br	bromine	5.00
I	iodine	5.00

Table S6 Atom types used to assign AIPs.

2.3 Lone Pair AIP Locations

For oxygen, nitrogen and sulfur atoms (excluding atom types S.3, S.2.phene, N.ar lp, and all N.pl3), lone pair AIPs were located as local minima on the 0.0300 e bohr^{-3} electron density isosurface. Ordinarily, the number of local minima matches the expected number of lone pairs, but there are cases, for example, when an atom is engaged in intramolecular H-bond, where fewer lone pair sites are found. For four atom types (O.2.sulfone, O.2.noxide, O.2.po, S.2.ps), the MEPS has high symmetry with no well-defined minima. These atom types are described by three lone pair sites symmetrically placed on the circle defined with respect to the electrostatic potential minimum on the MEPS (Figure S4).

2.4 Fluorine AIP Locations

Fluorine is represented by a single AIP, which is placed on the 0.0300 e bohr^{-3} electron density isosurface on an extension of the C-F vector (Figure S5). The minimum on the 0300 e bohr^{-3} electron density isosurface patch is used to assign the AIP value. In many perfluoro compounds, a negative electrostatic potential is not found on the 0.0300 e bohr^{-3} electron density isosurface, and in these cases, the AIP is assigned a default value of zero (the same default applies to all lone pair AIPs).

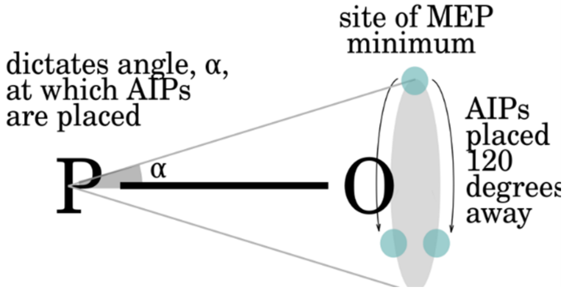

Figure S4 Location of AIPs for lone pairs on O.2.po. The minimum on the MEPS is identified and defines angle α from the P-O vector. A cone described by this vector identifies the sites for other two AIPs, which are positioned symmetrically around the P-O bond.

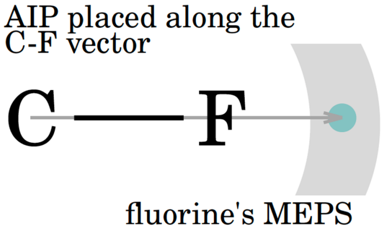

Figure S5 Location of the AIP for fluorine.

2.5 Hydrogen AIP Locations

Hydrogen is represented by a single AIP, which is placed on an extension of the X-H vector (Figure S6). The maximum on the 0.0104 e bohr^{-3} electron density isosurface patch is used to assign the AIP value.

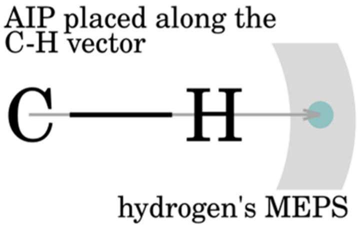

Figure S6 Location of the AIP for a hydrogen in a CH group.

2.6 σ-Hole AIP Locations

For chlorine, bromine, iodine and S.2.ps atoms, the σ-hole AIP is placed on an extension of the bond vector as for hydrogen and fluorine, and the AIP value is assigned using the maximum on

the 0.0104 e bohr^{-3} electron density isosurface. Similarly, for sulfur atoms bonded to two carbon atoms (excluding atom type S.2 which does not have a σ-hole AIP), the two σ-hole AIPs are located using the C-S bond vectors. For the sulfur in a sulfoxide (S.O), the σ-hole AIP was located as the maximum on the 0.0104 e bohr^{-3} electron density isosurface.

2.7 Non-polar AIP Locations

The non-polar regions of the MEPS fall into two distinct categories: discrete patches that represent individual AIP sites, and contiguous surfaces that represent multiple AIPs.

For most atoms with π-system AIPs, the MEPs are non-contiguous with a patch above and below the plane of the ring in which the atom is located. In these cases, the k-medoid algorithm (*22*) from the sklearn library (*23*) was used to both separate the atomic MEPS into two clusters and find the centres. If the ratio of the two cluster areas was smaller than 4 or the distance between the two cluster centres was smaller than 1.5 times the radius of the element, the algorithm was deemed to have failed to correctly locate the two patches. In this case, the procedure was run three times using different initialisation algorithms. If two satisfactory clusters were not identified, the atom was deemed to have a single surface patch. One AIP is located at the centre of each patch, and the value is determined by the minimum in the MEPS of that patch.

For atoms with a contiguous MEPS surface that represents multiple non-polar AIPs (C.1, N.1, S.2.ps, Cl, Br, I), the approach illustrated in Figure S7 was used. The most negative site on the surface patch was found first, and the other three AIPs were arranged geometrically with respect to that site.

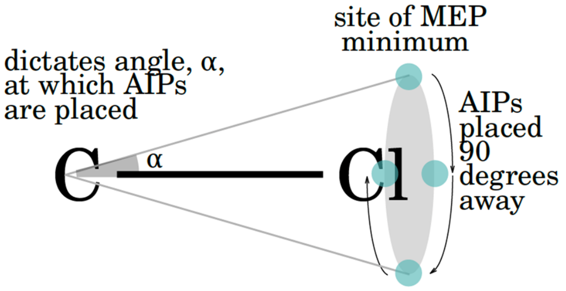

Figure S7 Location of non-polar AIPs on chlorine. The minimum on the MEPS is identified and defines angle α from the C-Cl vector. A cone described by this vector identifies the sites for the other three AIPs, which are positioned symmetrically around the C-Cl bond.

For atoms that have both non-polar π-sites and lone pairs (N.1, N.2, S.2 and all O.2), the lone pair sites were first identified on the 0.0300 e bohr^{-3} electron density isosurface. All points that were within 1.24 Å of these sites were excised from the 0.0020 e bohr^{-3} electron density isosurface (Figure S8), and the remaining surface was used to assign the non-polar AIPs that represent the π-sites as above. However in this case, the maximum in the MEPS was used to assign the AIP value to minimise the through-space effect of the very polar lone pair site.

For non-polar sulfur H-bond acceptors (S.2.phene and S.3), AIPs are located as local minima on the 0.0020 e bohr^{-3} electron density isosurface.

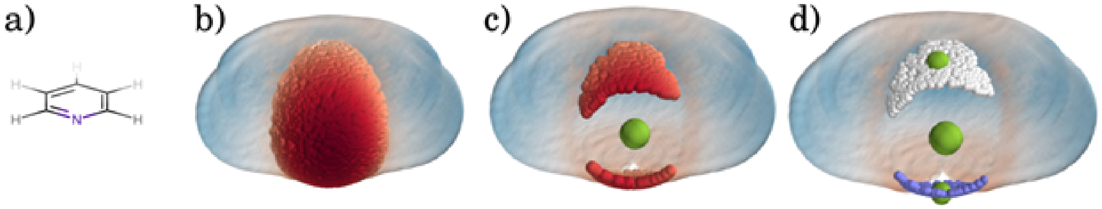

Figure S8 Analysis of the atomic surface of N.ar in pyridine. Excision of the lone pair MEP surface for pyridine in side view, as seen on (a) chemical structure, (b) the 0.0020 e bohr^{-3} electron density isosurface with the patch assigned to nitrogen highlighted by colouring the MEPS value, (c) excision of the all points on the atomic surface within 1.24 Å of the nitrogen lone pair AIP (green), (d) identification of the two π-site patches (blue and white) and assignment of the two AIPs (green).

3 Free Energy of Transfer

3.1 Data

The data used was obtained from refs. (*24*, *25*). The original experimental data was obtained from measurements of the solvation free energies from gas into n-hexadecane and from gas into water obtained from liquid-gas chromatography.(*26*) The solvation free energies of basic or acidic functional groups have been corrected taking into account pKa values. The free energy of transfer from n-hexadecane to water is quoted at the 1M standard state.

Common Name	ΔG°_{expt} / kJ mol^{-1}
methane	6.5
ethane	10.4
butane	17.9
2-methylpropane	17.7
pentane	22
2-methylbutane	21.5
2,2-dimethylpropane	20.9
hexane	25.6
2-methylpentane	24.8
3-methylpentane	25.2
2,2-dimethylbutane	23.9
2,3-dimethylbutane	24.1
heptane	29.3
2-methylhexane	29.4
3-methylhexane	28.7
2,2-dimethylpentane	28
2,3-dimethylpentane	27.8
2,4-dimethylpentane	27.9
3,3-dimethylpentane	27.6
2,2,3-trimethylbutane	27.6
octane	33

3-methylheptane	32.5
2,5-dimethylhexane	30.4
2,2,4-trimethylpentane	29.8
2,3,4-trimethylpentane	30.6
nonane	36.1
2-methyloctane	34.9
2,2,5-trimethylhexane	32.6
decane	40
tricosane	85.4
tetracosane	89
2,2,4,6,6-pentamethylheptane	39.9
2-methyldecane	41.9
cyclopropane	10.6
cyclopentane	19.2
methylcyclopentane	23.3
propylcyclopentane	30.6
pentylcyclopentane	36.9
cyclohexane	22
methylcyclohexane	26.1
cis-1,2-dimethylcyclohexane	28.6
trans-1,2-dimethylcyclohexane	28.7
cis-1,4-dimethylcyclohexane	28.8
trans-1,4-dimethylcyclohexane	28.8
ethylcyclohexane	31.2
propylcyclohexane	32.1
butylcyclohexane	35.7
t-butylcyclohexane	33.5
1,2,4-trimethylcyclohexane	33.0
cyclooctane	28.3
dicyclohexyl	38.3
adamantane	27.4
jp-10	24.5
ethene	7.0
propene	11.0
but-1-ene	14.5
cis-but-2-ene	15.6
trans-but-2-ene	15.1
2-methylprop-1-ene	13.9
pent-1-ene	18.7
cis-pent-2-ene	18.1
trans-pent-2-ene	18.5
3-methylbut-1-ene	18.7
2-methylbut-1-ene	17.9
2-methylbut-2-ene	18.2
hex-1-ene	21.3
cis-hex-2-ene	21.9

2-methylpent-2-ene	21.5
hept-1-ene	24.4
trans-hept-2-ene	25.2
3,3-dimethylpent-1-ene	22.8
oct-1-ene	28.4
trans-oct-2-ene	28.5
2,4,4-trimethylpent-1-ene	27.2
2,4,4-trimethylpent-2-ene	26.2
non-1-ene	31.8
dec-1-ene	35.2
undec-1-ene	38.6
dodec-1-ene	42.2
propadiene	8.8
buta-1,2-diene	11.6
buta-1,3-diene	11.4
2-methylbuta-1,3-diene	14.8
2,3-dimethylbuta-1,3-diene	17.0
penta-1,2-diene	14.9
cis-penta-1,3-diene	14.9
4-methylpenta-1,3-diene	17.2
penta-1,4-diene	15.5
trans-hexa-1,3-diene	18.5
hexa-1,5-diene	18.1
hepta-1,6-diene	22.1
4,8-dimethyl-1,3,7-nonatriene	29.7
cyclopentene	15.3
cyclohexene	18.4
1-methylcyclohexene	22.6
cycloheptene	24
cyclooctene	26.9
cyclopenta-1,3-diene	11.8
dicyclopentadiene	19.3
cyclohexa-1,3-diene	15.2
cyclohexa-1,4-diene	13.9
cyclohepta-1,3,5-triene	15.5
methylenecyclopentane	18.1
methylenecyclohexane	21.6
ethyne	0.4
propyne	3.9
but-1-yne	8
pent-1-yne	11.5
hex-1-yne	15.5
hept-1-yne	19.6
oct-1-yne	23.0
but-1-en-3-yne	8.6
fluoroethane	6.0

1-fluoropropane	8.5
2-fluoropropane	8.6
1-fluorobutane	11.8
1-fluoropentane	15.1
1,1-difluoroethane	2.7
1,1,1-trifluoroethane	6.7
1,1,2-trifluoroethane	-0.4
1,1,1,2-tetrafluoroethane	5.4
1,1,2,2-tetrafluoroethane	2.5
pentafluoroethane	6.6
1,1,1-trifluoropropane	9.6
1,1,1-trifluorobutane	13.6
1,1,1-trifluoropentane	17.1
1,1,1-trifluorohexane	20.5
1,1,1-trifluoroheptane	23.8
1,1,1-trifluorooctane	27.2
1,1,1-trifluorononane	30.6
1,1,1-trifluorodecane	34
1,1,1-trifluoroundecane	37.4
1,1,1-trifluorododecane	40.8
difluoromethane	-1.1
trifluoromethane	1.4
tetrafluoromethane	8.5
octafluorocyclobutane	18.6
perfluorodimethylcyclobutane	27.5
1,1-difluoroethene	8.5
chloromethane	4.3
dichloromethane	6
trichloromethane	9.6
tetrachloromethane	16.4
chloroethane	7.0
1,1-dichloroethane	9.7
1,2-dichloroethane	7.2
1,1,1-trichloroethane	14.8
1,1,2-trichloroethane	10.4
1,1,2,2-tetrachloroethane	11.4
pentachloroethane	18.5
1-chloropropane	11.2
2-chloropropane	10.2
1,2-dichloropropane	10.9
1,3-dichloropropane	9.8
1,2,3-trichloropropane	10.3
1-chlorobutane	14.8
2-chlorobutane	14.5
2-chloro-2-methylpropane	17.5
1,4-dichlorobutane	10.3

1-chloropentane	18.1
1-chlorohexane	21.2
1-chloroheptane	25.2
alpha-hexachlorocyclohexane	20.1
beta-hexachlorocyclohexane	19.3
gamma-hexachlorocyclohexane	19.2
delta-hexachlorocyclohexane	20.7
endrin	23.6
a-endosulfan	28.6
b-endosulfan	26.1
perfluoropropene	12
1,1-dichloroethene	13.1
cis-1,2-dichloroethene	9
trans-1,2,-dichloroethene	9.8
trichloroethene	15.3
tetrachloroethene	20.8
3-chloroprop-1-ene	9.6
2-chlorobuta-1,3-diene	12.7
hexachlorobuta-1,3-diene	28.3
3-chlorobut-1-ene	11.4
4-chlorobut-1-ene	11.5
1-chloro-2-methylprop-1-ene	14.4
3-chloro-2-methylprop-1-ene	10.2
5-chloropent-1-yne	7.1
bromomethane	5.9
dibromomethane	8.3
tribromomethane	12.7
bromoethane	9
1,2-dibromoethane	9.5
1,1,2,2-tetrabromoethane	16.8
1-bromopropane	12.6
2-bromopropane	11.6
1-bromo-2-methylpropane	16.8
2-bromo-2-methylpropane	18.4
1-bromopentane	20.2
1-bromohexane	24.3
1-bromoheptane	28.0
1-bromooctane	31.5
1-bromoadamantane	25.0
bromoethene	9.2
iodomethane	8.3
diiodomethane	12
iodoethane	11.6
1-iodopropane	15.6
1-iodobutane	19.7
1-iodopentane	23

1-iodohexane	26.7
1-iodoheptane	30.2
fluorochloromethane	2.4
chlorobromomethane	7.1
fluorochlorobromomethane	7.7
fluorodichloromethane	6.6
fluorodibromomethane	6.5
fluorotrichloromethane	13.7
difluorodichloromethane	12.8
trifluorochloromethane	10.7
1,1-difluoro-1-chloroethane	8.7
1-fluoro-1,1-dichloroethane	11.8
1,1,2-trifluoro-1-chloroethane	1.0
1,1,1-trifluoro-2,2-dichloroethane	9.9
1,1,1-trifluoro-2-chloro-2-bromoethane	10.6
1,1,1,2-tetrafluoro-2-bromoethane	10.4
1,1,1,2-tetrafluoro-2-chloroethane	8.6
1,1-difluoro-1,2,2-trichloroethane	15.4
1,1,2-trifluoro-1-bromo-2-chloroethane	13.6
1,1,2-trifluorotrichloroethane	20.0
1,2-difluorotetrachloroethane	20.9
1,2-dichlorotetrafluoroethane	17.6
chloropentafluoroethane	15.2
1-bromo-3-chloropropane	11.2
1,2,2,3-tetrafluoro-1-chloro-3-bromopropane	11.9
1,2,2,3-tetrafluoro-1-chloro-1-bromopropane	14.2
1,2,2,3-tetrafluoro-1,3-dichloropropane	9.4
1,2,2,3-tetrafluoro-1,1-dichloropropane	13.5
1,1,2,2,3-pentafluoro-3,3-dibromopropane	14.8
2-bromoheptafluoropropane	20.9
2-chloroheptafluoropropane	16.3
1,2,3-trichloropentafluoropropane	23.3
2,3-dichlorooctafluorobutane	26.2
1,2,3,4-tetrachlorohexafluorobutane	29.7
1-bromo-1,1,2,2,3,3,4,4,5,5,6,6-dodecafluorohexane	30.2
1,4-dibromooctafluorobutane	28.1
1,1,-difluoro-2-chloroethene	6.3
cis-1,2-dichlorohexafluorocyclobutane	22.2
trans-1,2-dichlorohexafluorocyclobutane	22.1
racemic-1,2-dichlorohexafluorocyclobutane	22.5
dimethylether	-0.6
diethylether	4.2
dipropylether	11.7
diisopropylether	8
dibutylether	18.9
methylethylether	0.3

methyl-tert-butylether	5.2
methyl-tert-pentylether	8.5
ethyl-tert-butylether	8.4
ethyl-t-pentylether	9.8
isopropyl-tert-butylether	12.6
methylcyclohexylether	8.2
ethylvinylether	3.5
dimethoxymethane	-1.7
1,1-diethoxypropane	4.2
1,1-dimethoxybutane	0.7
1,1-dibutoxybutane	21.2
2,2-dimethoxypropane	1.3
2,2-diethoxypropane	1.9
1,2-dimethoxyethane	-5.1
1-butoxy-2-methoxypropane	5.6
ethylene oxide	-5.3
1,2-butylene oxide	1.9
1,2-epoxybut-3-ene	0.3
cyanoethylene oxide	-14.4
1,2:3,4-diepoxybutane	-9.8
bis(2-chloroethyl) ether	3.7
bis(2-chloroisopropyl)ether	6.6
1-bromo-2,3-epoxypropane	0.0
cf3ch2ocf3	12.8
1,1,1,2-tetrafluoro-2-chlorofluoromethoxyethane	10.6
cf3ch2ocf2cl	16.1
chcl2cf2och3	11.6
cf3chclocf2h	11.1
chfclcf2ocf2h	9.6
(cf3)2ch-o-ch2f	8.8
cf3ch2och=ch2	5.7
bis(2,2,2-trifluoroethyl)ether	7.4
(cf3)2choch3	11.5
chf2ochfcf3	8.3
chf2cf2chfochf2	7.4
tetrahydrofuran	0.5
2-methyltetrahydrofuran	2.3
2,5-dimethyltetrahydrofuran	4.8
2,5-dimethoxytetrahydrofuran	-6.3
1,3-dioxane	-7.5
tetrahydropyran	4.1
1,4-dioxane	-4.6
formaldehyde	-7.4
acetaldehyde	-7.6
propionaldehyde	-4
butyraldehyde	-0.3

2-methylproprionaldehyde	0.1
pentanal	3.6
hexanal	7.4
2-methylpentanal	8.4
heptanal	10.9
octanal	15.3
nonanal	18.9
decanal	22.3
undecanal	26.1
dodecanal	30.0
tridecanal	33.5
tetradecanal	37.3
glutaraldehyde	-7.4
2-(4-ethylcyclohexyl)ethanal	18.7
propenal	-4.2
trans-but-2-en-1-al	-3.3
trans-pent-2-en-1-al	3
trans-2-methylbut-2-en-1-al	1.4
3-methylbut-2-en-1-al	2.6
trans-hex-2-en-1-al	6.4
cis-hex-3-en-1-al	6.7
2-methylpent-2-en-1-al	4.9
trans-hept-2-en-1-al	10.0
trans-oct-2-en-1-al	13.6
oct-7-en-1-al	10.4
trans-non-2-en-1-al	17.0
trans-dec-2-en-1-al	20.6
trans-undec-2-en-1-al	24.1
e,e-nona-2,4-dienal	12.8
e,z-nona-2,6-dienal	13.2
e,e-deca-2,4-dienal	16
chloral hydrate	-10
propanone	-6.4
butanone	-2.5
pentan-2-one	1.0
pentan-3-one	1.8
3-methylbutan-2-one	1.8
hexan-2-one	5
hexan-3-one	4.9
4-methylpentan-2-one	4.8
3,3-dimethylbutan-2-one	3.7
heptan-2-one	8.7
heptan-4-one	8.3
5-methylhexan-2-one	7.3
2,4-dimethylpentan-3-one	7.9
octan-2-one	12.3

3-methylheptan-4-one	11.6
octan-4-one	11.6
nonan-2-one	16.6
nonan-5-one	15.7
2,2,4,4-tetramethylpentan-3-one	14.7
decan-2-one	20.1
undecan-2-one	23.7
cyclopropylmethylketone	-1.4
dicyclopropylketone	1.1
cyclohexylmethylketone	7.6
cyclopentanone	-1.3
2-methylcyclopentanone	2.1
3-ethylcyclopentanone	5.1
cyclohexanone	1.1
2-methylcyclohexanone	5.6
cycloheptanone	5.5
cyclooctanone	9.1
pent-1-en-3-one	-1.1
pent-3-en-2-one	-1.7
non-8-en-2-one	11.6
6-methylhepta-3,5-diene-2-one	4.5
isophorone	9.0
1-methylcyclopent-1-en-3-one	-2.2
2-methylcyclopent-2-en-1-one	-1.4
cyclopenteneone	-5.2
butan-2,3-dione	-6
pentan-2,3-dione	-2.5
pentane-2,4-dione	-1.2
hexane-2,5-dione	-9.9
nonane-4,6-dione	11.8
cyclohexan-1,4-dione	-7.4
cyclohexan-1,3-dione	-4.8
1,1,1-trifluoropentan-2,4-dione	-4.1
thenoyl trifluoroacetone	3.3
diethyl carbonate	4.3
propylene carbonate	-10.7
ascorbic acid	-30.6
g-butyrolactone	-9.6
d-pentanolactone	-10.1
methyl formate	-4.3
ethyl formate	-0.6
propyl formate	3.5
isopropyl formate	4.3
butyl formate	6.7
isobutyl formate	6.6
pentyl formate	10.3

isopentyl formate	10
methyl acetate	-2.2
ethyl acetate	0.9
propyl acetate	4.4
isopropyl acetate	3.5
butyl acetate	8.0
isobutyl acetate	8.2
pentyl acetate	11.4
isopentyl acetate	12.1
hexyl acetate	15
heptyl acetate	18.7
vinyl acetate	0.9
methyl propanoate	1.6
ethyl propanoate	4.8
propyl propanoate	8.8
isopropyl propanoate	7.2
butyl propanoate	11.7
isobutyl propanoate	11
sec-butyl propanoate	10.7
pentyl propanoate	15.9
methyl butanoate	4.6
ethyl butanoate	8.2
propyl butanoate	12
methyl pentanoate	8.6
ethyl pentanoate	11.1
butyl pentanoate	19.2
methyl hexanoate	11.6
ethyl hexanoate	14.9
ethyl isobutanoate	7.3
isobutyl isobutanoate	15.1
methyl trimethylacetate	6.7
methyl cyclopropanecarboxylate	0.2
methyl cyclohexanecarboxylate	11.1
glycerol triacetate	-9.0
acetol acetate	-7.9
ethyl acetoacetate	-3.9
ethylidene diacetate	-2.3
ethyl trifluoroacetate	4.8
ethyl trichloroacetate	13.6
dibutyl succinate	16.9
diethyl tartrate	-18.3
triethyl citrate	-10.7
acetonitrile	-6.3
proprionitrile	-4.2
1-cyanopropane	-0.7
1-cyanobutane	3.0

methacrylonitrile	1.8
allyl cyanide	-1.2
1,2-dicyanoethane	-13.7
1,3-dicyanopropane	-11.1
1,4-dicyanobutane	-9.7
methylamine	-11.6
ethylamine	-9.2
propylamine	-6.2
butylamine	-2.8
pentylamine	0.8
hexylamine	4.3
heptylamine	7.8
octylamine	11.0
cyclohexylamine	2.5
dimethylamine	-8.8
diethylamine	-3.4
dipropylamine	3.8
diisopropylamine	3.0
dibutylamine	11.2
methylethylamine	-3.9
trimethylamine	-4.2
triethylamine	3.9
methyldiethylamine	3.1
dimethylisopropylamine	1.9
cyanamide	-15.2
2-methoxyethylamine	-13.2
2-ethoxyethylamine	-9.8
3-methoxypropylamine	-11.6
1,2-diaminocyclohexane	-19.7
1,3-diaminocyclohexane	-19.7
1,4-diaminocyclohexane	-19.7
guanidine	-25.1
4-aminobutylguanidine	-33.4
diethyldiimide	5.6
nitromethane	-6
nitroethane	-1.8
1-nitropropane	2.5
2-nitropropane	1.4
1-nitrobutane	6.6
1-nitropentane	10.7
formamide	-28.6
acetamide	-26.0
proprionamide	-20.1
butanamide	-18.5
pentanamide	-14.8
n-methylformamide	-21.2

n-methylacetamide	-22.3
n-methylpropanamide	-18.2
n-ethylacetamide	-16.4
n-butylacetamide	-11.4
n,n-dimethylformamide	-14.6
n,n-dibutylformamide	7.3
n,n-dimethylacetamide	-13.7
succinamide	-42.3
chloralformamide	-15.8
iodoacetamide	-18.5
methylurea	-28.6
1,1-dimethylurea	-24.9
1,3-dimethylurea	-18.6
trimethylurea	-14.7
tetramethylurea	-5.6
ethylurea	-24.1
1,1-diethylurea	-16.5
1,3-diethylurea	-11.6
triethylurea	-4.4
tetraethylurea	6.1
propylurea	-21.0
butylurea	-16.4
adalin	-11.4
methylacetylurea	-21.1
metformin	-30.1
ethyl carbamate	-13.2
methyl urethan	-7.5
ethyl urethan	-4.2
propyl urethan	-0.7
isopropyl urethan	-2.4
butyl urethan	2.7
isobutyl urethan	1.7
isopentyl urethan	5
2-ethyl-2-methylsuccinimide	-15.2
1,6-hexanediisocyanate	16
hexyl isocyanate	18.3
formic acid	-21.6
acetic acid	-17.5
propanoic acid	-14
butanoic acid	-10.7
2-methylpropanoic acid	-10.4
pentanoic acid	-7
2-methylbutanoic acid	-6.8
3-methylbutanoic acid	-7.2
2,2-dimethylpropanoic acid	-6.7
hexanoic acid	-3.5

2-methylpentanoic acid	-3.2
3-methylpentanoic acid	-3.7
2,2-dimethylbutanoic acid	-3.4
heptanoic acid	-0.1
octanoic acid	3.0
nonanoic acid	6.7
decanoic acid	10.4
undecanoic acid	13.7
dodecanoic acid	17
tridecanoic acid	20.6
tetradecanoic acid	24.1
pentadecanoic acid	27.8
hexadecanoic acid	31.3
heptadecanoic acid	34.9
octadecanoic acid	38.1
nonadecanoic acid	41.6
eicosanoic acid	45.1
heneicosanoic acid	48.6
docosanoic acid	52.1
tricosanoic acid	55.6
tetracosanoic acid	59.0
2-isopropyl-3-methylbutanoic acid	2.3
propenoic acid	-13.0
trans-2-butenoic acid	-10.8
2-methylpropenoic acid	-9.1
methyl 2-cyano-2-propenoic acid	-0.9
ethyl 2-cyano-2-propenoic acid	2.1
isopropyl 2-cyano-2-propenoic acid	4.6
2-methoxyethyl 2-cyano-2-propenoic acid	-3.4
sorbic acid	-7.6
linoleic acid	29.5
fluoroacetic acid	-22.0
difluoroacetic acid	-20.7
trifluoroacetic acid	-19.5
chloroacetic acid	-20.3
dichloroacetic acid	-17.1
trichloroacetic acid	-12.3
bromoacetic acid	-18.8
dibromoacetic acid	-13.3
tribromoacetic acid	-7.7
iodoacetic acid	-15.2
diiodoacetic acid	-8.7
triiodoacetic acid	-1.5
methoxyacetic acid	-17.7
ethoxyacetic acid	-15.6
2-hydroxybutanoic acid	-12.7

tretinoin	23.8
pyruvic acid	-20.8
acetylglycine	-34.5
acetic anhydride	-7.9
malonic acid	-30.7
succinic acid	-30.1
glutaric acid	-29.2
adipic acid	-28.4
pimelic acid	-24.9
suberic acid	-22.8
citric acid	-22.5
malic acid	-37.8
tartaric acid	-38.8
dimethylmalonic acid	-23.8
cyclopropanecarboxylic acid	-10.3
cyclobutanecarboxylic acid	-7.6
cyclopentanecarboxylic acid	-4.8
cyclohexanecarboxylic acid	-1.9
chlorogenic acid	-65
beta-carboxyadipic acid	-45.7
aconitic acid	-39.7
fumaric acid	-19.3
maleic acid	-24.1
diglycolic acid	-34.4
lactic acid	-23.8
peracetic acid	-17.7
water	-25.3
methanol	-15.8
ethanol	-12.4
propan-1-ol	-8.7
propan-2-ol	-9.8
butan-1-ol	-4.9
2-methylpropan-1-ol	-5.1
butan-2-ol	-6
2-methylpropan-2-ol	-7.5
pentan-1-ol	-1.4
pentan-2-ol	-2.2
pentan-3-ol	-1.9
2-methylbutan-1-ol	-1.3
3-methylbutan-1-ol	-1.3
2-methylbutan-2-ol	-3.4
3-methylbutan-2-ol	-2.1
hexan-1-ol	2.2
hexan-3-ol	2.1
2-methylpentan-1-ol	1.7
3-methylpentan-1-ol	1.5

4-methylpentan-1-ol	2.1
2-methylpentan-2-ol	-0.1
3-methylpentan-2-ol	1.3
4-methylpentan-2-ol	1.7
2-methylpentan-3-ol	-0.3
3,3-dimethylbutan-1-ol	-0.3
2,3-dimethylbutan-2-ol	-0.2
heptan-1-ol	5.9
2-methylhexan-2-ol	3.6
3-methylhexan-2-ol	5.5
2-methylhexan-3-ol	3.4
3-methylhexan-3-ol	3.6
3-ethylpentan-3-ol	3.8
2,2-dimethylpentan-3-ol	6.5
2,4-dimethylpentan-3-ol	3.5
octan-1-ol	9.2
nonan-1-ol	13
2,6-dimethylheptan-4-ol	8.7
decan-1-ol	16.8
undecan-1-ol	20.7
dodecan-1-ol	23.8
tridecan-1-ol	27.2
tetradecan-1-ol	30.7
pentadecan-1-ol	34.3
hexadecan-1-ol	37.8
heptadecan-1-ol	41.4
octadecan-1-ol	45
nonadecan-1-ol	48.5
eicosan-1-ol	52.1
cyclopentanol	-4.5
cyclohexanol	-1.4
cycloheptanol	2.2
cyclooctanol	7.1
adamantan-1-ol	2.7
prop-2-yn-1-ol	-14.1
but-2-yn-1-ol	-10.4
but-3-yn-1-ol	-13
pent-2-yn-1-ol	-6.8
pent-3-yn-1-ol	-9.1
hex-2-yn-1-ol	-3.0
hex-5-yn-1-ol	-6.0
hept-2-yn-1-ol	0.4
oct-2-yn-1-ol	3.8
non-2-yn-1-ol	7.3
prop-2-en-1-ol	-9.9
pent-1-en-3-ol	-5.6

hex-1-en-3-ol	-2.3
cis-hex-3-en-1-ol	-0.7
trans-hex-3-en-1-ol	-2.2
hept-1-en-3-ol	1.0
oct-1-en-3-ol	4.4
trans-oct-2-en-1-ol	6.4
cyclohex-2-en-1-ol	-5.4
4-hydroxy-2-nonenal	-2.2
2,2,2-trifluoroethanol	-11.1
1,1,1-trifluoropropan-2-ol	-5.4
2,2,3,3-tetrafluoropropan-1-ol	-8.1
2,2,3,3,3-pentafluoropropan-1-ol	-3.3
1,1,1,3,3,3-hexafluoropropan-2-ol	-7.8
1,1,1,3,3,3-hexafluoro-2-methylpropan-2-ol	1.7
2,2,3,4,4,4-hexafluorobutan-1-ol	-3.4
2,2,3,3,4,4,4-heptafluorobutan-1-ol	1.1
3,3,4,4,5,5,5-heptafluoropentan-2-ol	5.1
2,2,3,3,4,4,5,5-octafluoropentan-1-ol	0.0
2,2,3,3,4,4,5,5,6,6,6-undecafluorohexan-1-ol	11.2
2,2,3,3,4,4,5,5,6,6,7,7-dodecafluoroheptan-1-ol	7.6
2,2,3,3,4,4,5,5,6,6,7,7,7-tridecafluoroheptan-1-ol	16.5
3,3,4,4,5,5,6,6,7,7,8,8-tridecafluorooctan-1-ol	4.2
2,2,3,3,4,4,5,5,6,6,7,7,8,8,8-pentadecafluorooctan-1-ol	20.1
2-chloroethanol	-12.3
2,2,2-trichloroethanol	-4.1
3-chloropropan-1-ol	-9.5
1,3-dichloropropan-2-ol	-8.4
cyclopropanemethanol	-9.0
cyclobutanemethanol	-5.3
cyclopentanemethanol	-1.6
cyclohexanemethanol	2.0
cycloheptanemethanol	5.6
cyclooctanemethanol	9.2
cyclononanemethanol	13
cyclodecanemethanol	16.6
cycloundecanemethanol	20.3
cyclododecanemethanol	23.9
cyclotridecanemethanol	27.6
cyclotetradecanemethanol	31.3
2-methoxyethanol	-14.8
2-ethoxyethanol	-12.5
2-propoxyethanol	-7.9
2-butoxyethanol	-4.9
2-isopropoxyethanol	-9
2-isobutoxyethanol	-6.8
1-methoxypropan-2-ol	-13.6

ethan-1,2-diol	-25.3
propan-1,2-diol	-23.5
propan-1,3-diol	-29.5
butan-1,2-diol	-21.3
pentan-1,2-diol	-17.9
pentan-2,4-diol	-24.1
pentan-1,5-diol	-23.5
hexan-1,2-diol	-14.5
hexan-1,5-diol	-20.4
hexan-1,6-diol	-20.6
heptan-1,2-diol	-11.1
heptan-1,7-diol	-18.0
octan-1,2-diol	-7.7
octan-1,8-diol	-14.8
trans-cyclohexane-1,2-diol	-20.7
cis-cyclohexane-1,2-diol	-16.7
trans-cyclooctane-1,2-diol	-13.6
cis-cyclooctane-1,2-diol	-9.4
erythritol	-46.2
mannitol	-51.4
3-hydroxybutan-2-one	-12.4
4-hydroxy-4-methylpentan-2-one	-8.1
hydroxypropanone	-16.1
hex-3-yne-2,5-diol	-26.4
2,2-dimethylpropan-1,3-diol	-22.2
3-methylbutan-1,3-diol	-27.9
cyanomethanol	-21.8
ethyl lactate	-6.4
methyl lactate	-9.1
chloralose	-18.7
3-chloropropan-1,2-diol	-18.8
glycolamide	-30.2
lactamide	-28.6
ethyl glycollate	-12.7
2-amino-2-methylpropan-1-ol	-20.7
2-aminoethanol	-24.9
3-aminopropan-1-ol	-21.5
4-aminobutan-1-ol	-22.4
5-aminopentan-1-ol	-20.1
6-aminohexan-1-ol	-16.7
n-methyldiethanolamine	-23
n-methylethanolamine	-19.9
n-ethylethanolamine	-16.1
n,n-dimethylethanolamine	-13.3
n,n-diethylethanolamine	-6.8
1,2,3,4,5-pentahydroxypentane	-34.5

but-2-en-1,4-diol	-22.3
but-3-en-1,2-diol	-22.8
1,2-epoxybut-3,4-diol	-31.6
tetrahydrothiophene	8
bis(2-chloroethyl)sulfide	11.1
dimethylsulfoxide	-22.5
tetramethylenesulfone	-12.6
sulfonal	-1.3
allicin	2.5
sulfur hexafluoride	12
carbon disulphide	14.4
thiourea	-29.6
allylthiourea	0.0
allyl isothiocyanate	10.0
methional	-2.7
sulfosalicylic acid	-35.5
tributylphosphine oxide	-3.5
trihexylphosphine oxide	8.6
trioctylphosphine oxide	22.4
1,2,2-trimethylpropylmethylphosphonofluoridate	4.1
diethyl phosphate	-28.1
dibutyl phosphate	-10.7
dioctyl phosphate	18.3
trimethyl phosphate	-13.5
triethyl phosphate	-4.5
tripropyl phosphate	4.3
tributyl phosphate	13.9
dimethyl methanephosphonate	-8
diphenylphosphoric acid	-20
dibenzylphosphoric acid	-12.4
bis(4-chlorophenyl)phosphoric acid	-8.9
dibutylphosphorodithiotic acid	12.4
dimethyl dichlorovinylphosphonate	1.7
trichlorfon	-14.0
trimethylborate	-2.2
benzene	12.3
toluene	15.3
ethylbenzene	18.3
o-xylene	18.7
m-xylene	18.4
p-xylene	18.5
propylbenzene	21.9
isopropylbenzene	22
1,2,3-trimethylbenzene	21.0
1,2,4-trimethylbenzene	21.7
1,3,5-trimethylbenzene	21.0

2-ethyltoluene	20.5
4-ethyltoluene	20.5
butylbenzene	25.3
isobutylbenzene	26.4
sec-butylbenzene	23.8
tert-butylbenzene	23.3
1,2,4,5-tetramethylbenzene	25.7
4-isopropyltoluene	23.3
pentylbenzene	28.9
hexylbenzene	32.5
styrene	16.8
a-methylstyrene	19.3
3-methylstyrene	18.6
4-methylstyrene	18.7
allylbenzene	17.3
trans-stilbene	27.1
biphenyl	23.2
naphthalene	19.4
1-methylnaphthalene	21.8
2-methylnaphthalene	22.1
1,2-dimethylnaphthalene	24.6
1,3-dimethylnaphthalene	25.3
1,4-dimethylnaphthalene	25.6
1,5-dimethylnaphthalene	25.7
1,6-dimethylnaphthalene	25.8
1,7-dimethylnaphthalene	25
1,8-dimethylnaphthalene	25.2
2,3-dimethylnaphthalene	24.8
2,6-dimethylnaphthalene	25.7
2,7-dimethylnaphthalene	25.7
1,4,5-trimethylnaphthalene	29.7
1,4,6-trimethylnaphthalene	28.7
2,3,5-trimethylnaphthalene	28.1
2,3,6-trimethylnaphthalene	28.2
indane	20.1
acenaphthene	23.7
fluorene	25.5
anthracene	25.9
phenanthrene	27.6
fluoranthene	30.8
pyrene	30.4
coronene	37.4
fluorobenzene	12.6
1,2-difluorobenzene	13.5
1,2,3-trifluorobenzene	14.5
1,2,4-trifluorobenzene	13.8

1,3,5-trifluorobenzene	16.1
4-fluorotoluene	15.7
1,3,5-tri(trifluoromethyl)benzene	29.6
perfluorotoluene	21.3
2,3,4,5,6-pentafluorotoluene	19.4
benzotrifluoride	15.5
2-fluorobiphenyl	24.4
chlorobenzene	16.2
1,2-dichlorobenzene	20.1
1,3-dichlorobenzene	21.1
1,4-dichlorobenzene	21.1
1,2,3-trichlorobenzene	25.7
1,2,4-trichlorobenzene	25.3
1,3,5-trichlorobenzene	25.6
1,2,3,4-tetrachlorobenzene	29.6
1,2,3,5-tetrachlorobenzene	27
1,2,4,5-tetrachlorobenzene	28.2
pentachlorobenzene	30.9
hexachlorobenzene	33.6
2-chlorotoluene	19.0
2,4,5-trichlorotoluene	28.8
3,4,5-trichlorotoluene	27.7
2-chlorobiphenyl	27.3
3-chlorobiphenyl	28.2
4-chlorobiphenyl	28.1
2,2'-dichlorobiphenyl	29.5
2,3-dichlorobiphenyl	30.3
2,3'-dichlorobiphenyl	30.4
2,4-dichlorobiphenyl	30.2
2,4'-dichlorobiphenyl	30.3
2,5-dichlorobiphenyl	30.3
3,3'-dichlorobiphenyl	31.2
3,4-dichlorobiphenyl	31.1
3,4'-dichlorobiphenyl	31.2
3,5-dichlorobiphenyl	30.7
4,4'-dichlorobiphenyl	31.1
2,2',3-trichlorobiphenyl	32.7
2,2',4-trichlorobiphenyl	32.7
2,2',5-trichlorobiphenyl	32.7
2,2',6-trichlorobiphenyl	32.6
2,3,3'-trichlorobiphenyl	33.6
2,3,4-trichlorobiphenyl	33.6
2,3,5-trichlorobiphenyl	33.6
2,3,6-trichlorobiphenyl	32.7
2,3',4-trichlorobiphenyl	33.5
2,3',5-trichlorobiphenyl	33.6

2,3’,6-trichlorobiphenyl	32.8
2,4,4’-trichlorobiphenyl	33.5
2,4,5-trichlorobiphenyl	33.5
2,4,6-trichlorobiphenyl	32.1
2,4’,5-trichlorobiphenyl	33.5
2,4’6-trichlorobiphenyl	32.7
2’,3,4-trichlorobiphenyl	33.5
2’,3,5-trichlorobiphenyl	33.6
3,3’,4-trichlorobiphenyl	34.4
3,3’,5-trichlorobiphenyl	34.4
3,4,4’-trichlorobiphenyl	34.4
3,4,5-trichlorobiphenyl	34.4
3,4’5-trichlorobiphenyl	33.9
2,2’,3,3’tetrachlorobiphenyl	35.8
2,2’,3,4-tetrachlorobiphenyl	35.8
2,2’,3,4’-tetrachlorobiphenyl	35.7
2,2’,3,5-tetrachlorobiphenyl	35.8
2,2’3,5’-tetrachlorobiphenyl	35.8
2,2’,3,6-tetrachlorobiphenyl	35.7
2,2’,3,6’-tetrachlorobiphenyl	35.7
2,2’,4,4’-tetrachlorobiphenyl	35.7
2,2’,4,5-tetrachlorobiphenyl	35.7
2,2’,4,5’-tetrachlorobiphenyl	35.8
2,2’,4,6-tetrachlorobiphenyl	35.1
2,2’,4,6’-tetrachlorobiphenyl	35.6
2,2’,5,5’-tetrachlorobiphenyl	35.8
2,2’,5,6’-tetrachlorobiphenyl	35.7
2,2’,6,6’-tetrachlorobiphenyl	35.5
2,3,3’,4-tetrachlorobiphenyl	36.6
2,3,3’,4’-tetrachlorobiphenyl	36.6
2,3,3’,5-tetrachlorobiphenyl	36.6
2,3,3’,5’-tetrachlorobiphenyl	36.6
2,3,3’,6-tetrachlorobiphenyl	35.8
2,3,4,4’-tetrachlorobiphenyl	36.6
2,3,4,5-tetrachlorobiphenyl	36.6
2,3,4,6-tetrachlorobiphenyl	35.8
2,3,4’,5-tetrachlorobiphenyl	36.6
2,3,4’,6-tetrachlorobiphenyl	35.8
2,3,5,6-tetrachlorobiphenyl	35.4
2,3’,4,4’-tetrachlorobiphenyl	36.6
2,3’,4,5’-tetrachlorobiphenyl	36.6
2,3’,4,6-tetrachlorobiphenyl	35.8
2,3’,4’,5-tetrachlorobiphenyl	36.5
2,3’,4’,6-tetrachlorobiphenyl	35.8
2,3’,5,5’-tetrachlorobiphenyl	36.6
2,3’5’6-tetrachlorobiphenyl	35.8

2,4,4',6-tetrachlorobiphenyl	35.7
2',3,4,5-tetrachlorobiphenyl	36.6
3,3'4,4'-tetrachlorobiphenyl	37.4
3,3',4,5-tetrachlorobiphenyl	37.4
3,3',4,5'-tetrachlorobiphenyl	37.4
3,3',5,5'-tetrachlorobiphenyl	37.1
3,4,4',5-tetrachlorobiphenyl	37.4
2,2',3,3',4-pentachlorobiphenyl	38.8
2,2',3,3',5-pentachlorobiphenyl	38.8
2,2',3,3',6-pentachlorobiphenyl	38.7
2,2',3,4,4'-pentachlorobiphenyl	38.7
2,2',3,4,5-pentachlorobiphenyl	38.8
2,2',3,4,5'-pentachlorobiphenyl	38.7
2,2',3,4,6-pentachlorobiphenyl	38.7
2,2',3,4,6'-pentachlorobiphenyl	38.7
2,2',3,4',5-pentachlorobiphenyl	38.8
2,2',3,4',6-pentachlorobiphenyl	38.6
2,2',3,5,5'-pentachlorobiphenyl	38.8
2,2',3,5,6-pentachlorobiphenyl	38.7
2,2',3,5,6'-pentachlorobiphenyl	38.7
2,2',3,5',6-pentachlorobiphenyl	38.7
2,2',3,6,6'-pentachlorobiphenyl	38.6
2,2',3',4,5-pentachlorobiphenyl	38.7
2,2',3',4,6-pentachlorobiphenyl	38.7
2,2',4,4',5-pentachlorobiphenyl	38.7
2,2',4,4',6-pentachlorobiphenyl	38.6
2,2',4,5,5'-pentachlorobiphenyl	38.8
2,2',4,5,6'-pentachlorobiphenyl	38.6
2,2',4,5',6-pentachlorobiphenyl	38.7
2,2',4,6,6'-pentachlorobiphenyl	38.5
2,3,3',4,4'-pentachlorobiphenyl	39.5
2,3,3',4,5-pentachlorobiphenyl	39.7
2,3,3',4',5-pentachlorobiphenyl	39.6
2,3,3',4,5'-pentachlorobiphenyl	39.5
2,3,3',4',6-pentachlorobiphenyl	38.7
2,3,3',5,5'-pentachlorobiphenyl	39.7
2,3,3',5,6-pentachlorobiphenyl	38.8
2,3,3',5',6-pentachlorobiphenyl	38.8
2,3,4,4',5-pentachlorobiphenyl	39.6
2,3,4,4',6-pentachlorobiphenyl	38.7
2,3,4,5,6-pentachlorobiphenyl	38.8
2,3,4',5,6-pentachlorobiphenyl	37.8
2,3',4,4',5-pentachlorobiphenyl	39.6
2,3',4,4',6-pentachlorobiphenyl	38.7
2,3',4,5,5'-pentachlorobiphenyl	39.6
2,3',4,5',6-pentachlorobiphenyl	38.7

2',3,3',4,5-pentachlorobiphenyl	39.6
2',3,4,4',5-pentachlorobiphenyl	39.5
2',3,4,5,5'-pentachlorobiphenyl	39.7
2',3,4,5,6'-pentachlorobiphenyl	38.7
3,3',4,4',5-pentachlorobiphenyl	40.5
3,3',4,5,5'-pentachlorobiphenyl	40.5
2,2',3,3',4,4'-hexachlorobiphenyl	41.8
2,2',3,3',4,5-hexachlorobiphenyl	41.8
2,2',3,3',4,5'-hexachlorobiphenyl	41.8
2,2',3,3,',4,6-hexachlorobiphenyl	41.6
2,2',3,3',4,6'-hexachlorobiphenyl	41.7
2,2',3,3',5,5'-hexachlorobiphenyl	41.8
2,2',3,3',5,6-hexachlorobiphemyl	41.7
2,2',3,3',5,6'-hexachlorobiphenyl	41.7
2,2',3,3',6,6'-hexachlorobiphenyl	41.6
2,2',3,4,4',5-hexachlorobiphenyl	41.8
2,2',3,4,4',5'-hexachlorobiphenyl	41.8
2,2',3,4,4',6-hexachlorobiphenyl	40.8
2,2',3,4,4',6'-hexachlorobiphenyl	41.8
2,2',3,4,5,5'-hexachlorobiphenyl	41.8
2,2',3,4,5,6-hexachlorobiphenyl	41.7
2,2',3,4,5,6'-hexachlorobiphenyl	41.7
2,2',3,4,5',6-hexachlorobiphenyl	41.6
2,2',3,4,6,6'-hexachlorobiphenyl	41.6
2,2',3,4',5,5'-hexachlorobiphenyl	41.8
2,2',3,4',5,6-hexachlorobiphenyl	41.6
2,2',3,4',5',6-hexachlorobiphenyl	41.6
2,2',3,4',6,6'-hexachlorobiphenyl	41.6
2,2',3,5,5',6-hexachlorobiphenyl	41.7
2,2',3,5,6,6'-hexachlorobiphenyl	41.6
2,2',4,4',5,5'-hexachlorobiphenyl	41.8
2,2',4,4',5,6'-hexachlorobiphenyl	41.6
2,2',4,4',6,6'-hexachlorobiphenyl	41.5
2,3,3',4,4',5-hexachlorobiphenyl	42.6
2,3,3',4,4',5'-hexachlorobiphenyl	42.6
2,3,3',4,4',6-hexachlorobiphenyl	41.7
2,3,3',4,5,5'-hexachlorobiphenyl	42.6
2,3,3',4,5,6-hexachlorobiphenyl	41.8
2,3,3',4,5'6-hexachlorobiphenyl	41.8
2,3,3',4',5,5'-hexachlorobiphenyl	42.6
2,3,3',4',5,6-hexachlorobiphenyl	41.8
2,3,3',4',5',6-hexachlorobiphenyl	41.8
2,3,3',5,5',6-hexachlorobiphenyl	41.4
2,3,4,4',5,6-hexachlorobiphenyl	40.7
2,3',4,4',5,5'-hexachlorobiphenyl	42.6
2,3',4,4',5',6-hexachlorobiphenyl	41.8

3,3',4,4',5,5'-hexachlorobiphenyl	43.4
2,2',3,3',4,4',5-heptachlorobiphenyl	44.8
2,2',3,3',4,4',6-heptachlorobiphenyl	44.7
2,2',3,3',4,5,5'-heptachlorobiphenyl	44.8
2,2',3,3',4,5,6-heptachlorobiphenyl	44.7
2,2',3,3',4,5,6'-heptachlorobiphenyl	44.7
2,2',3,3',4,5',6-heptachlorobiphenyl	44.7
2,2',3,3',4,6,6'-heptachlorobiphenyl	44.7
2,2',3,3',4',5,6-heptachlorobiphenyl	44.6
2,2',3,3',5,5',6-heptachlorobiphenyl	44.7
2,2',3,3',5,6,6'-heptachlorobiphenyl	44.7
2,2',3,4,4',5,5'-heptachlorobiphenyl	44.7
2,2',3,4,4',5,6-heptachlorobiphenyl	44.7
2,2',3,4,4',5,6'-heptachlorobiphenyl	44.7
2,2',3,4,4',5',6-heptachlorobiphenyl	44.7
2,2',3,4,4',6,6'-heptachlorobiphenyl	44.6
2,2',3,4,5,5'6-heptachlorobiphenyl	44.7
2,2',3,4,5,6,6'-heptachlorobiphenyl	44.6
2,2',3,4',5,5',6-heptachlorobiphenyl	44.7
2,2',3,4',5,6,6'-heptachlorobiphenyl	44.6
2,3,3',4,4',5,5'-heptachlorobiphenyl	45.6
2,3,3',4,4',5,6-heptachlorobiphenyl	44.7
2,3,3',4,4',5',6-heptachlorobiphenyl	44.8
2,3,3',4,5,5',6-heptachlorobiphenyl	44.8
2,3,3',4',5,5',6-heptachlorobiphenyl	44.8
2,2',3,3',4,4',5,5'-octachlorobiphenyl	48.1
2,2',3,3',4,4',5,6-octachlorobiphenyl	48.0
2,2',3,3',4,4',5,6'-octachlorobiphenyl	48.0
2,2',3,3',4,4',6,6'-octachlorobiphenyl	47.8
2,2',3,3',4,5,5',6-octachlorobiphenyl	48.0
2,2',3,3',4,5,5',6'-octachlorobiphenyl	48.0
2,2',3,3',4,5,6,6'-octachlorobiphenyl	47.9
2,2',3,3',4,5',6,6'-octachlorobiphenyl	46.4
2,2',3,3',5,5',6,6'-octachlorobiphenyl	47.9
2,2',3,4,4',5,5',6-octachlorobiphenyl	48.1
2,2',3,4,4',5,6,6'-octachlorobiphenyl	48.2
2,3,3',4,4',5,5',6-octachlorobiphenyl	48.1
2,2',3,3',4,4',5,5',6-nonachlorobiphenyl	51
2,2',3,3',4,4',5,6,6'-nonachlorobiphenyl	50.8
2,2',3,3',4,5,5',6,6'-nonachlorobiphenyl	50.9
decachlorobiphenyl	53.9
1,1,1-trichloro-2,2-(4-clc6h4)ethane	38.1
1,1-dichloro-2,2-(4-clc6h4)ethene	39.7
1,1-dichloro-2,2-(4-clc6h4)ethane	35.2
1-chloronaphthalene	21.9
2-chloronaphthalene	21.5

1,2-dichloronaphthalene	27.1
1,3-dichloronaphthalene	27.4
1,4-dichloronaphthalene	27.7
1,5-dichloronaphthalene	27.8
1,6-dichloronaphthalene	28
1,7-dichloronaphthalene	27.9
1,8-dichloronaphthaalene	28.7
bromobenzene	16.9
1,4-dibromobenzene	23.7
1,3,5-tribromobenzene	28
4-bromotoluene	20.4
2-bromonaphthalene	24.1
4-bromo-p-terphenyl	39.9
iodobenzene	18.4
4-iodotoluene	21.1
4-iodoethylbenzene	26.1
o-fluorochlorobenzene	16.7
m-fluorochlorobenzene	16.4
p-fluorochlorobenzene	16.7
o-fluoroiodobenzene	19.9
m-fluoroiodobenzene	19.8
p-fluoroiodobenzene	19.2
4-chlorobenzotrifluoride	23.4
methylphenylether	11.9
ethylphenylether	14.9
4-propylanisole	20.9
2,4,6-tribromoanisole	26.0
diphenylether	3.3
4-chlorodiphenylether	29.9
4-bromodiphenylether	30.3
1,3-dimethoxybenzene	10.0
1,4-dimethoxybenzene	8.7
tetrachloro-1,4-dimethoxybenzene	24.5
styrene oxide	6.3
benzaldehyde	6.0
4-methylbenzaldehyde	8.3
2,5-dimethylbenzaldehyde	12.4
2-methoxybenzaldehyde	5.1
cinnamaldehyde	6.3
2,6-dichlorobenzaldehyde	16.9
phenylacetaldehyde	6.7
acetophenone	6.5
4-methylacetophenone	9.3
4-methoxyacetophenone	15.5
benzylmethylketone	3.5
benzoylacetone	11.2

1,1,1-trifluorobenzoylacetone	9.9
benzil	15.6
anthraquinone	16.7
2-methylanthraquinone	19.4
phenanthraquinone	8.4
9-fluorenone	18.7
methadone	13.3
mitoxantrone	-41.1
1-chloroanthraquinone	17.9
7-hydroxyflavone	3.8
4',5,7-trihydroxyflavone	-20.9
2',3,4',5,7-pentahydroxyflavone	-46.1
3',5,7-trihydroxy-4-methoxyflavone	-12.7
5,7-dihydroxy-4'-methoxyflavone	-5.6
3',4',5,7-tetrahydroxyflavone	-27.2
2,3-dihydro-4',5,7-trihydroxyflavone	-19.5
3,3',4',5,5,',7-hexahydroxyflavone	-51.8
rutin	-91.2
naringin	-73.0
3,3',4',5,7-pentahydroxyflavone	-36.6
catechin	-56
epicatechin	-59.6
hesperidin	0.0
methyl benzoate	10.4
ethyl benzoate	13.7
methyl 4-dimethylaminobenzoate	10.6
methyl 3-nitrobenzoate	6.4
methyl 4-nitrobenzoate	6.8
methyl 4-bromobenzoate	15.3
methyl 4-iodobenzoate	16.8
methyl 3,4-dichlorobenzoate	18.7
methyl 3,5-dinitrobenzoate	4.7
methyl 2-nitro-4-chlorobenzoate	9.1
methyl 3-nitro-4-chlorobenzoate	9.5
phenyl acetate	2.9
diphenyl carbonate	15.5
dimethyl phthalate	2.6
diethyl phthalate	6.2
dibutyl phthalate	23.0
benzonitrile	5.4
4-methoxybenzonitrile	5.5
phenylacetonitrile	5.3
1,2-dicyanobenzene	0.0
1,3-dicyanobenzene	-1.5
1,4-dicyanobenzene	-0.4
2,6-dichlorobenzonitrile	14.3

aniline	-2.1
o-toluidine	2.2
p-toluidine	2.1
4-ethylaniline	4.9
4-propylaniline	8.7
4-isopropylaniline	6.8
4-butylaniline	11
2-chloroaniline	6.1
3-chloroaniline	3.7
4-chloroaniline	3.2
4-bromoaniline	4.5
2-methoxyaniline	1.9
3-methoxyaniline	-1.9
4-methoxyaniline	-3
2-nitroaniline	1.3
3-nitroaniline	-3.5
4-nitroaniline	-6.5
3-aminoacetophenone	-2.2
4-aminoacetophenone	0.6
methyl 4-aminobenzoate	-3.4
ethyl 4-aminobenzoate	0.6
propyl 4-aminobenzoate	3.9
isopropyl 4-aminobenzoate	2.6
butyl 4-aminobenzoate	6.8
isobutyl 4-aminobenzoate	6.4
pentyl 4-aminobenzoate	11.8
o-phenylenediamine	-9.7
m-phenylenediamine	-14.3
3,5-dinitroaniline	-2.5
2-methyl-3,5-dinitroaniline	-1.4
4-methyl-3,5-dinitroaniline	-1.7
2,4-dimethylaniline	4.3
2,6-dimethylaniline	7.3
3,4-dimethylaniline	4.6
4-pentylaniline	15
4-hexylaniline	18.7
n-methylaniline	5.9
n,n-dimethylaniline	12.4
n,n-diethylaniline	16.6
1-naphthylamine	6.6
2-naphthylamine	6.6
di(4-aminophenyl)methane	-9.7
4,4'-diaminobiphenyl	-6.8
3,4-dichloroaniline	6.6
2,4,5-trichloroaniline	14.7
phenylazide	14.6

azobenzene	20.5
trans-3,3',4,4'-tetrachloroazobenzene	38.5
nitrobenzene	8.8
2-nitrotoluene	12.8
3-nitrotoluene	14.7
4-nitrotoluene	13.1
1-chloro-2-nitrobenzene	11.5
4-nitroanisole	8.0
4-(chloromethyl)nitrobenzene	11.6
1,2-dinitrobenzene	4.6
1,3-dinitrobenzene	4.1
1,4-dinitrobenzene	3.6
2,4-dinitrotoluene	6.7
2,6-dinitrotoluene	8.1
3,4-dinitrotoluene	7.8
1-nitronaphthalene	16.9
2-nitronaphthalene	18.5
3-nitrobiphenyl	20.5
benzamide	-13.1
n-methylbenzamide	-9.0
n-ethylbenzamide	-5.5
n,n-dimethylbenzamide	-5.0
n,n-diethyl-4-methylbenzamide	5.9
1-naphthaleneacetamide	-6.6
diphenamid	5.7
acetanilide	-8.9
n-methylacetanilide	-2.3
4-methoxyacetanilide	-10.6
4-ethoxyacetanilide	-6.3
2-(4-bromophenyl)-n,n-dimethylacetamide	3.4
lorcainide	0.0
phthalimide	-7.9
n-methylphthalimide	2.9
n-phenylurethane	2.2
salicylamide	-10.6
benzoic acid	-2.5
2-methylbenzoic acid	1.7
3-methylbenzoic acid	-0.9
4-methylbenzoic acid	-2.3
3,4-dimethylbenzoic acid	2.9
4-ethylbenzoic acid	2.6
4-butylbenzoic acid	8.4
2-fluorobenzoic acid	-6
3-fluorobenzoic acid	-1.8
4-fluorobenzoic acid	-1.8
2-chlorobenzoic acid	-3.8

3-chlorobenzoic acid	0.3
4-chlorobenzoic acid	0.9
2-bromobenzoic acid	-4.2
3-bromobenzoic acid	2.1
4-bromobenzoic acid	3.1
2-iodobenzoic acid	-3.8
4-iodobenzoic acid	3.7
2-methoxybenzoic acid	-6.6
4-methoxybenzoic acid	-5.5
2-nitrobenzoic acid	-12.5
3-nitrobenzoic acid	-7.5
4-nitrobenzoic acid	-5.4
4-aminobenzoic acid	-20.0
4-n,n-dimethylaminobenzoic acid	-2.1
4-formylbenzoic acid	-6.2
3,5-dinitrobenzoic acid	-7.5
2,4-dichlorobenzoic acid	-0.7
3,4-dichlorobenzoic acid	5.0
2-chloro-5-nitrobenzoic acid	-2.5
2,3-difluorobenzoic acid	-6.2
2,4-difluorobenzoic acid	-5.1
2,5-difluorobenzoic acid	-5.6
2,6-difluorobenzoic acid	-9.7
3,4-difluorobenzoic acid	-3
3,5-difluorobenzoic acid	-1.5
acetylsalicylic acid	-13.1
4-aminosalicylic acid	-19.3
5-aminosalicylic acid	-18.8
5,6,7,8-tetrahydronaphthalene-1-carboxylic acid	6.7
5,6,7,8-tetrahydronaphthalene-2-carboxylic acid	6.6
phenylacetic acid	-8.6
3-phenylpropanoic acid	-5.6
4-phenylbutanoic acid	-2.6
flurbiprofen	5.9
a-methyl-4-isobutylphenylacetic acid	9.8
diclofenac	10.0
ketoprofen	0.4
phthalic acid	-22.6
isophthalic acid	-13
trans-cinnamic acid	-2.2
3,4-dihydroxycinnamic acid	-27.2
1-naphthoic acid	1.8
1-naphthylacetic acid	-1.5
3-methoxy-4-hydroxycinnamic acid	-13.9
4-hydroxycinnamic acid	-14.3
3,5-dimethoxy-4-hydroxycinnamic acid	-17.7

tranilast	-3.1
naproxen	2.3
phenol	-6.2
2-methylphenol	-0.5
3-methylphenol	-1.7
4-methylphenol	-1.1
2,3-dimethylphenol	0.3
2,4-dimethylphenol	2.1
2,5-dimethylphenol	2.5
2,6-dimethylphenol	4.7
3,4-dimethylphenol	1.2
3,5-dimethylphenol	1.5
2,3,6-trimethylphenol	8.1
2,4,6-trimethylphenol	8.4
2-ethylphenol	1.1
3-ethylphenol	0.9
4-ethylphenol	1.4
2-propylphenol	4.8
4-propylphenol	4.2
4-isopropylphenol	3.5
4-butylphenol	7.6
2-sec-butylphenol	7.1
4-sec-butylphenol	6
2-tert-butylphenol	7.4
3-tert-butylphenol	6.6
4-tert-butylphenol	6.6
2-isopropyl-5-methylphenol	8.8
2-methyl-5-isopropylphenol	7.1
4-pentylphenol	12
4-tert-pentylphenol	8.6
2,6-diethylphenol	11.6
4-nonylphenol	25.8
4-tert-octylphenol	20.2
2-benzylphenol	7.5
3-benzylphenol	7.0
4-benzylphenol	7.0
2-phenylphenol	5.1
3-phenylphenol	0.0
4-phenylphenol	0.0
2-allylphenol	3.0
2-tert-butyl-6-methylphenol	9.8
2-tert-butyl-5-methylphenol	10.2
2-tert-butyl-4-methylphenol	10.2
1,2,3,4-tetrahydro-1-naphthol	1.6
1,2,3,4-tetrahydro-2-naphthol	-0.9
2-fluorophenol	-2.5

3-fluorophenol	-4.2
4-fluorophenol	-4.0
2-chlorophenol	4.8
3-chlorophenol	-0.5
4-chlorophenol	-2.2
2-bromophenol	6.2
3-bromophenol	-0.1
4-bromophenol	-0.5
2-iodophenol	2.3
3-iodophenol	-0.9
4-iodophenol	-0.5
2,3,5,6-tetrafluorophenol	-3
pentafluorophenol	-2.5
2,3-dichlorophenol	7.2
2,4-dichlorophenol	7.1
2,5-dichlorophenol	7.4
2,6-dichlorophenol	8.0
3,4-dichlorophenol	0.3
3,5-dichlorophenol	2.2
2,4,5-trichlorophenol	8.0
2,4,6-trichlorophenol	11.6
3,4,5-trichlorophenol	5.8
2,4-dibromophenol	9.0
3,5-dibromophenol	5.5
2,4,6-tribromophenol	12.4
4-methyl-3,5-dibromophenol	8.6
4-chloro-3-methylphenol	1.8
2-trifluoromethylphenol	2.1
3-trifluoromethylphenol	1.6
4-trifluoromethylphenol	1.9
2-methoxyphenol	2.1
3-methoxyphenol	-5.7
4-methoxyphenol	-7.9
2-hydroxybenzaldehyde	6.2
3-hydroxybenzaldehyde	-10.9
4-hydroxybenzaldehyde	-7.0
4-hydroxyacetophenone	-12.8
3-cyanophenol	-10.8
4-cyanophenol	-11.6
2-aminophenol	-14.1
3-aminophenol	-19.2
4-aminophenol	-20.0
2-nitrophenol	8.0
3-nitrophenol	-7.8
4-nitrophenol	-11.0
2,3-dinitrophenol	-5.1

2,4-dinitrophenol	2.3
2,5-dinitrophenol	3.2
2,6-dinitrophenol	-1.5
3,4-dinitrophenol	-14.5
3,5-dinitrophenol	-10.4
2,4,6-trinitrophenol	-6.7
2-methyl-4,6-dinitrophenol	7.1
2-sec-butyl-4,6-dinitrophenol	13.9
2,4-dinitro-6-aminophenol	-5.0
2,4-dinitroresorcinol	-3.3
2-isopropyl-4,6-dinitrophenol	13.5
2,6-dichloro-4-nitrophenol	1.9
2-hydroxybenzoic acid	-3.8
3-hydroxybenzoic acid	-12.2
4-hydroxybenzoic acid	-10.9
5-bromosalicylic acid	1.8
catechol	-15.7
resorcinol	-21.3
hydroquinone	-23
methyl 4-hydroxybenzoate	-6.4
ethyl 4-hydroxybenzoate	-2.7
propyl 4-hydroxybenzoate	1.0
benzyl 4-hydroxybenzoate	1.7
methyl 2-hydroxybenzoate	11.4
methyl 3-hydroxybenzoate	-6.0
ethyl 2-hydroxybenzoate	14.0
propyl 2-hydroxybenzoate	18.7
butyl 2-hydroxybenzoate	22.6
phenyl 2-hydroxybenzoate	13.5
chlorohydroquinone	-8.4
4-methyl-1,2-dihydroxybenzene	-13.1
capsaicin	-0.4
2-hydroxy-n-phenylbenzamide	4.7
2-hydroxybenzamide	-10.2
4-hydroxy-3-methoxybenzaldehyde	-4.0
3-hydroxy-4-methoxybenzaldehyde	-5.0
2-hydroxy-3-methoxybenzaldehyde	3.2
2-methoxy-4-allylphenol	4.5
2-methoxy-4-propenylphenol	9.4
3,4,5-trihydroxybenzoic acid	-37.4
3-methoxy-4-hydroxybenzoic acid	-16.2
4-hydroxypropriophenone	-8.5
butyl 4-hydroxybenzoate	3.4
4-hydroxyacetanilide	-25.5
2-amino-4-nitrosophenol	-15.2
4-nitrosophenol	-7.1

2-methyl-4-nitrosophenol	-3.4
1-naphthol	2.3
2-naphthol	1.4
3,4,5-tribromocatechol	-11.2
tetrabromocatechol	-6.2
3-bromo-2-nitroso-1-naphthol	4.3
1,2,3-trihydroxybenzene	-28.6
1,2,4-trihydroxybenzene	-31.7
1,3,5-trihydroxybenzene	-31.8
dienestrol	-4.1
hexestrol	0.5
diethylstilbestrol	0.4
4-allyl-2,6-dimethoxyphenol	3.3
2,3-dihydroxybenzoic acid	-15.7
2,4-dihydroxybenzoic acid	-16.1
2,5-dihydroxybenzoic acid	-21.2
3,4-dihydroxybenzoic acid	-23.9
3,5-dihydroxybenzoic acid	-31.4
isopropyl-4-formylphenoxyacetate	3.1
2,6-dibromo-4-cyanophenol	3.5
3,5,3',5'-tetrabromobisphenola	24.7
1,3-dihydroxynaphthalene	-15.6
1,5-dihydroxynaphthalene	-13
1,7-dihydroxynaphthalene	-14.4
podophyllotoxin	-9.8
methyl vanillate	0.5
rutin	-40.0
4,4'-isopropylidenediphenol	-5.4
dopamine	-28.1
tetracycline	-59.7
minocycline	-41.4
doxorubicin	-44.3
daunorubicin	-27
clindamycin	-23.8
zorubicin	-42.3
mithramycin	-122
benzyl alcohol	-5.0
4-methylbenzyl alcohol	-3.7
2-hydroxybenzyl alcohol	-14.2
2-(4-methylphenyl)-propan-2-ol	-0.1
2-phenylethanol	-2.0
3-phenylpropan-1-ol	1.3
3-phenoxypropan-1-ol	-1.3
2-benzyloxyethanol	-6.0
oxybutynin	16.0
nebivolol	3.9

bupranolol	6.2
f-isopropylbupranolol	0.8
iodocyanopindolol	-1.5
pindolol	-1.4
chloramphenicol	-18.7
norephedrin	-11.7
ephedrine	-4.2
albuterol	57.6
f-isopropylpenbutalol	9.2
tramadol, equatorial	9.2
carazolol	1.0
esmolol	-3.6
atenolol	-25.2
labetalol	-14.4
propranolol	3.4
thiophenol	11.5
phenylmethylsulfide	15.2
methylphenylsulfoxide	-8.3
cyclohexylmethyl phenylsulfoxide	7.1
diphenylsulfoxide	4.1
methylphenylsulfone	-4.8
diphenylsulfone	6.8
4-methylsulfonylphenol	-22.8
phenylthiourea	-12.4
benzenesulfonamide	-16.9
n-methylbenzenesulfonamide	-6.6
n,n-dimethylbenzenesulfonamide	0.7
1-(phenylsulfonyl)pyrrolidine	6.4
2-methylbenzenesulfonamide	-13.0
3-methylbenzenesulfonamide	-13.5
4-methylbenzenesulfonamide	-13.6
tolbutamide	-7.4
furosemide	-19.1
probenecid	-2.5
bumetanide	-13.5
hydrochlorothiazide	-33.3
sulfachlorpyridazine	-24.5
triphenylphosphine	30.4
triphenylphosphineoxide	3.3
triphenylphosphate	20.7
tri-o-tolylphosphate	29.2
tri-m-tolylphosphate	29.2
tri-p-tolylphosphate	29.2
fenchlorphos	29.2
isazofos	9.6
isofenphos	15.6

n,2,4,6-tetranitro-n-methylaniline	0.7
pentaerythritol tetranitrate	3.5
glycerol trinitrate	3.3
2-propylfuran	17.1
2-butylfuran	20.6
2-pentylfuran	24
2-hexylfuran	27.2
2-heptylfuran	30.8
furan	7.1
2-methylfuran	10.6
2,5-dimethylfuran	13.2
2-ethylfuran	13.9
furfuryl acetate	1.8
furfuryl alcohol	-10.5
2-furaldehyde	-2.9
5-methylfurfural	-0.6
2-acetylfuran	-2.2
methyl 2-furoate	1.7
5-hydroxymethyl-2-furaldehyde	-18.3
carbofuran	1.4
phthalan	7.5
cannabinol	26.5
d8tetrahydrocannabinol	29.4
dibenzofuran	24.5
dihydrocoumarin	4.5
coumarin	2.5
maltol	-9.5
phthalide	-1.1
fluorescein	-18.1
diacetylfluorescein	7.0
warfarin	0.2
6-methyl-4h-chromen-4-one	6.5
2-phenyl-4h-chromen-4-one	16.7
1,4-dihydro-3h-isochromen-3-one	-1.1
1,3-benzodioxole	10.6
piperonal	1.0
xanthene	26.5
luteolin	-15.4
1,3-dioxolane	-6.3
2-methyl-1,3-dioxolane	-4.2
2-ethyl-1,3-dioxolane	-0.9
2,2-dimethyl-1,3-dioxolane	-1.6
2-methyl-2-ethyl-1,3-dioxolane	2.1
4-methyl-1,3-dioxolane	-4.2
4,5-dimethyl-1,3-dioxolane	-1.4
myristin	12.2

1,3-dioxepane	-3.8
paraldehyde	0.2
tropolone	-2.1
6-isopropyltropalone	6.4
pyridine	-2.4
2-methylpyridine	0.1
3-methylpyridine	0.7
4-methylpyridine	0.1
2,3-dimethylpyridine	2.9
2,4-dimethylpyridine	2.5
2,5-dimethylpyridine	3.0
2,6-dimethylpyridine	2.2
3,4-dimethylpyridine	2.8
3,5-dimethylpyridine	3.8
2-ethylpyridine	3.8
3-ethylpyridine	4.1
4-ethylpyridine	3.7
2-methyl-5-ethylpyridine	8.5
2-chloropyridine	3.8
3-chloropyridine	4.8
2-bromopyridine	4.3
3-bromopyridine	6.8
2-methoxypyridine	5.1
2-acetylpyridine	0.2
3-acetylpyridine	-6.7
4-acetylpyridine	-5.3
2-cyanopyridine	-3.2
3-cyanopyridine	-4.5
4-cyanopyridine	-2.2
3-formylpyridine	-5.4
4-formylpyridine	-3.1
2-aminopyridine	-10.3
4-aminopyridine	-11.6
2-n,n-dimethylaminopyridine	5.0
4-nitropyridine	-3.7
2-pyridinemethanol	-11.5
2-pyridineethanol	-11.4
2-pyridinepropanol	-10.9
2-pyridinebutanol	-9.5
2-pyridinepentanol	-8.4
phenazopyridine	-4.7
nicotine	0.2
chlorpheniramine	13
brompheniramine	11.6
zimeldine	11.1
cotinine	-13.2

diantipyrylmethane	-8.5
hexyldiantipyrylmethane	5.8
1-benzylpyridin-2(1h)-one	0.2
1-(3-chlorobenzyl)pyridin-2(1h)-one	5.2
1-[4-(trifluoromethyl)benzyl]pyridin-2(1h)-one	8.1
1-(biphenyl-4-ylmethyl)pyridin-4(1h)-one	5.1
2,2'-bipyridine	6.2
nicotinic acid	-23
isoniazid	-21.5
amrinone	-25.3
nicardipine	10.4
nimodipine	7.8
niflumic acid	-0.7
4-methylpyridine thiocyanate complex	-17.5
azocane	7.6
2-piperidineethanol	-11.5
piperidine	-3.7
n-methylpiperidine	2.7
2-methylpiperidine	0.5
3-methylpiperidine	0.4
4-methylpiperidine	0.5
cocaine	5.1
atropine	-5.5
fentanyl	13.5
haloperidol	8.7
domperidone	-1.5
methylphenidate	-0.2
n-methyl-2-pyridone	-13
adenosine	-42.7
1-methylquinolin-2(1h)-one	2.4
quinoline	7.2
isoquinoline	9.6
8-hydroxyquinoline	8.0
2-methyl-8-hydroxyquinoline	10.6
4-methyl-8-hydroxyquinoline	11.2
5-chloro-8-hydroxyquinoline	14.4
5,7-dichloro-8-hydroxyquinoline	16.7
2-methyl-5,7-dichloro-8-hydroxyquinoline	19.5
5,7-dibromo-8-hydroxyquinoline	19.3
grepafloxacin	-31.3
quinine	1.8
quinidine	1.8
fleroxacin	-32.3
1,5-naphthylridine	-2.2
pyrrole	-2.4
2-methylpyrrole	0.0

n-furfurylpyrrole	9.1
2-acetylpyrrole	-6.6
2-pyrrolecarbaldehyde	-5.1
methyl 2-pyrrolecarboxylate	-3.1
inaperisone	18.4
n-methyl-2-pyrrolidinone	-10.9
n-benzyl-2-pyrrolidinone	-0.3
n-trifluoroacetylindole	18.3
d-valerolactam	-15.9
n-methyl-d-valerolactam	-5.4
indole	-1.9
3-methylindole	11.2
6-chloroindole	8.3
indomethacin	6.8
carbazole	14.8
tryptophan	-41.2
carbamazepine	-5.5
mianserin	17.9
papaverine	13.0
diphenhydramine	15.1
4-acetyl-1-phenyl-3-methyl-5-pyrazolone	5.5
4-propionyl-1-phenyl-3-methyl-5-pyrazolone	9.0
4-butanoyl-1-phenyl-3-methyl-5-pyrazolone	12.7
4-pentanoyl-1-phenyl-3-methyl-5-pyrazolone	16.3
4-hexanoyl-1-phenyl-3-methyl-5-pyrazolone	19.6
4-heptanoyl-1-phenyl-3-methyl-5-pyrazolone	23.4
4-octanoyl-1-phenyl-3-methyl-5-pyrazolone	27.0
4-nonanoyl-1-phenyl-3-methyl-5-pyrazolone	30.6
4-decanoyl-1-phenyl-3-methyl-5-pyrazolone	34.8
4-benzoyl-1-phenyl-3-methyl-5-pyrazolone	12.8
imidazole	-13.2
n-methylimidazole	-6.6
4-(2-aminoethyl)imidazole	-23.5
dipyridamole	-28.2
1-benzyl-1h-imidazole	3.3
2-trifluoromethyl-5,6-dichlorobenzimidazole	12.6
2-trifluoromethyl-4,5,6,7-tetrachlorobenzimidazole	21.4
2-trifluoromethylbenzimidazole	5.0
benzimidazole	-13.6
1-methylbenzimidazole	-5.1
1,3-dimethyl-1,3-dihydro-2h-benzimidazol-2-one	2.9
albendazole sulfoxide	-14.7
pyrazine	-6.3
2-methylpyrazine	-4.1
2,3-dimethylpyrazine	-2
2,5-dimethylpyrazine	-1.3

trimethylpyrazine	0.2
tetramethylpyrazine	1.6
2-ethylpyrazine	-0.6
2,3-diethylpyrazine	4.0
2,5-diethylpyrazine	6.1
2-methyl-5-ethylpyrazine	2.5
2-isobutylpyrazine	6.8
2-methyl-3-isobutylpyrazine	6.8
2-fluoropyrazine	-2.3
2-chloropyrazine	1.1
2-methoxypyrazine	1.0
2-ethoxypyrazine	4.1
2-propoxypyrazine	7.8
2-carbmethoxypyrazine	-7.8
2-carbethoxypyrazine	-4.8
2-acetylpyrazine	-5.2
2-cyanopyrazine	-4.8
2-vinylpyrazine	-2.9
quinoxaline	3.1
1,10-phenanthroline	4.5
2-dimethylaminopyrimidine	1.7
5-dimethylaminopyrimidine	-3.5
pyrimidine	-8.6
2-methylpyrimidine	-6.3
5-methylpyrimidine	-5.8
2-fluoropyrimidine	-3.9
5-fluoropyrimidine	-4.9
2-chloropyrimidine	-2.6
5-chloropyrimidine	-2.0
2-bromopyrimidine	-1.8
5-bromopyrimidine	-0.5
2-methoxypyrimidine	-4.3
2-ethoxypyrimidine	-1.3
5-ethoxypyrimidine	-2.3
methyl 2-pyrimidinecarboxylate	-13.4
methyl 5-pyrimidinecarboxylate	-7.0
ethyl 2-pyrimidinecarboxylate	-11.5
quinazoline	0.8
2-methylquinazolin-4(3h)-one	-8.4
ra12165	6.4
2-n-methylaminopyrimidine	-7.1
2-n-phenylaminopyrimidine	-5.0
2-thiomethoxypyrimidine	1.2
2-cyanopyrimidine	-5.1
trimethoprim	-11.1
4-phenylpyrimidine	6.5

1-methyl-1,3-dihydro-2h-indol-2-one	0.3
1-ethyl-1,3-dihydro-2h-indol-2-one	3.1
2-methyl-2,3-dihydro-1h-isoindol-1-one	-2.1
riboflavin	-58
lamivudine	-27.4
cytosine	-31.8
antipyrine	-11.3
aminopyrine	-6.2
piperazine	-23.2
n-methylpiperazine	-16.8
n,n-dimethylpiperazine	-10.3
2,5-dimethylpiperazine	-16.7
hydroxyzine	6.7
quinuclidine	3.9
chlordiazepoxide	-1.8
2-hydroxybenzotriazole	-18.7
benzotriazole	-11.6
1,2,4-triazole	-30.0
flutriafol	2.5
tebuconazole	8.6
hexaconazole	6.5
3-amino-1,2,4-triazole	-31.4
azosemide	-29.4
purine	-24.6
adenine	-28.4
6-chloropurine	-19.8
methotrexate	-59.2
didanosine	-33.4
2,4,5-trimethyloxazole	3.9
2,5-diphenyl-1,3-oxazole	26.8
benzoxazole	5.5
2,1-benzisoxazole	4.8
morpholine	-11.2
n-methylmorpholine	-7.8
n-formylmorpholine	-15.1
n-ethylmorpholine	-1.8
scopolamine	-12.6
uracil	-22.5
thymine	-19
1,3-dimethyluracil	-11.7
bromacil	-1.5
xanthine	-31.5
theophylline	-21.5
theobromine	-24.9
caffeine	-11.8
flumazenil	-6.8

guanine	-34.6
hypoxanthine	-28.3
acyclovir	-40.4
acycloviroacetyl	-33.3
acyclovirn2acetyl	-40.8
acyclovirnodiacetyl	-34.2
desciclovir	-29.0
descicloviroacetyl	-22.3
desciclovirnacetyl	-36.6
desciclovirnodiacetyl	-31.1
morphine	-14.4
codeine	-9.9
strychnine	-0.9
galantamine	-14.1
trazodone	6.0
uridine	0.0
oxycodone	-20.5
mdma	5.3
milnacipran	-1.1
nalbuphine	-23.6
phenylbutazone	14.2
diazepam	10.2
piroxicam	3.6
tenoxicam	-15.5
oxazepam	0.9
temazepam	2.7
clotiazepam	0.0
cefuroxime	-39.9
nevirapine	-7.3
thiophene	10.2
2-methylthiophen	13.1
2-formylthiophene	2.2
2-formyl-3-methylthiophene	7.4
dibenzothiophene	26.4
thianthrene	25.9
1,1,1-trifluoro-4(2-thienyl)-4-mercaptobut-3-en-2-one	17.5
xylazine	11.4
thiazole	-2.5
2-acetylthiazole	-1.2
benzothiazole	7.9
mercaptobenzothiazole	9.0
nizatidine	-24.1
phenothiazine	17.1
promazine	22.9
chlorpromazine	28.1
desmonomethylpromazine	18.6

oxacillin	-14.0
penicilling	-12.1
nafcillin	-2.6
cephalothin	-33.0
cabenicillin	-29
piperacillin	-39.7
cloxacillin	-12.2
dicloxacillin	-9.2
oxacillin	-14
amoxicillin	-59.4
floxacillin	-10.4
ampicillin	-44.8
cefazolin	-36.6
cinoxacin	-15.6
ciprofloxacin	-39.5
cephalexin	-50.1
pipemidic acid	-44.6
enoxacin	-36.2
lomefloxacin	-35.4
ofloxacin	-31
pefloxacin	-25.5
amiloride	-31.8
amitriptyline	25.2
physostigmine	-3.0
stobadine	2.2
skb2	-15.4
skb7	10.3
skb8	23.3
skb19	-7.0
skb20	-19.5
skb24	-10
skb26	0.9
skb30	-1.0
skb31	8.8
skb34	4.7
skb36	16
skb41	20.2
2-(2-dimethylamino)pyridine	-1.4
2-(2-aminoethyl)thiazole	-10.2
skf101468	5.6
2-(2-methylamino)pyridine	-7.0
desipramine	20.5
progesterone	9.9
hydrocortisone	-22
prednisolone	-20.2
cholesterol	58.4

betulin	30.2
11a-hydroxy-16a,17a-epoxyprogesterone	-3.6
estrone	1.4
estratriol	-16.3
digitoxin	-51.2
cholesterol acetate	67.6
11-b-methoxyestradiol	-13.3
11-b-ethylestradiol	0.0
16a-fluoroestradiol	-3.4
16a-fluoro-11b-methoxyestradiol	-15.2
16a-fluoro-11b-ethylestradiol	3.3
16a-fluoro-17a-ethynylestradiol	0.0
norethisterone	-2.6
etonogestrel	-2.5
norgestrel	1.0
gestodene	-1.1
ethinylestradiol	-6.8
om08	-21.3
org34694	0.7
org36410	8.4
org30659	-6.6
org4325	0.0
org4060	2.8
org32540	0.0
glucose	-57.4
sucrose	-79.5
jg-13	-6.9
jg-14	-4.7
jg-15	-17.1
jg-16	-23.4
jg-17	0.0
atrazine	3.9
simazine	0.4
propazine	7.2
terbuthylazine	9.9
cyanazine	-4.3
simetryn	4.7
ametryn	8.6
prometryn	12.3
terbutryn	13.6
dimethametryn	17
dipropetryn	15.3
aziprotryn	10.8
methoprotryn	6
atraton	1.3
terbumeton	7.5

secbumeton	4.3
prometon	5.6
chlorpropamide	-5.5
diflubenzuron	16.8
hexaflumuron	22.1
phenylurea	-17.6
3-cf3-1-phenyl-3,3-dimethylurea	-0.5
m-chloro-1-phenyl-3,3-dimethylurea	-3.2
p-chloro-1-phenyl-3,3-dimethylurea	-2.6
3,4-dichloro-1-phenyl-3,3-dimethylurea	0.5
3-me-4-chloro-1-phenyl-3,3,-dimethylurea	2.2
3-chloro-4-methoxy-1-phenyl-3,3-dimethylurea	-5.1
1-phenyl-1,3,3-trimethylurea	1.4
1,3-diphenylthiourea	-1.9
n-phenylbenzohydroxamic acid	0.4
n-phenyl-2-methylbenzohydroxamic acid	3
n-m-tolylbenzohydroxamic aid	3.6
n-o-tolyl-2-methylbenzohydroxamic acid	6.8
n-p-tolyl-2-methylbenzohydroxamic acid	6.1
n-phenyl-4-ethoxybenzohydroxamic acid	2.7
n-p-tolyl-4-ethoxybenzohydroxamic acid	7.4
n-o-tolyl-4-chlorobenzohydroxamic acid	8.8
n-m-chlorophenyl-4-methoxybenzohydroxamic acid	4.1
n-p-chlorophenyl-4-bromobenzohydroxamic acid	10.4
n-m-chlorophenylbenzohydroxamic acid	6.1
n-p-tolyl-phenoxyacetohydroxamic acid	2.5
fccp	4.7
cccp	4.1
barbituric acid	-27.6
5-methyl-5-ethylbarbituric acid	-19.7
5,5-diethylbarbituric acid	-13
5-ethyl-5-propylbarbituric acid	-13.2
5-allyl-5-ethylbarbital	-15.2
5,5-diallylbarbital	-12.4
1,5-dimethyl-5-cyclohexenylbarbituric acid	-2.7
5-ethyl-5-phenylbarbital	-13.5
ftorafur	-17.6
thiopental	1.5
phenoxyacetic acid	-11.1
2,4-dichlorophenoxyacetic acid	-5.5
carbaryl	3.0
fenoxycarb	17.6
pirimicarb	3.0
dimethirimol	3.5
ethirimol	-0.1
bupirimate	-4.0

cyprodinil	18.4
propachlor	10.9
acetochlor	10.0
metolachlor	9.9
flufenamic acid	14.9
isoxaben	10.3
metalaxyl	-0.7
ethylfumesate	7.5
haloxyfop	-2.9
amlodipine	-0.7
zidovudine	-22.8
azoxystrobin	6.9
kresoxim-methyl	17.7
5,5-diphenylhydantoin	-11.3
procainamide	-20.7
procaine	-2.3
lidocaine	6.2
ropivacaine	1.7
bupivacaine	5.9
prilocaine	-4.7
tetracaine	9.3
chloroprocaine	3.9
rifampin	-71.7
pyridine-n-oxide	-15.1
2,4,6-trimethylquinoline-1-oxide	3.1
n,n-dimethyl-2-phenylethylamineoxide	-12
n,n-dimethyl-3-phenylpropylamineoxide	-10.9
2,2-difluoropropane	6.4
1,1,2,2,3-pentafluoropropane	9.9
1,1,1,2,2-pentafluoropropane	11.5
1,1,2,2,3,3-hexafluoropropane	5.1
1,1,1,3,3,3-hexafluoropropane	9.6
1,1,1,2,2,3,3-heptafluoropropane	11.4
1,1,1,2,3,3,3-heptafluoropropane	12.3
1,1,2,3,4,4-hexafluorobutane	3.5
1,1,1,2,2,3,4,4-octafluorobutane	11.3
1,1,1,2,3,4,4,4-octafluorobutane	10.2
1,1,2,2,3,3,4,4-octafluorobutane	12.2
bctc	7.6
thiobctc	18.9
icilin	-5.1
frescolatml	11.4
frescolatmga	6.1
ws3	6.7
coolingagent10	-4.5
pmd38	-8.3

coolantp	6.7
ws23	-5.8
hydroxycitronellal	-8.6
capsazepine	-5
a-copaene	37
germacrene d	41
a-ylangene	37.1
a-cubebene	36.7
a-muurolene	39.1
a-farnesene	37.5
1,8-cineole	14.3
geranial	16
camphor	10.8
b-ionone	17.6
dihydro-b-ionone	20.8
carveol	4.3
dihydrocarveol	5.9
linalool	10.3
hydroxycitronellal	-4.2
menthol	12.0
borneol	7.5
geranyl acetone	18.3
benzylamine	-1.5
2-phenylethylamine	-3.6
n,n-dimethyl-3-phenylpropylamine	12.0
4-phenylbutylamine	6.0
n-methylbenzylamine	1.5
4-methyl-n-methylbenzylamine	4.6
4-methyl-n-ethylbenzylamine	7.1
4-methyl-n-propylbenzylamine	10.8
4-methyl-n-butylbenzylamine	14.0
4-methyl-n-pentylbenzylamine	18.6
4-methyl-n-hexylbenzylamine	22.6
4-methyl-n-heptylbenzylamine	24.7
deprenyl	10.8
4-chloroamphetamine	5.7
amphetamine	2.9
fluoxetine	19.3
verapamil	5.9
diphenylamine	16.7
ch3ocf2chclf	9.4
ch3ocf2chbrf	11
ch3ocf2chcl2	11.5
ch3ocf2cf2cf2cf3	21.8
ch3ocf2cf(cf3)2	21.8
ch2focf2chf2	4.9

chf2och2cf3	7.0
ch2fochfcf3	5.5
ch2focf2ch(cf3)2	19.3
cclf2och2cf3	15.7
chf2ochclcf3	16.8
chf2ocf2chclf	5.7
chf2ochfcf3	8.3
chf2ocf2cbrclf	20.3
chf2ocbrclcf3	19.5
cclf2ochfcf3	16.4
chf2occlfcf3	16.0
chf2ocf2cclf2	15.0
cclf2ochclcf3	18.8
chf2occl2cf3	18.1
cclf2ocf2chclf	18.5
chf2ocf2ccl2f	18.1
chf2ocf2cf3	14.5
cf3ochfcf3	13.9
chf2oc(f)(chf2)cf3	12.7
cclf2occlfcf3	23.9
cclf2ocf2cclf2	24.6
cclf2occl2cf3	26.3
cclf2ocf2ccl2f	26.6
ccl2focf2cclf2	26.7
2-difluoromethylperfluorooxolane	16.0
cis-2,5-bis(fluoromethyl)perfuorooxolane	12.7
trans-2,5-bis(fluoromethyl)perfluorooxolane	13.5
cis-2,5-bis(difluoromethyl)perfluorooxolane	16.5
trans-2,5-bis(difluoromethyl)perfluorooxolane	17.2
cis-3,4-difluoro-2,2-bis(trifluoromethyl)oxetane	12.0
trans-3,4-difluoro-2,2-bis(trifluoromethyl)oxetane	15.0
2,2,3-trifluoro-4,4-bis(trifluoromethyl)oxetane	18.3
2,2-difluoro-4,4-bis(trifluoromethyl)oxetane	16.3
3,3-difluoro-2,2-bis(trifluoromethyl)oxetane	16.3
4-fluoro-2,2-bis(trifluoromethyl)oxetane	12.1
3-fluoro-2,2-bis(trifluoromethyl)oxetane	12.0
2,2-bis(trifluoromethyl)oxetane	11.2
n-nitrosodimethylamine	-9.4
n-nitrosodiethylamine	-2.3
n-nitrosodipropylamine	4.6
n-nitrosodiisopropylamine	3.7
n-nitrosodibutylamine	11.5
n-nitrosodiisobutylamine	10.7
n-nitrosodipentylamine	18.4
n-nitrosomethylethylamine	-5.9
n-nitrosomethylpropylamine	-2.2

n-nitrosomethylbutylamine	1.3
n-nitrosomethylpentylamine	5.0
n-nitrosoethylpropylamine	0.9
n-nitrosoethylbutylamine	4.5
n-nitrosopropylbutylamine	7.9
n-nitrosobutylpentylamine	14.2
1-nitroso-2-naphthol	7.7
2-nitroso-1-naphthol	1.4
acetaldehyde oxime	-11.7
propanal oxime	-8.1
butanal oxime	-4.6
isobutanal oxime	-6.5
acetone oxime	-10.8
butanone oxime	-7.0
cyclopentanone oxime	-8.7
cyclohexanone oxime	-4.5
hydrogen sulfide	1.3
ethylthiol	7.1
propylthiol	10.4
butylthiol	13.3
dimethylsulfide	4.9
diethylsulfide	11.1
dipropylsulfide	17.5
diisopropylsulfide	15.2
methylethylsulfide	7.2
bis(benzoxazolyl-2-methyl)sulfide	17.0
diethyldisulfide	16.2
methylethyldisulfide	12.5
methylpropyldisulfide	16.1
diallyldisulfide	18.5
allylmethyldisulfide	13.7
dimethyltrisulfide	11.4
diethyl malonate	1.4
dibutyl succinate	16.9
dibutyl glutarate	18.4
dimethyl adipate	-1.0
diethyl adipate	3.4
4-(1-adamantyl)phenol	15.9
2-(1-adamantyl)-4-cresol	18.7
2-cyclohexyloxyphenol	12.4
3-cyclohexyloxyphenol	7.4
4-cyclohexyloxyphenol	7.0
2-methoxy-4-vinylphenol	5.4
2-cyclohexylmethyloxyphenol	14.7
3-cyclohexylmethyloxyphenol	11.5
4-cyclohexylmethyloxyphenol	11.5

4-hydroxypropriophenone	-7.7
4-hydroxybutyrophenone	-4.5
4-hydroxypentanophenone	-1.1
2-hydroxybenzophenone	17.5
3-hydroxybenzophenone	-0.6
4-hydroxybenzophenone	-0.5
4,4'-dihydroxybenzophenone	-18
triclosan	16.8
serotonin	-23.6
totarol	34.7
5-hydroxyindole	-9.0
6-hydroxyquinoline	-5.5
ferruginol	36.6
8-hydroxyjulolidine	1.2
4-chlorocatechol	-11.3
3,5-dichlorocatechol	-7.4
3,4,5-trichlorocatechol	-3.5
tetrachlorocatechol	-0.2
dibenzo-p-dioxin	26.2
2,3,7,8-tetrachlorodibenzo-p-dioxin	38.9

Table S7 Experimental free energy of tranfer from *n*-hexadecane to water (ΔG°_{expt})

3.2 Free Energy of Transfer and the Number of Alkyl Hydrogens

Figure S9 explores the relationship between free energy of transfer from *n*-hexadecane to water (ΔG°_{calc}) and the number of alkyl hydrogens (N_H) for a homologous series of compounds containing a single functional group but a varying alkyl chain length.

(a) alkenes

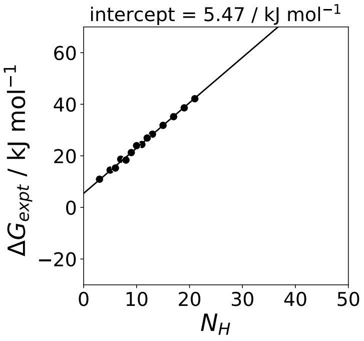

(b) alkenes

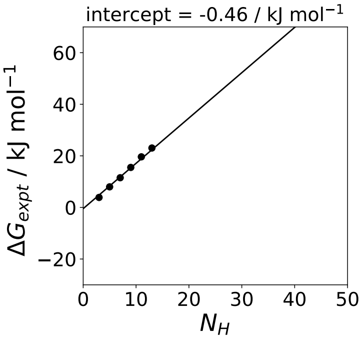

(c) primary amines

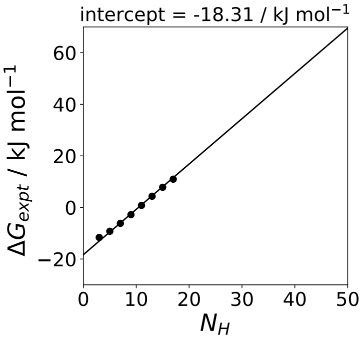

(d) secondary amines

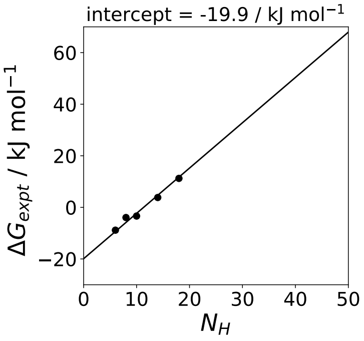

(e) tertiary amines

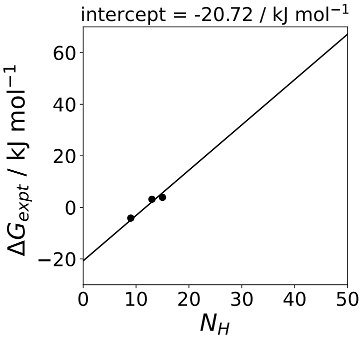

(f) nitriles

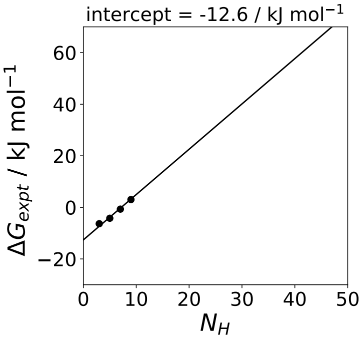

(g) ethers

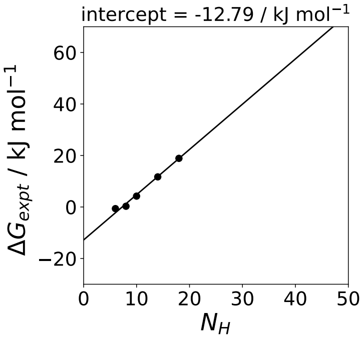

(h) alcohols

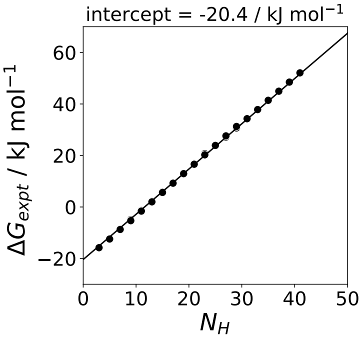

(i) aldehydes

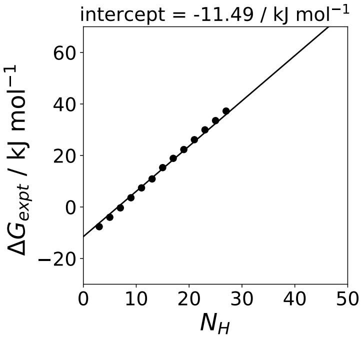

(j) ketones

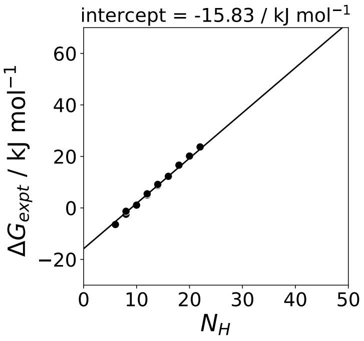

(k) esters

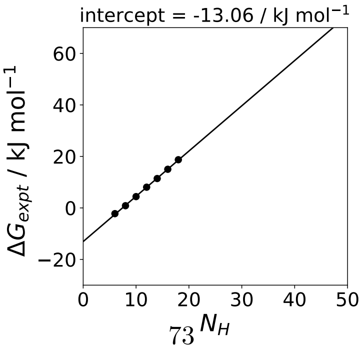

(l) formate esters

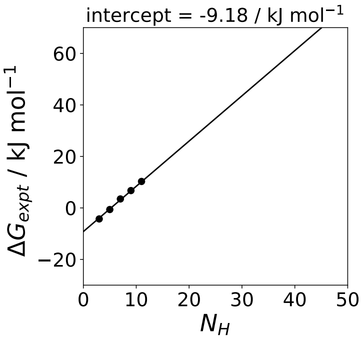

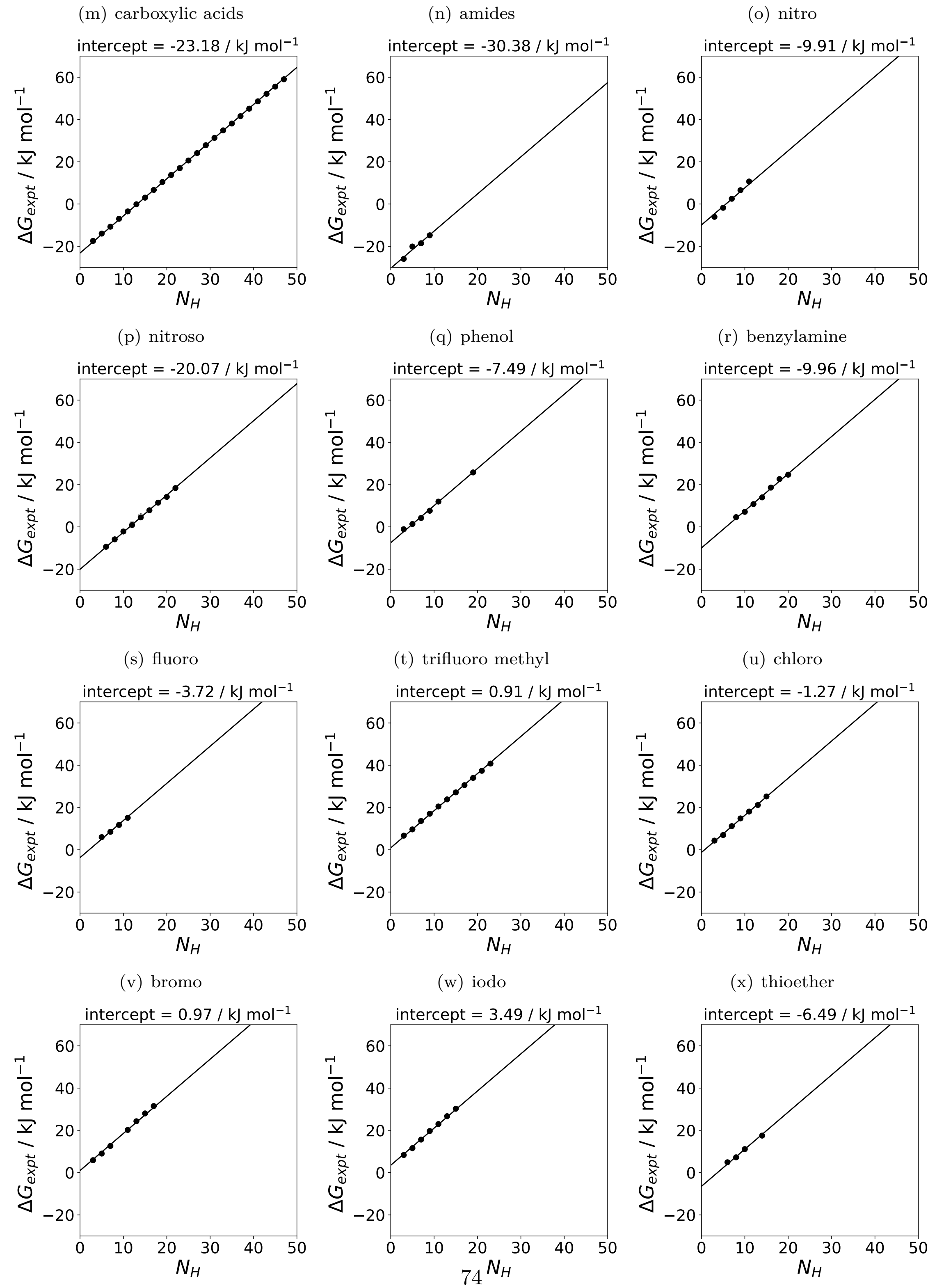

Figure S9 ΔG°_{expt} vs N_H for a range of homologous series of functional groups. The lines are the lines of best fit keeping the slope constant amongst the different functional groups (slope = +1.8 kJ mol^{-1})

3.3 f value for Various Atom Types

The surface area of the patch of surface associated with each atom is calculated using the local density of points given by Equ. S6.

$$A_{atom} = \sum_p \frac{1}{N_{neigh}} \pi r^2 \tag{S6}$$

where p is every MEP point in the patch, N_{neigh} is the number of points are within a radius r = 0.1 Å of the point p considered. The algorithm to find N_{neigh} was the kDtree query ball algorithm implemented with SciPy (*27*, *28*).

The non-polar surface area assigned to an AIP (A_{np}) can be obtained from Equ. S7, where A_{atom} is the surface area of the associated atom on the 0.002 e bohr^{-3} electron density isosurface, A_{AIP} is 9.35 Å^2 and N_p is the number of polar AIPs.(*29*)

$$A_{np} = A_{atom} - N_p A_{AIP} \tag{S7}$$

4 Host-guest complexes AIP pairings

4.1 Tables of host-guest interactions

AIP 1		AIP2		f value	$\Delta\Delta G$ kJ mol^{-1}
type	value	type	value		
H.N	3.7	N.ar	-6.7	1.0	-13.7
H.N	3.7	N.ar	-6.8	1.0	-13.6
O.2.am	-7.2	H.N	2.8	1.0	-8.7
O.2.am	-6.9	H.N	2.8	1.0	-7.9

Table S8 AIP contacts for the host-guest complex shown in Figure 16 of the main text and free energy contributions to binding in chloroform solution.

AIP 1		AIP2		f value	$\Delta\Delta G$ kJ mol^{-1}
type	value	type	value		
H.N	2.8	C.ar	-1.2	0.5	-0.4
H.N	2.8	C.ar	-0.9	0.5	-0.3
H.N	2.8	C.ar	-1.3	0.5	-0.5
H.N	2.8	C.ar	-1.1	0.5	-0.4
O.2.am	-6.4	H.N	2.4	1.0	-5.4
O.2.am	-6.3	H.N	2.3	1.0	-4.6
O.2.am	-6.6	H.N	2.3	1.0	-4.8
O.2.am	-6.6	H.N	2.2	1.0	-4.1

Table S9 AIP contacts for the host-guest complex shown in Figure 17 of the main text and free energy contributions to binding in chloroform solution.

AIP 1		AIP2		f value	$\Delta\Delta G$ kJ mol^{-1}
type	value	type	value		
H.soft	1.4	C.ar	-1.3	0.5	-1.3
H.soft	1.4	C.ar	-1.1	0.5	-1.3
H.soft	1.3	C.ar	-1.0	0.5	-1.4
H.soft	1.3	C.ar	-0.9	0.5	-1.4
H.soft	1.3	C.ar	-1.6	0.5	-1.3
H.soft	1.3	C.ar	-1.3	0.5	-1.4
H.soft	1.4	C.ar	-1.6	0.5	-1.2
H.soft	1.4	C.ar	-1.2	0.5	-1.3
N.pl3.am	-0.4	C.ar	-1.6	0.5	-1.6
N.pl3.am	0.5	C.ar	-1.0	0.5	-1.9
N.pl3.am	-0.4	C.ar	-1.3	0.5	-1.8
N.pl3.am	0.5	C.ar	-1.6	0.5	-1.7
H.N	2.8	C.ar	-1.2	0.5	0.8
H.N	2.8	C.ar	-0.9	0.5	1.0
H.N	2.8	C.ar	-1.3	0.5	0.8
H.N	2.8	C.ar	-1.1	0.5	0.8
O.2.am	-6.4	H.N	2.4	1.0	0.8
O.2.am	-6.3	H.N	2.3	1.0	1.1
O.2.am	-6.6	H.N	2.3	1.0	1.2
O.2.am	-6.6	H.N	2.2	1.0	1.4

Table S10 AIP contacts for the host-guest complex shown in Figure 17 of the main text and free energy contributions to binding in water.

References

1. G. Beggiato, G. G. Aloisi, U. Mazzucato, *J. Chem. Soc., Faraday Trans. 1* **70**, 628–634 (1974).
2. C. Laurence, J. Graton, M. Berthelot, M. J. El Ghomari, *Chem. A Eur. J.* **17**, 10431–10444 (2011).
3. L. J. Andrews, R. M. Keefer, *J. Org. Chem.* **52**, 2690–2694 (1987).
4. A. M. Lamsabhi, W. Bouab, M. Esseffar, M. Alcamí, M. Yáñez, J.-L. M. Abboud, *New J. Chem.* **25**, 509–517 (3 2001).
5. A. El Firdoussi, M. Esseffar, W. Bouab, A. Lamsabhi, J.-L. M. Abboud, O. Mó, M. Yáñez, *New J. Chem.* **27**, 1741–1747 (12 2003).
6. A. El Firdoussi, M. Esseffar, W. Bouab, J.-L. M. Abboud, O. Mó, M. Yáñez, *J. Phys. Chem. A* **108**, 10568–10577 (2004).
7. H.-P. Sleper, R. Paetzold, *Z. Phys. Chem.* **255**, 1125–1135 (1974).
8. E. Augdahl, P. Klaeboe, *Acta Chem. Scand.* **18**, 18 (1964).
9. J.-M. Dumas, M. Gomel, M. Guerin, in *Halides, Pseudo-Halides and Azides (1983)* (John Wiley & Sons, Ltd, 1983), chap. 21, pp. 985–1020, ISBN: 9780470771723.

10. J. Dumas, C. Geron, A. Kribii, M. Lakraimi, *Canadian journal of chemistry* **62**, 2634–2640 (1984).

11. M. G. Sarwar, B. Dragisic, L. J. Salsberg, C. Gouliaras, M. S. Taylor, *J. Am. Chem. Soc* **132**, 1646–1653 (2010).

12. C. Laurence, M. Queignec-Cabanetos, B. Wojtkowiak, *J. Chem. Soc., Perkin Trans. 2*, 1605–1610 (12 1982).

13. M. C. Storer, C. A. Hunter, *Chem. Soc. Rev.* **51**, 10064–10082 (2022).

14. M. Berthelot, F. Besseau, C. Laurence, *Eur. J. Org. Chem.* **1998**, 925–931 (1998).

15. J. Graton, C. Laurence, M. Berthelot, J.-Y. Le Questel, F. Besseau, E. D. Raczynska, *J. Chem. Soc., Perkin Trans. 2*, 997–1002 (5 1999).

16. C. Ouvrard, M. Berthelot, C. Laurence, *J. Chem. Soc., Perkin Trans. 2*, 1357–1362 (7 1999).

17. H. Zhong, J. P. Bowen, *J. Am. Chem. Soc.* **129**, 5780–5780 (2007).

18. L. Cordella, P. Foggia, C. Sansone, M. Vento, *IEEE Trans. Pattern Anal. Mach. Intell* **26**, 1367–1372 (2004).

19. A. Hagberg, P. Swart, D. Chult, *Proc. Python Sci. Conf.* (2008).

20. Ł. Mentel, *Mendeleev - A Python package with properties of chemical elements, ions, isotopes and methods to manipulate and visualize periodic table.* Version 0.14.0, 2021, (`https://github.com/lmmentel/mendeleev`).

21. C. B. Barber, D. P. Dobkin, H. Huhdanpaa, *ACM Trans. Math. Softw.* **22**, 469–483 (1996).

22. C. H. J. H. S. Park, *Expert Syst. Appl.* **36**, 3336–3341 (2009).

23. F. Pedregosa, G. Varoquaux, A. Gramfort, V. Michel, B. Thirion, O. Grisel, M. Blondel, P. Prettenhofer, R. Weiss, V. Dubourg, J. Vanderplas, A. Passos, D. Cournapeau, M. Brucher, M. Perrot, E. Duchesnay, *J. Mach. Learn. Res.* **12**, 2825–2830 (2011).

24. D. P. Reynolds, M. C. Storer, C. A. Hunter, *Chem. Sci.* **12**, 13193–13208 (2021).

25. Y. Chung, F. H. Vermeire, H. Wu, P. J. Walker, M. H. Abraham, W. H. Green, *J. Chem. Inf. Model.* **62**, 433–446 (2022).

26. M. H. Abraham, P. L. Grellier, R. A. McGill, *J. Chem. Soc., Perkin Trans. 2*, 797–803 (1987).

27. J. L. Bentley, *Commun. ACM* **18**, 509–517 (1975).

28. P. Virtanen, R. Gommers, T. E. Oliphant, M. Haberland, T. Reddy, D. Cournapeau, E. Burovski, P. Peterson, W. Weckesser, J. Bright, S. J. van der Walt, M. Brett, J. Wilson, K. J. Millman, N. Mayorov, A. R. J. Nelson, E. Jones, R. Kern, E. Larson, C. J. Carey, İ. Polat, Y. Feng, E. W. Moore, J. VanderPlas, D. Laxalde, J. Perktold, R. Cimrman, I. Henriksen, E. A. Quintero, C. R. Harris, A. M. Archibald, A. H. Ribeiro, F. Pedregosa, P. van Mulbregt, SciPy 1.0 Contributors, *Nature Methods* **17**, 261–272 (2020).

29. C. S. Calero, J. Farwer, E. J. Gardiner, C. A. Hunter, M. Mackey, S. Scuderi, S. Thompson, J. G. Vinter, *Phys. Chem. Chem. Phys.* **15**, 18262–18273 (41 2013).
